# Late-Stage Functionalisation of Polycyclic (*N*-Hetero-) Aromatic Hydrocarbons by Detoxifying CYP5035S7 Monooxygenase of the White-Rot Fungus *Polyporus arcularius*

**DOI:** 10.3390/biom11111708

**Published:** 2021-11-17

**Authors:** Nico D. Fessner, Christopher Grimm, Wolfgang Kroutil, Anton Glieder

**Affiliations:** 1 Institute of Molecular Biotechnology, Graz University of Technology, NAWI Graz, 8010 Graz, Austria; a.glieder@tugraz.at; 2 Institute of Chemistry, University of Graz, NAWI Graz, 8010 Graz, Austria; christopher.grimm@uni-graz.at (C.G.); wolfgang.kroutil@uni-graz.at (W.K.); 3 Field of Excellence BioHealth, University of Graz, 8010 Graz, Austria

**Keywords:** *P. arcularius*, late-stage functionalisation, CYP5035, detoxification, polycyclic aromatic hydrocarbons

## Abstract

Functionalisation of polycyclic aromatic hydrocarbons (PAHs) and their *N*-heteroarene analogues (NPAHs) is a tedious synthetic endeavour that requires diverse bottom-up approaches. Cytochrome P450 enzymes of white-rot fungi were shown to participate in the fungal detoxification of xenobiotics and environmental hazards via hydroxylation of PAH compounds. In this paper, the recently discovered activity of the monooxygenase CYP5035S7 towards (N)PAHs was investigated in detail, and products formed from the substrates azulene, acenaphthene, fluorene, anthracene, and phenanthrene by whole-cell biocatalysis were isolated and characterised. The observed regioselectivity of CYP5035S7 could be explained by a combination of the substrate’s electron density and steric factors influencing the substrate orientation giving insight into the active-site geometry of the enzyme.

## 1. Introduction

On the one hand, polycyclic aromatic hydrocarbons (PAHs) are toxic environmental contaminants and carcinogenic and mutagenic hazards [1,2]; but on the other, they are materials with properties suitable for the preparation of innovative optoelectronic devices such as organic light-emitting diodes (OLEDs) and organic photovoltaic cells (PVC) [3,4,5]. However, PAHs themselves are unprocessable [6] and heteroaromatic doping or functionalisation of the their molecular structures is indispensable to allow highly versatile scaling of the bandgap [4,5,7,8,9]. The inherent lack of polarity of PAHs and the low reactivity of arene C-H bond necessitates tedious “bottom-up” syntheses for the introduction of functional groups [10]. Traditional synthetic strategies ranging from the Scholl reaction to transition-metal catalysis require either high temperatures and/or toxic metal catalysts [11] weighing down the environmental bill. In contrast, direct C-H bond functionalisation methods allow the use of the naturally occurring non-functionalised substrates [12,13,14]. Therefore, cytochrome P450 enzymes (P450s) offer themselves as an alternative sustainable solution to such dilemma as they are renowned for their capacity of regioselective late-stage C-H bond activation [15].

Additionally, PAHs are often structural motifs of natural products [16] and many *N*-heterocyclic analogues are essential constituents of a large percentage of FDA-approved drugs [17]. Therefore, the biocatalytic functionalisation of such (N)PAHs is of particular value for the pharmaceutical industry [18].

In 1991 evidence was found that P450s of the model white-rot fungus *Phanerochaete chrysosporium* contribute towards the bioremediation of PAHs [19]. Almost two decades later, specific P450 monooxygenases of the same fungus were identified and expressed to better characterise their PAH-degrading activities [20,21]. However, their functional proficiency towards these compounds was rarely studied in greater detail or put into synthetic context [22,23,24,25,26,27].

In a recent study, CYP5035S7 from the white-rot fungus *Polyporus arcularius* was expressed in *Pichia pastoris* (*Komagataella phaffii*) and found active towards common (N)PAHs such as indole and phenanthrene [28]. Hence in this study, the synthetic potential of CYP5035S7 towards (N)PAH functionalisation was investigated in greater detail by screening a range of different compounds and characterising the corresponding products of some of them formed upon scaling up to semi-preparative conversion of selected substrates in whole-cell biotransformations.

## 2. Materials and Methods

Solvents and chemicals were purchased in best available purity and used as received without further purification from Sigma-Aldrich/Merck (Steinheim/Darmstadt, Germany), VWR International (Fontenay-sous-Bois, France), Carl Roth GmbH (Karls- ruhe, Germany) or Fisher Scientific (Loughborough, UK). The same *P. pastoris* strain expressing CYP5035S7 enzyme was used as reported in a previous study [28]. HPLC tubes were bought from Macherey-Nagel (Düren, Germany) and the corresponding caps and inserts from Bruckner Analysentechnik (Linz, Austria). OD measurements were executed with an Eppendorf BioPhotometer plus. NMR spectra were recorded on a Bruker Avance III 300 MHz NMR spectrometer equipped with an autosampler.

### 2.1. Substrate Screening

Protein expression using the *P. pastoris* strain expressing CYP5035S7 monooxygenase, biotransformations for the substrate screening and their analysis by HPLC followed the same protocols and used the same HPLC instrument and column as described previously [28].

### 2.2. Product Isolation

In order to produce sufficient biomass to scale up the reaction for semi-preparative product isolation, cultivations were performed in baffled 2.5 L shake flasks. BMD1 (450 mL, pH 7.4) was inoculated, then 50 mL of BMM10 (pH 7.4) added after 60 h [29] and 5 mL of methanol fed further three times every 12 h. After harvesting and washing cells twice in 50 mM potassium phosphate buffer (pH 7.4), cells were resuspended in the same phosphate buffer until an OD_600_ of 100 was obtained. The resulting cell broth volume was filled into a clean baffled 2.5 L shake flask, a volume of the 100 mM compound stock solution in DMSO was added to get a final substrate concentration of 1 mM and the flask was covered with a porous piece of cloth. The biotransformation was carried out for 17 h at 28 °C, 80% humidity and 120 rpm. To stop the reaction, the broth was centrifuged and the cell pellet washed sequentially with distilled water (1×) and 50 mL of a MeCN/MeOH (1:1; *v*/*v*) solution (2×). Both the aqueous and MeCN/MeOH supernatant were stored in individual glass bottle at 4 °C. The MeCN/MeOH supernatant was concentrated on rotary evaporation and then combined with the aqueous supernatant. Products were extracted using ethyl acetate (3 × 200 mL) and dried over anhydrous sodium sulphate. The organic phase was concentrated to an end volume of 1 mL and injected into the Shimadzu LC-20AP preparative liquid chromatography system equipped with an FRC-10A fraction collector for the isolation of the products by measuring UV absorbance at 254 nm. A Phenomenex Luna^®^ C18(2) (100 Å; 250 × 21.2 mm; 5 μm) reverse-phase column was used. Water (A) and acetonitrile (B) were used for elution at 25 °C and a flow rate of 30 mL/min: 0–2 min: A/B 100/0; 2–20 min: A/B 70/30; 20–33 min: A/B 0/100; 33–38 min A/B 0/100. Products were recovered by extraction with ethyl acetate from the individual fraction. Agilent Technologies 1260 Infinity HPLC system coupled to a 6120 quadruple LC/MS mass detector (MSD) confirmed the mass of each isolated product.

### 2.3. NMR of Products

**Azulene** (**2**, C_10_H_8_, dark blue solid, 6 mg, 30%): ^1^H NMR (300 MHz, CDCl_3_): *δ* = 8.38 (2H, d, J = 9.8 Hz, 4-, 8-H), 7.93 (1H, dd, J = 4.1, 3.7 Hz, 2-H), 7.61 (1H, dd, J = 10.0, 9.8 Hz, 6-H), 7.42 (2H, d, J = 3.7 Hz, 1-, 3-H), 7.18 (2H, dd, J = 10.0, 9.8 Hz, 5-, 7-H).

**1-Hydroxyazulene** (**11**, C_10_H_8_O, green solid, <1 mg, 5%): ^1^H NMR (300 MHz, CDCl_3_): *δ* = 8.36 (1H, d, J = 9.5 Hz, 4- or 8-H), 8.30 (1H, d, J = 9.8 Hz, 4- or 8-H), 7.84 (1H, d, J = 3.7 Hz, 2-H), 7.58 (1H, dd, J = 10.3, 9.0 Hz, 6-H), 7.38 (1H, d, J = 3.9 Hz, 3-H), 7.17–7.08 (2H, m, 5-, 7-H). The two neighbouring singlets corresponding to the most deshielded protons 4 and 8 between 8.30–8.36 ppm signalled a broken symmetry of the product. As compared to the double doublet of proton 2 of **2**, here the same peak was only a doublet implying hydroxylation to have removed one of the neighbouring protons. Furthermore, it was experienced a shielding effect indicating 1-hydroxyazulene to be the product. The COSY spectrum allowed the approximate annotation of the peaks with help of the literature [30].

**Acenaphthene** (**3**, C_12_H_10_, white crystals, 5 mg, 10%): ^1^H NMR (300 MHz, CDCl_3_): *δ* = 7.57 (2H, d, J = 8.2 Hz), 7.40 (2H, dd, J = 8.2, 8.0 Hz, -H), 7.28–7.23 (2H, m, -H), 3.37 (4H, s, H).

**1-Hydroxyacenaphthene** (**12**, C_12_H_10_O, off-white crystals, 2 mg, 4%): ^1^H NMR (300 MHz, CDCl_3_): *δ* = 7.77 (1H, m, 6-H), 7.68 (1H, dd, J = 8.3, 0.7 Hz, 5-H), 7.57–7.54 (2H, m, 7-, 8-H), 7.53 (1H, dd, J = 8.3, 6.9 Hz, 4-H), 7.33 (1H, dd, J = 6.9, 0.7 Hz, 3-H), 5.77 (1H, dd, J = 7.4, 2.3 Hz, 1-H), 3.87 (1H, dd, J = 17.7, 7.4 Hz, 2-H), 3.29 (1H, d, J = 17.7 Hz, 2-H). In comparison to the spectrum of acenaphthene, here each aromatic proton had a different chemical shift splitting the peaks into individual ones. The aliphatic singlet of acenaphthene was split into three individual peaks suggesting hydroxylation to have occurred at position 1. No sufficient amount of product was obtained to determine the precise enantiomer; however, a COSY and HSQC spectrum allowed for proton assignment and corresponding ^13^C chemical shifts. The product structure was confirmed by literature [31].

**1-Acenaphthenone** (**13**, C_12_H_8_O, beige crystals, 5 mg, 10%): ^1^H NMR (300 MHz, CDCl_3_): *δ* = 8.11 (1H, dd, J = 8.1, 0.4 Hz, 8-H), 7.98 (1H, dd, J = 7.0, 0.4 Hz, 6-H), 7.84 (1H, dd, J = 8.4, 0.4 Hz, 5-H), 7.75 (1H, dd, J = 8.3, 7.1 Hz, 7-H), 7.63 (1H, dd, J = 8.4, 6.9 Hz, 4-H), 7.48 (1H, dd, J = 7.0, 0.7 Hz, 3-H), 3.83 (2H, s, 2-Hs). In comparison to the spectrum of acenaphthene, here each aromatic proton had a different chemical shift splitting the peaks into individual ones. There were six peaks for the six aromatic protons, while the singlet at 3.83 ppm only integrated to 2 protons (compared to 4 for **3**). Hence, the alcohol group of **12** was oxidised to a ketone to form this product. The COSY spectrum allowed for proton assignment and an HSQC spectrum provided corresponding ^13^C chemical shifts. The product structure was confirmed by literature [31].

**2-Hydroxyfluorene** (**14**, C_13_H_10_O, white solid, 4 mg, 35%): ^1^H NMR (300 MHz, CDCl_3_): *δ* = 7.69 (1H, d, J = 7.6 Hz, 5-H), 7.65 (1H, d, J = 8.0 Hz, 4-H), 7.51 (1H, d, J = 7.3 Hz, 8-H), 7.34 (1H, dd, J = 7.6, 7.5 Hz, 6-H), 7.24 (1H, ddd, J = 7.5, 7.3, 1.1 Hz, 7-H), 7.03 (1H, br, 1-H), 6.87 (1H, dd, J = 8.0, 2.3 Hz, 3-H), 3.85 (2H, s, 9-H). The presence of a singlet at 3.85 ppm indicated that hydroxylation had not occurred at the reactive position 9. All other protons corresponded to individual, well-defined peaks spread across the area of 6.8 to 7.7 ppm, which implied asymmetry of the aromatic rings. The broad singlet at 7.03 ppm suggested hydroxylation to have isolated a proton from its neighbours and its de-shielded chemical shift could only correspond to position 1, not 4 involved in the crowded bay region [32]. Hence, 2-hydroxyfluorene was identified as the product, which was confirmed by literature data [33]. The COSY spectrum allowed for proton assignment.

**2,7-Dihydroxyfluorene** (**15**, C_13_H_10_O_2_, beige solid, 2 mg, 18%): ^1^H NMR (300 MHz, Acetone-d_6_): *δ* = 8.21 (1H, s, 2-, 7-OH), 7.49 (2H, d, J = 8.2 Hz, 4-, 5-H), 6.98 (2H, br, 1-, 8-H), 6.81 (2H, dd, J = 8.2, 2.3 Hz, 3-, 6-H), 3.74 (2H, s, 9-H). As compared to the one of **14**, the merged peaks in this spectrum clearly implied a symmetric product once more. The singlet at 6.98 ppm again suggested isolated protons as for **14**, and the two doublets (one double doublet but with only one coupling to a neighbouring proton) supported hydroxylation at positions 2 and 7. Hence, 2,7-dihydroxyfluorene was identified as the product and confirmed by literature data [34]. The COSY spectrum allowed for proton assignment.

**Anthracene** (**5**, C_14_H_10_, off-white crystals, 6 mg, 22%): ^1^H NMR (300 MHz, CDCl_3_): *δ* = 8.43 (2H, s, 9-, 10-H), 8.03–8.00 (4H, m, 1-, 4-, 5-, 8-H), 7.48–7.45 (4H, m, 2-, 3-, 6-, 7-H). 

**1-Hydroxyanthracene** (**16**, C_14_H_10_O, yellow solid, 1 mg, 4%): ^1^H NMR (300 MHz, CDCl_3_): *δ* = 8.77 (1H, s, 9-H), 8.39 (1H, s, 10-H), 8.05–8.00 (2H, m, 5-, 8-H), 7.60 (1H, d, J = 8.0 Hz, 4-H), 7.44 (2H, m, 6-, 7-H), 7.30 (1H, d, J = 8.0 Hz, 3-H), 6.79 (1H, d, J = 7.2 Hz, 2-H). This spectrum was a mixture of different substances. Protons 9 and 10 are split up into two singlets as expected for a monohydroxylated product with broken symmetry. Clearly, proton 9 was de-shielded by 0.34 ppm as compared to **5**. Such a large downfield shift already indicated the introduction of the alcohol group at position 1 due to a larger crowding effect [32]. At the same time, one of the outer rings was clearly experiencing a shielding effect by the electron donation of the new hydroxyl functional group. With the help of the corresponding COSY spectrum, literature data, and chemical shift predictions of the individual protons, 1-hydroxyanthracene could be deduced as the product [35,36].

**Anthraquinone** (**17**, C_14_H_10_, black solid, <1 mg, <3%): ^1^H NMR (300 MHz, CDCl_3_): *δ* = 8.34 (4H, m), 7.83–7.80 (4H, m). The presence of only two peaks of the same shape as those from **5** pointed towards a still highly symmetric product. The lack of a singlet for protons 9 and 10 strongly suggested anthraquinone to be the product, confirmed by literature [37].

**1,7-Dihydroxyanthracene** (**18**, C_14_H_10_O_2_, yellow solid, 1 mg, 4%): ^1^H NMR (300 MHz, Acetone-D_6_): *δ* = 9.09 (1H, s, 1- or 2-OH), 8.78 (1H, s, 9-H), 8.60 (1H, m, 1- or 2-OH), 8.36 (1H, d, J = 8.0 Hz, 10-H), 7.95 (1H, d, J = 8.9 Hz, 5-H), 7.49 (1H, d, J = 8.0 Hz, 4-H), 7.35 (1H, d, J = 2.1 Hz, 8-H), 7.22 (2H, m, 3-, 6-H), 6.84 (1H, dd, J = 7.4, 0.8 Hz, 2-H). The key to identification of the product corresponding to this spectrum were the number of individual peaks implying asymmetry of the molecule and the doublet at 7.35 ppm with a small coupling constant indicating an isolated proton (i.e., position 8 or 5) on one of the outer rings by a newly introduced functional group. As the elution time of this product suggested a more polar compound than **16**, dihydroxylation was considered. Indeed, the chemical shifts of protons of one of the outer aromatic rings matched well to those of **16**, and the others to that of 2-hydroxyanthracene reported in the literature—especially the peak at 7.35 ppm was a symbol of identification [36,38]. Due to the small amounts of product mass recovered, no clear differentiation between 1,7- and 1,6-dihydroxyanthracene could be made; however, the resonance structures would activate the pseudo-ortho position 7 of **16**. Therefore, 1,7-dihydroxyanthracene was derived as the product.

**Phenanthrene** (**6**, C_14_H_10_, off-white crystals, 6 mg, 18%): ^1^H NMR (300 MHz, CDCl_3_): *δ* = 8.72 (2H, dd, J = 7.5, 1.3 Hz, 4-, 5-H), 7.92 (2H, dd, J = 7.5, 1.3 Hz, 1-, 8-H), 7.75 (2H, s, 9-, 10-H), 7.67 (4H, ddd, J = 19.6, 7.5, 1.3 Hz, 2-, 3-, 6-, 7-H).

**1-Hydroxyphenanthrene** (**19**, C_14_H_10_O, beige solid, 5 mg, 15%): ^1^H NMR (300 MHz, CDCl_3_): *δ* = 8.68 (1H, dd, J = 8.3, 1.9 Hz, 5-H), 8.30 (1H, d, J = 8.4 Hz, 4-H), 8.17 (1H, d, J = 9.1 Hz, 10-H), 7.92 (1H, dd, J = 7.0, 1.9 Hz, 8-H), 7.78 (1H, d, J = 9.1 Hz, 9-H), 7.68–7.58 (2H, m, 6-, 7-H), 7.49 (1H, dd, J = 8.4, 7.6 Hz, 3-H), 6.99 (1H, dd, J = 7.6, 0.7 Hz, 2-H). Almost all protons corresponded to individual, well-defined peaks spread across the area of 6.9 to 8.8 ppm. Hence, they all had specific, different chemical shifts indicating asymmetry and a functional group at one of the molecules outer aromatic ringers (i.e., one of 1-H to 4-H. Six of the eight peaks were clearly doublets or double doublets with principal coupling to only one proton and strong enough coupling for it to be a neighbouring proton. None of the other peaks was a singlet ruling out protons 2-H and 3-H as the abstracted protons, as this would have isolated either 1-H or 4-H. Protons 4 and 5 are most deshielded in phenanthrene due to crowding [32], which is why hydroxylation at one of these positions would cause an even stronger deshielding. Therefore, hydroxylation must have had occurred at position 1. Annotation of the peaks with each proton could be achieved using COSY and starting with 5-H as the most deshielded peak and 2-H as the most shielded peak due to the electron donation of the neighbouring hydroxyl group. The proton shifts were equivalent to those found in the literature [39,40]. The HSQC spectrum provided ^13^C chemical shifts to the corresponding protons.

**2-Hydroxyphenanthrene** (**20**, C_14_H_10_O, beige solid, 5 mg, 15%): ^1^H NMR (300 MHz, CDCl_3_): *δ* = 8.59 (2H, d, J = 8.5 Hz, 4-, 5-H), 7.86 (1H, dd, J = 7.5, 1.2 Hz, 8-H), 7.72 (1H, d, J = 8.9 Hz, 9-H), 7.63 (1H, dd, J = 7.6, 7.5, 1.2 Hz, 6-H), 7.62 (1H, d, J = 8.9 Hz, 10-H), 7.53 (1H, ddd, J = 7.6, 7.5, 1.2 Hz, 7-H), 7.25–7.21 (2H, m, 1-, 3-H). As compared to the spectrum of phenanthrene, the newly introduced alcohol functional group broke symmetry here splitting up the peaks for 9- and 10-H. At the same time, the two shielded protons at 7.24 ppm must correspond to the two protons next to the hydroxyl group due to its electron donation. Although the peaks of both protons are overlapping, also slightly with the chloroform reference peak, a singlet shape at 7.25 can be deduced indicating hydroxylation to have isolated proton 1. An isolated 4-H would be much more de-shielded. The proton shifts were equivalent to those found in the literature [40].

**1,8-Dihydroxyphenanthrene** (**21**, C_14_H_10_O_2_, beige solid, 1 mg, 3%): ^1^H NMR (300 MHz, CDCl_3_): *δ* = 8.26 (2H, d, J = 8.2 Hz Hz, 4-, 5-H), 8.15 (2H, s, 9-, 10-H), 7.48 (2H, dd, J = 8.2, 7.7 Hz, 3-, 6-H), 7.00 (2H, dd, J = 7.7, 0.7 Hz, 2-, 7-H). As compared to the spectrum of **19**, the spectrum of this product promised symmetry exemplified by the singlet at 8.15 ppm clearly corresponding to 9- and 10-H. Indeed, all peaks had a very similar chemical shift to the hydroxylated aromatic ring of **19**. Hence, this product was identified as 1,8-dihydroxyphenanthrene. The COSY spectrum allowed annotation of the peaks with the corresponding protons.

**Compound X** (**X**, C_14_H_10_O_4_, orange solid, 2 mg, 6%): ^1^H NMR (300 MHz, CDCl_3_): *δ* = 9.16 (1H, d, J = 8.3 Hz, 5-H), 8.16 (1H, d, J = 8.4 Hz, -H), 7.95 (1H, d, J = 8.2 Hz, 8-H), 7.74 (1H, ddd, J = 8.3, 6.9, 1.3 Hz, 6-H), 7.63 (1H, ddd, J = 8.3, 6.9, 1.3 Hz, 7-H), 7.59 (1H, d, J = 8.5 Hz, -H), 3.38 (1H, d, J = 18.2 Hz, 1-H), 3.08 (1H, d, J = 18.2 Hz, 2-H). In this proton spectrum, two doublets in roof-shape in the aliphatic region strongly coupled to each other as confirmed by the COSY spectrum. This suggested the presence a dihydro-diol functional group. Their strong shielding furthermore implied the location not be in the crowded bay region at positions 3 and 4 [32]. These aliphatic dihydro-diol protons did not show any interaction with the remaining aromatic peaks suggesting the modification of two further positions, which was further supported by only six aromatic peaks remaining for the total proton count of this product. In the COSY spectrum, two aromatic protons showed a strong mutual coupling, but no further interaction with any other protons as it would be the case for protons 9- and 10-H. The other four peaks showed good correlation to each other in sequential pattern as expected for protons of aromatic rings of phenanthrene. Ketone as compared to alcohol functional groups at positions 3 and 4 would explain better such downfield shift of H-5 to 9.15 ppm. Hence, this structure was tentatively suggested to be 1,2-dihydroxy-1,2-dihydrophenanthrene-3,4-dione, but requires further analysis for confirmation.

## 3. Results

**Figure 1:**
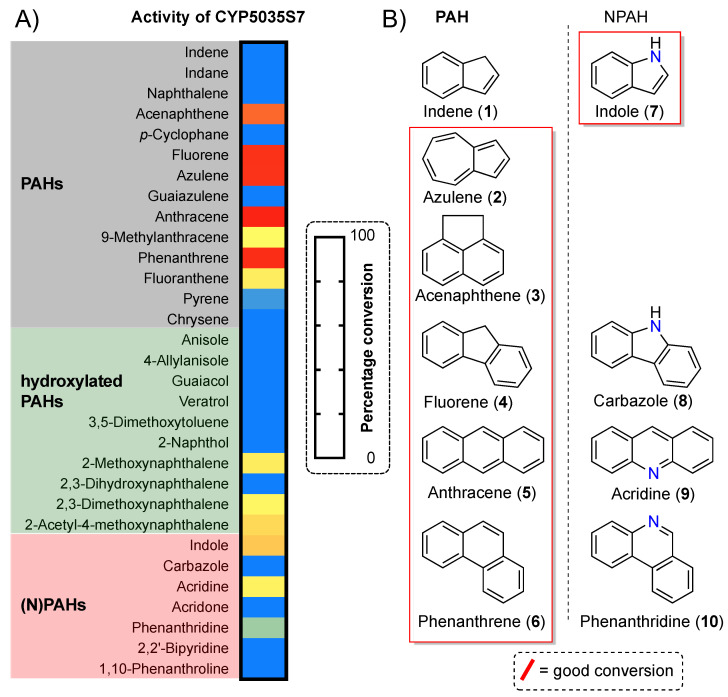
Expansion of the substrate scope of CYP5035S7 towards (N)PAHs. (**A**) The heat map shows the conversion efficiency of CYP5035S7 towards common PAHs, hydroxylated PAHs as well as *N*-containing PAHs (1 mM of **1**, 0.4 mL, OD_600_ = 100, 320 rpm, 17 h, 28 °C). It includes data from our previous publication [28]. (**B**) The contrast between the PAH and NPAH conversions is shown.

We aimed at investigating the substrate scope of CYP5035S7 towards an expanded set of (N)PAH compounds grouped by different criteria such as molecular size, electron density and functionalisation (Figure 1A). The P450 monooxygenase was inactive towards low molecular weight (LMW) PAHs such as indene (**1**) or naphthalene; however, showed high activity towards naphthalene’s isomer azulene (**2**) and the other 2- or 3-cyclic arenes acenaphthene (**3**), fluorene (**4**), anthracene (**5**) and phenanthrene (**6**) with percentage conversions of up to 90%. Higher molecular weight (HMW) PAHs such as fluoranthene, pyrene and chrysene were barely accepted or not all. In light of this variable activity towards PAHs of small ring-size differences, the contrary activity pattern with equivalent NPAHs is curious (Figure 1B): The LMW molecule indole (**7**) was converted well and blue colour formation after biotransformation indicated the formation of indigo (**22**). However, CYP5035S7 showed poor conversion of the larger HMW compounds carbazole (**8**), acridine (**9**), phenanthridine (**10**).

Introducing more nitrogen atoms as in 1,10-phenanthroline killed the little activity completely, which implies a correlation between activity and the number of heteroatoms within PAHs. Functionalised PAHs were only converted if they were bicyclic naphthalene derivatives with a methoxy functional group as seen by the conversion of 2-methoxynaphthalene and 2,3-dimethoxynaphthalene as compared to unaccepted 2-naphtol and 2,3-dihydroxynaphthalene. Such functional features strongly suggested a demethylation ability of this monooxygenase targeting the methyl group of the methoxy moiety for hydroxylation. 

Looking at the versatile nature of the hydroxylation of **6** by P450s of *P. chrysosporium* with monohydroxylation at three different positions reported by Syed et al. [20], we decided to also investigate the product spectra of **6** formed when using CYP5035S7 because the HPLC profile indicated the formation of two monohydroxylated products and a broad peak area at shorter elution times suggested even more polar dihydroxylated derivatives (Figure S1). Lacking access to any authentic references, semi-preparative whole-cell biotransformations were performed to synthesise sufficient product quantities for identification by NMR spectroscopy analysis. Curiously, a different product spectrum was obtained when using a higher cell concentration of OD_600_ = 200 as compared to conditions during the substrate screening (OD_600_ ≈ 100) indicating that the whole-cells of *P. pastoris* must have an influence on the products formed by the P450 enzyme (Figure S2). Hence, an OD_600_ = 100 was used to obtain an authentic product spectrum produced by the P450 biocatalyst. In contrast to more water-soluble substrates such as testosterone [41], the majority of PAH products remained in the cell pellet after biotransformation, which is why after centrifugation a 1:1 mixture of acetonitrile/methanol was used for better product extraction.

**Scheme 1:**
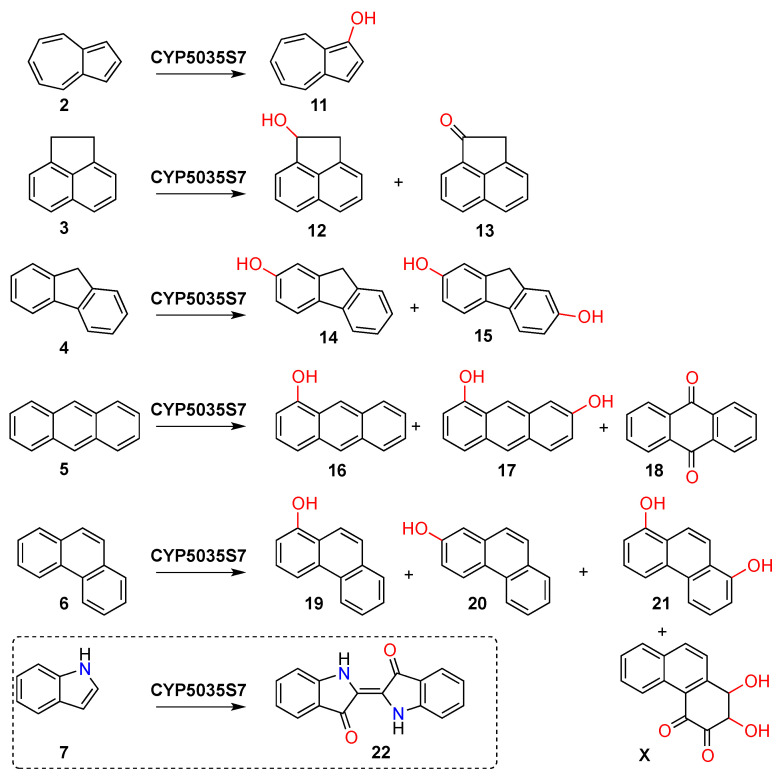
Products identified from the CYP5035S7-catalysed biotransformations of **2**, **3**, **4**, **5**, and **6** employing a *P. pastoris* whole-cell biocatalyst.

**Figure 2:**
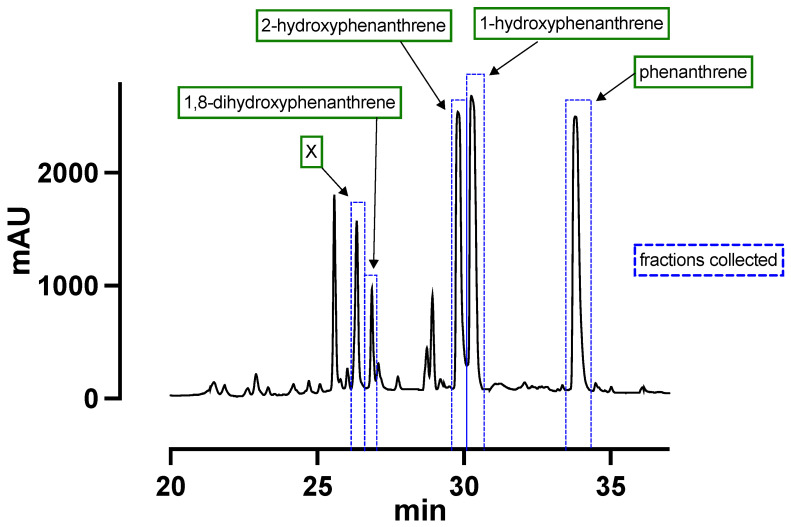
Preparative HPLC profile for product isolation of the scale-up whole-cell biotransformation of **6** employing CYP5035S7 (1 mM of **6**, <100 mL, OD_600_ = 100, 120 rpm, 17 h, 28 °C). The blue dashed boxes indicate the fractions collected and the arrows indicate the corresponding products framed in green that were identified by NMR analysis.

Isolation of the individual products was achieved by an extended HPLC method (38 min vs. 6.5 min) on a preparative HPLC instrument at a flow rate of 30 mL/min (Figure 2). CYP5035S7 hydroxylated **6** at positions 1 and 2 with almost equal rates to form 1- (**19**) and 2- hydroxyphenanthrene (**20**), respectively. However, while a second hydroxyl group was installed in **19** to form 1,8-dihydroxyphenanthrene (**21**) (Scheme 1), no comparable dihydroxylated product could be identified for further oxygenation of **20**. It was surprising to see so many peaks at even shorter elution times indicating further polar products. Although several of them could be isolated, unambiguous identification failed due to the small, isolated amounts as for example for compound X, for which the structure of 1,2-dihydroxy-1,2-dihydrophenanthrene-3,4-dione (**X**) was suggested. 

In addition, we repeated the product analysis and isolation procedure of all the other excellently converted substrates **2** (Figures S3–S5), **3** (Figures S6–S8), **4** (Figures S9–S11) and **5** (Figures S12–S14). Again, a cell concentration OD_600_ of 200 as compared to OD_600_ of 100 was found to produce a different product spectrum for all compounds except **3**, supporting the influence of *P. pastoris*’ whole-cells on further substrate processing. Of these compounds, the CYP5035S7-catalysed biotransformation of **5** also formed a similarly broad product spectrum with several minor polar products as observed for **6**. However, again not all of these could be identified due to the small amounts of product isolated. The various identified products formed from these substrates are displayed in Scheme 1.

## 4. Discussion

The substrate scope accessible by the enzymes of *P. chrysosporium* was nicely summarised by Syed et al. [42] It not only includes (heteroaromatic) PAHs of various ring sizes, but also polychlorinated biphenyls, phenolics, volatile organic solvents, and steroids as well as more exotic compounds such as pesticides, explosives or dyes. For this reason, white-rot fungi can be applied for biological wastewater treatment [43,44,45,46,47].

However, there are few annotations of specific P450 monooxygenases contributing towards such activity, which illustrates the gap that still needs to be filled [48]. Although Syed et al. identified six P450s responsible for PAH degradation in 2010 [20], few studies followed their investigation [21,22,23,24,25,26,27]. Therefore, our study again incites curiosity of P450s capable of regioselective late-stage functionalisation of PAHs via sp^2^-hybridised C-H activation.

The recently discovered CYP5035S7 of *P. arcularius* was found to be active towards common PAH compounds [28], therefore this study aimed at elaborating on its substrate scope with more exotic substrates such as **2** and **3** (Figure 1A) and analysing its regioselectivity in order to outline a more comprehensive activity profile (Scheme 1).

In terms of the aforementioned substrate criteria molecular size, electron density and functionalisation, the electron density combined with steric reasons seemed to be the determining factors for both the enzymatic activity and the regioselectivity towards substrates of suitable size for the active site. CYP5035S7 barely converted the *N*-heterocyclic equivalents **8**–**10** of PAH compounds **4**–**6** most likely due to the electron-withdrawing effect of the *N*-heteroatom resulting in electron-poor aromatic rings (Figure 1B). This hypothesis is supported by the contrasting pattern of **7**, which is fairly electron-rich due to its strong enamine-like, non-basic character [49,50]. 

Relative to LMW arenes such as benzene, indene or naphthalene, medium-sized aromatic rings such as **4**–**6** are more reactive due to their decreasing aromatic character, which is closely related to electron density [51,52]. For example, the aromaticity of **5** is half that of naphthalene. The reduction in aromaticity of linear arenes like **5** affects primarily the terminal rings, while the most dramatic effect can be observed for the central ring in PAHs with a tilted structure such as **6**. In fact, the central ring of **6** is 2.5-fold lower than that of the terminal rings [52], which can be pictured best by Clar’s aromatic π-sextet approach drawing resonance structures with the highest number of π-sextet rings (Figure S15) [53]. These aromaticity calculations are roughly in agreement with the reactive position of various PAH compounds identified by different prediction methods as well as laboratory data summarised by Cheong et al. [54]. They clearly identified position 9 of both **5** and **6** as the most reactive. Indeed, 9-hydroxyphenanthrene was also found to be the dominant product in the biotransformation catalysed by the aforementioned PAH-degrading P450s of Syed et al. found in *P. chrysosporium* [20]. With 3- and 4-hydroxyphenanthrene being their side-products, the regioselective preference of CYP5035S7 in this study towards positions 1 and 2 of **6** is unexpected and a nice addition to the synthetic repertoire of eukaryotic P450s targeting PAHs. Apparently, the active site’s geometry is responsible for the observed reactivity due to a sterically restricted substrate orientation. This hypothesis is supported by the fact that Syed et al.’s P450s were also able to accept sterically demanding HMW arenes such as benzo[a]pyrene [20]. The formation of both **19** and **20** is due to an initial epoxide formation followed by subsequent ring opening [55].

The presence of **18** as a product of the biotransformation of **5** follows the trend of the laboratory data of Cheong et al. [54], although the formation of **16** as the main product with hydroxylation on the terminal ring suggests that activity can be explained here by the predicted aromaticity (Figure S15). For compounds **3** and **4**, the neighbouring carbon to that in the compound’s bay area (positions 4 and 3, respectively) was classified as the preferred reactive site by laboratory data [54], and was hence in disagreement with the regioselectivity observed here. Instead, the prediction with Mulliken and the predicted carbon with the lowest hydroxylated PAH adduct stability could explain the product formation catalysed by CYP5035S7. 

However, it is more likely that the similar substrate sizes of **4** and **5** with **6** causes a comparable effect of the substrate orientation in the active site to take effect. Indeed, the lack of activity towards the central 5-membered ring of **4** despite its low aromaticity according to Clar’s aromatic π-sextet is in line with the disregard of the 9-position of **6** [54]. In light these reactivity explanations, the hydroxylation of the saturated ring of **3** at the activated benzylic position to form **12** [31] is either a bit of an exception or reveals the true activity of CYP5035S7 purely based on electron density without any steric influence. The extraordinary structure and polarity pattern of PAH **2** was studied extensively and the hydroxyated position 1 of the electron-rich five-membered ring of **2** is one of its most electron-rich positions according to resonance delocalisation [56].

Di-hydroxylations of several substrates by CYP5035S7 followed the general reactivity preference for electron-rich substrates considering that an alcohol functionality increases the electron density in the molecule. Simultaneously, in combination with the formation of various still unidentified polar substrates at shorter elution time such product over-hydroxylation (e.g. **X**) strengthens the previously claimed hypothesis that CYP5035 are part of the detoxification machinery of white-rot fungi [22,28].

The introduction of even small side chains as on 9-methylanthracene (vs. anthracene) or guaiazulene (vs. azulene) seemed to hamper the substrate tolerance significantly. Although the broad substrate tolerance towards different substrate classes suggested a promiscuous enzyme [28], the versatility of its large active site seems to be restricted to medium sized molecules and rather inflexible upon small changes in the molecular skeleton.

Just like the *P. pastoris* whole-cell biocatalyst influenced the resultant product spectrum observed in a previous study (testosterone oxidation to androstenedione) [41], the same seemed to be true for the conversion of some of the PAH compounds used here as illustrated by the use of different cell concentrations used (Figures S2, S4, S7, S10 and S13). Although such side-effect allows facile synthesis of follow-up products including C-H functionalisation with subsequent oxidation in a one-pot approach, it also outlines the severe disadvantages of using whole-cell bioconversions: (i) possible undesired product consumption [57,58], (ii) mass-transfer limitations dependent on the substrate’s polarity [59], and (iii) difficulty to improve percentage conversion by biocatalyst concentration as seen in this study. Ultimately, whole-cell biocatalyst performance relies on the recombinant protein expression efficiency and therefore requires strong promoters and systematic genomic integration loci [60,61].

## 5. Conclusions

The recently identified monooxygenase CYP5035S7 of *P. arcularius* possesses considerable activity towards various PAHs and also formed a broader set of products than previously thought as showcased by the peaks at earlier elution time corresponding to dihydroxylated products or other more products. Products formed by CYP5035S7-catalysed biotransformation of common 2- or 3-cyclic PAH compounds revealed that the P450 preferred electron-rich molecules or activated positions for hydroxylation. For example, NPAHs were less tolerated due their inherent electron-deficient nature. However, for several medium-sized PAHs not always the expected, most electron-rich positions were targeted. Instead, the regioselective outcome had often been influenced by the substrate’s orientation due to the sterically restricted active site. This combination of steric factors and the substrate’s electron density gives insight to its active-site geometry.

This paper aims at merging the research fields of white biotechnology and synthetic chemistry via enzymatic late-stage functionalisation: Applying the knowledge of fungal P450 bioremediation ability [62] enables the exploration of its potential in PAH derivatisation for synthetic purposes.

## Data Availability

The data presented in this study are openly available for all figures and samples.

## Supplementary Materials

The following are available online at https://www.mdpi.com/article/10.3390/biom11111708/s1, Figures S1, S3, S6, S9 and S12: HPLC conversion profiles of phenanthrene, azulene, acenpahthene, fluorene and anthracene, respectively, catalysed by CYP5035S7; Figures S2, S4, S7, S10 and S13: Differences in HPLC conversion profiles of phenanthrene, azulene, acenpahthene, fluorene and anthracene, respectively, at different OD; Figures S5, S8, S11 and S14: Preparative HPLC profiles of azulene, acenpahthene, fluorene and anthracene, respectively, for product isolation; Figure S15: Degree of aromaticity according to the *θ*′ index of four PAH structures.
