# Supplementary material for: Late-Stage Functionalisation of Polycyclic (N-Hetero-) Aromatic Hydrocarbons by Detoxifying CYP5035S7 Monooxygenase of the White-Rot Fungus Polyporus arcularius
Source: Biomolecules. 2021 Nov 17;11(11):1708. doi: 10.3390/biom11111708 (PMC8615681; doi:10.3390/biom11111708)
Supplement: Supplementary file 1 [file biomolecules-11-01708-s001.zip › biomolecules-1441785-supplementary.pdf]

Biomolecules

Supporting Information

for

Late-stage Functionalisation of Polycyclic (*N*-hetero-)Aromatic Hydrocarbons by Detoxifying CYP5035S7 Monooxygenase of the White-Rot Fungus *Polyporus Arcularius*

Nico D. Fessner,[1,*] Christopher Grimm,[2] Wolfgang Kroutil,[2,3] and Anton Glieder[1]

1 Institute of Molecular Biotechnology, Graz University of Technology, NAWI Graz, Graz, Austria
2 Institute of Chemistry, University of Graz, NAWI Graz, Graz, Austria
3 Field of Excellence BioHealth-University of Graz, Graz, Austria

* corresponding author: Nico D. Fessner
E-mail: nico.fessner11@alumni.imperial.ac.uk
phone: (+43)-316-873-4074
Fax: (+43)-316-873-9302

Phenanthrene

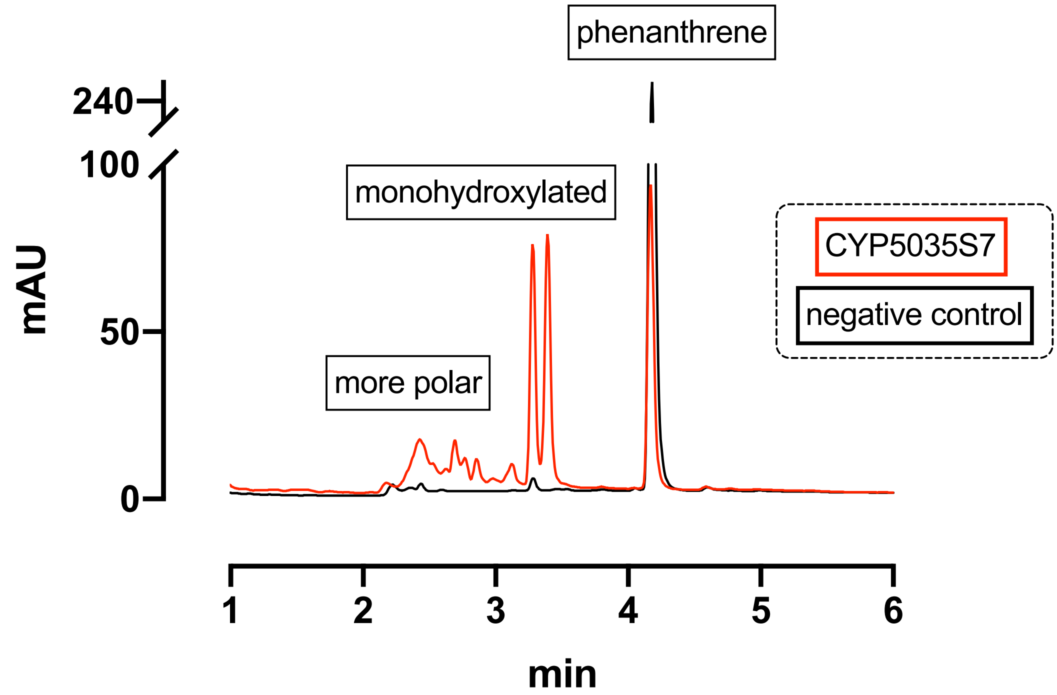

Figure S1: HPLC profile of phenanthrene conversion catalysed by CYP5035S7.

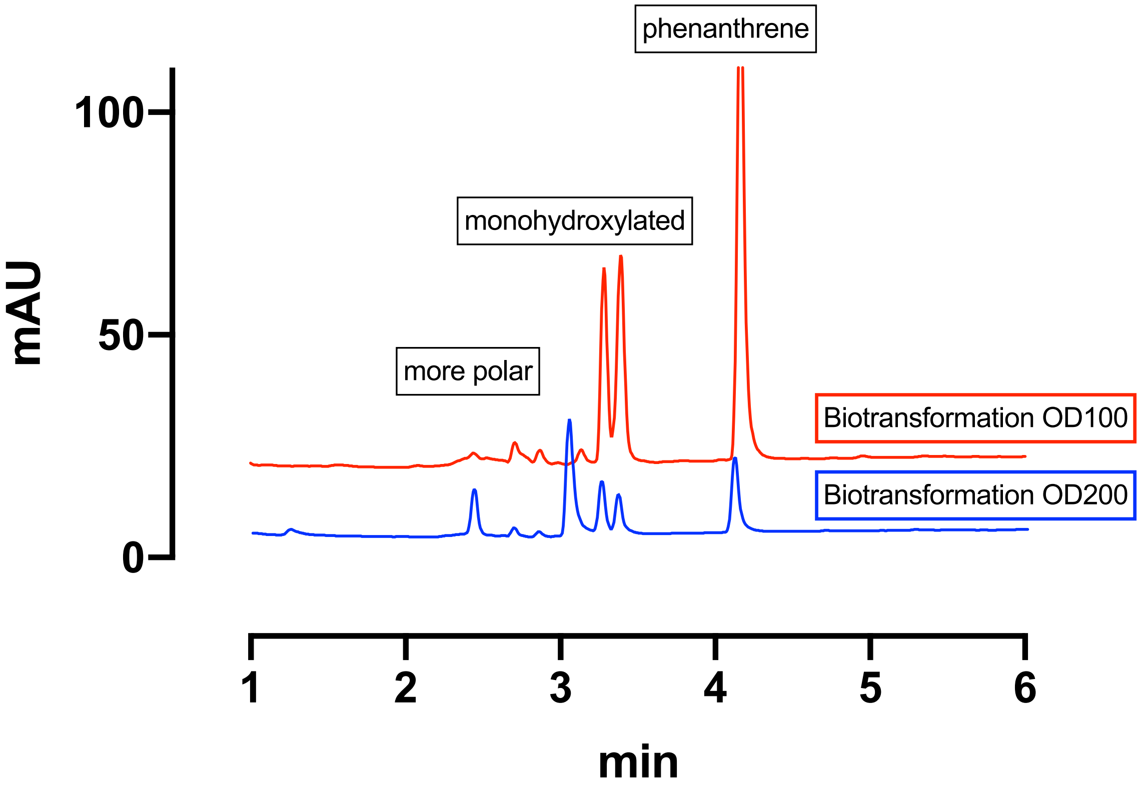

Figure S2: The difference between the HPLC profiles of the whole-cells biotransformation of phenanthrene catalysed by CYP5035S7 expressed in *P. pastoris* at different cell concentrations (OD).

Azulene

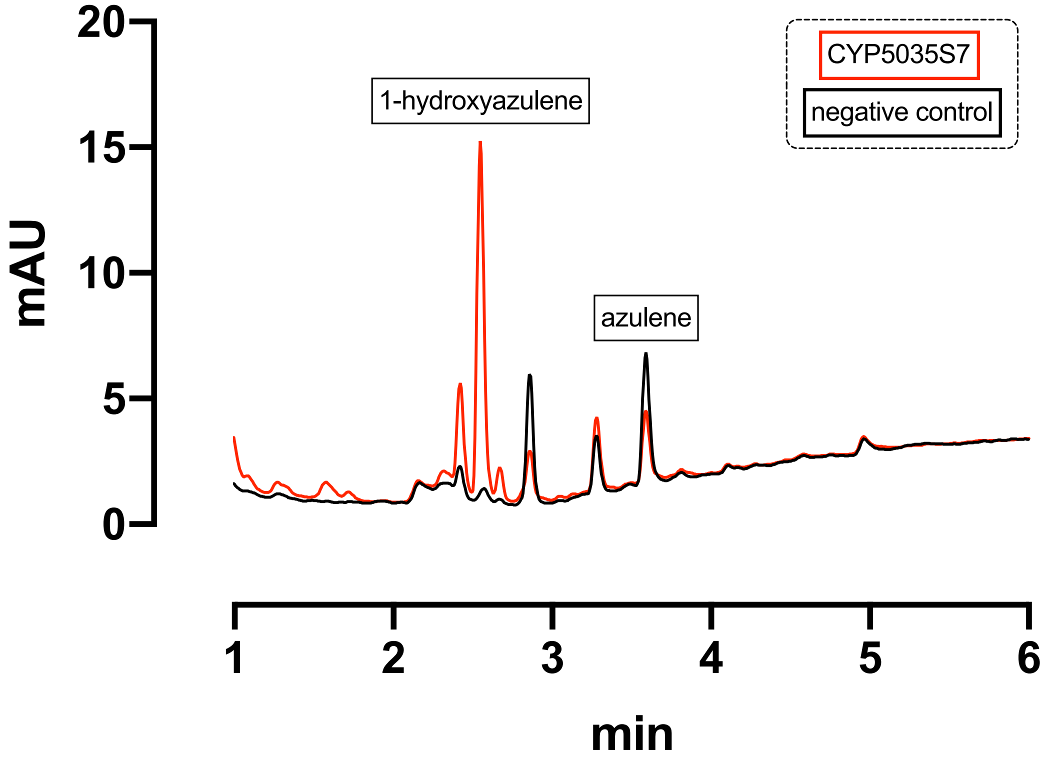

Figure S3: HPLC profile of azulene conversion catalysed by CYP5035S7.

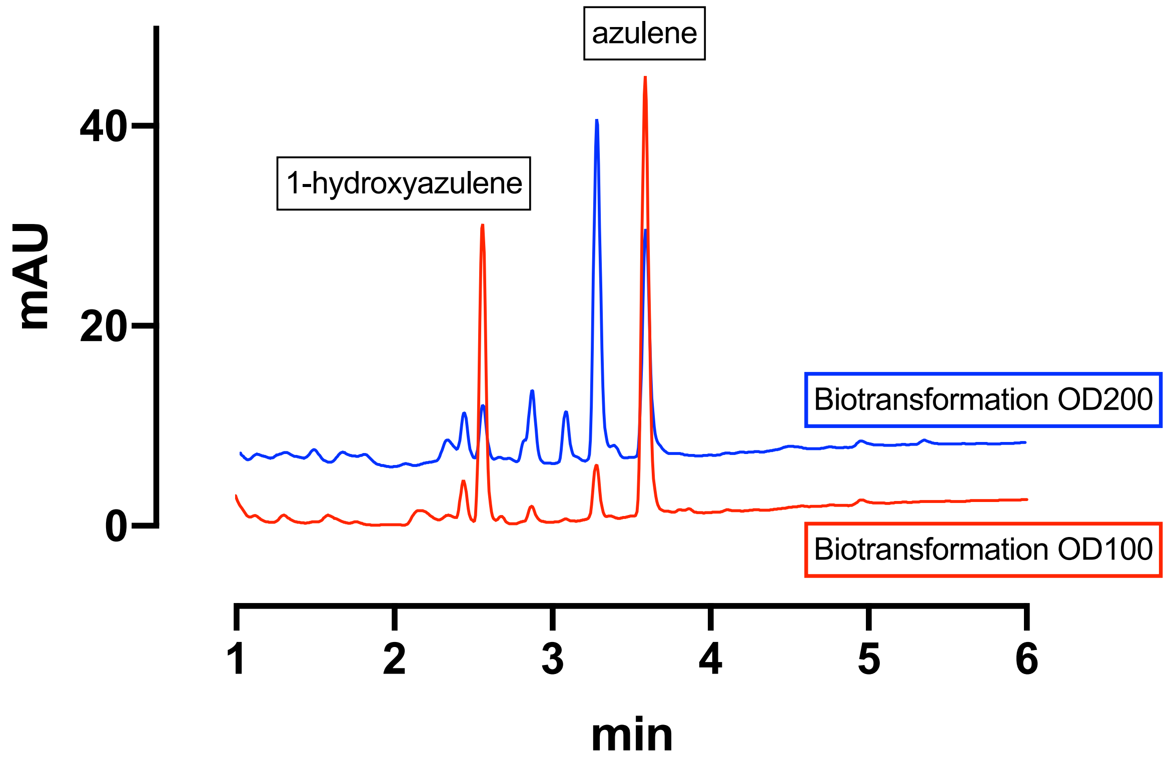

Azulene

Figure S4: The difference between the HPLC profiles of the whole-cells biotransformation of azulene catalysed by CYP5035S7 expressed in *P. pastoris* at different cell concentrations (OD).

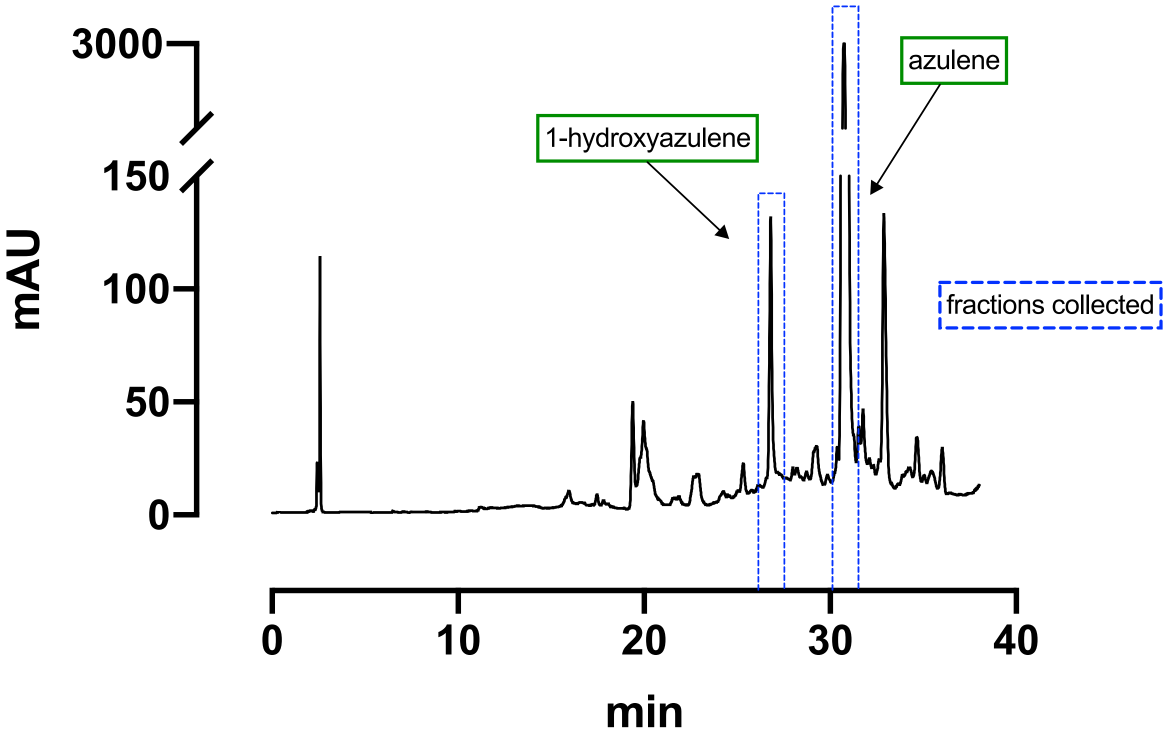

Figure S5: Preparative HPLC profile for product isolation of the scale-up azulene whole-cell biotransformation employing CYP5035S7. The blue dashed line indicates the fractions collected and the arrows indicate the corresponding products framed in green that were identified by NMR analysis.

Acenapthene

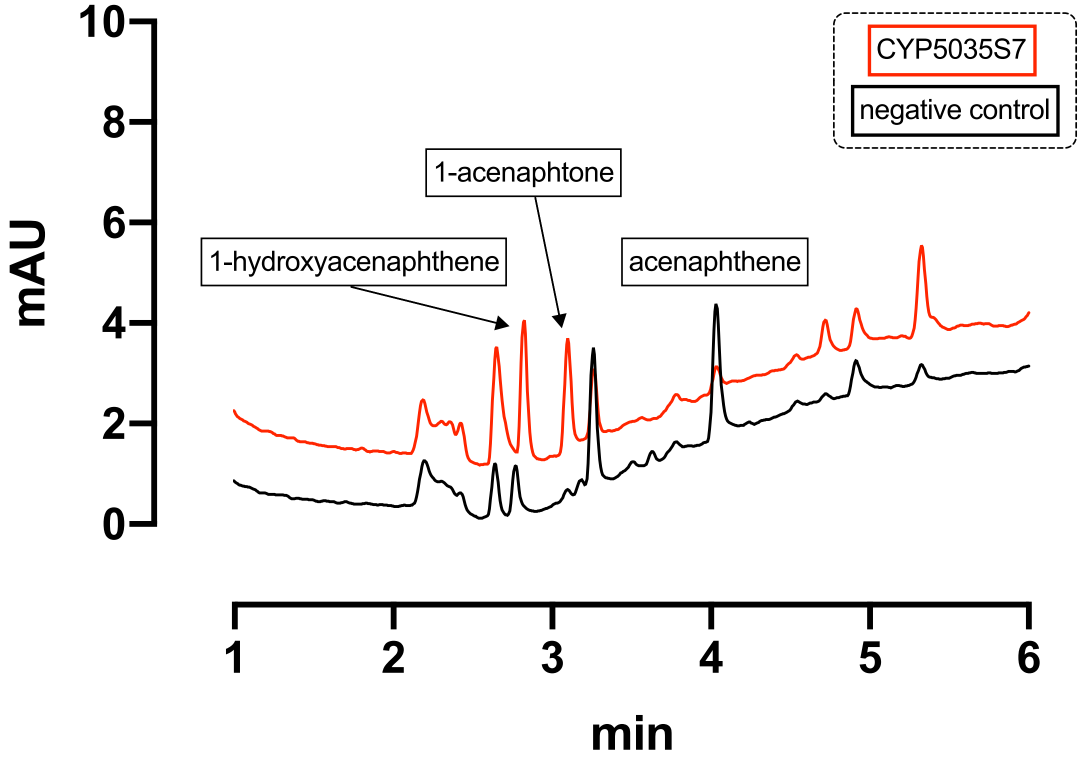

Figure S6: HPLC profile of acenaphthene conversion catalysed by CYP5035S7.

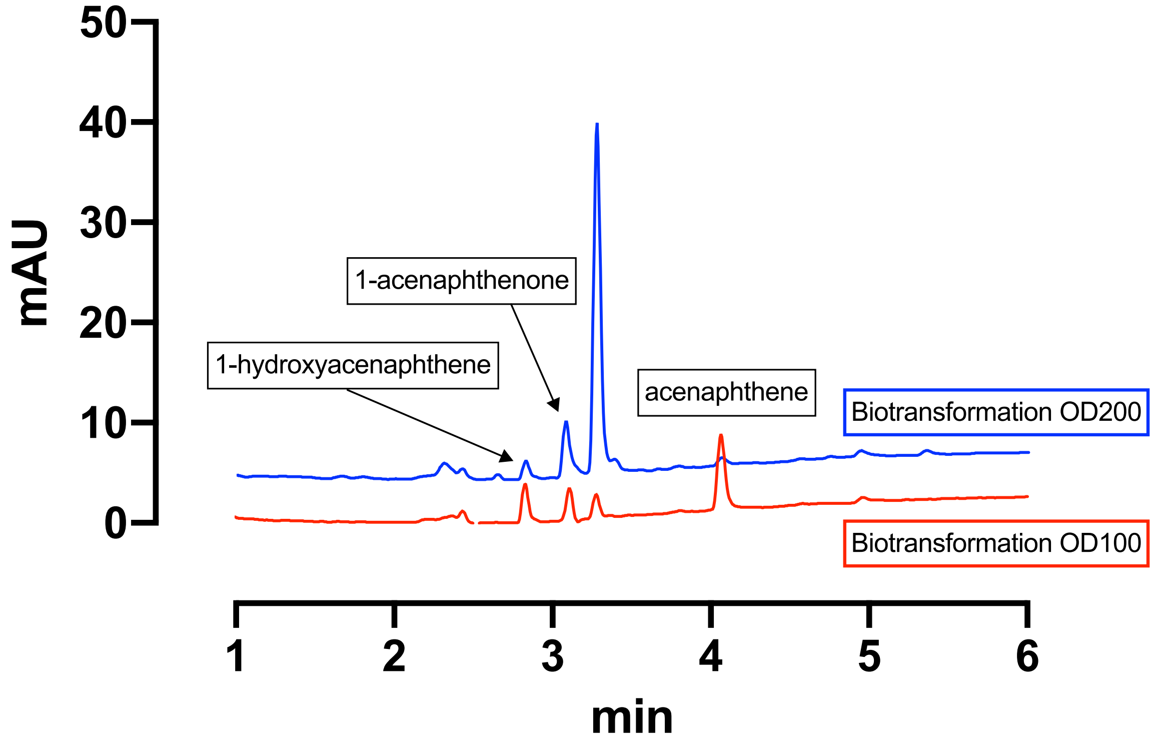

Figure S7: The difference between the HPLC profiles of the whole-cells biotransformation of acenaphthene catalysed by CYP5035S7 expressed in *P. pastoris* at different cell concentrations (OD).

Acenapthene

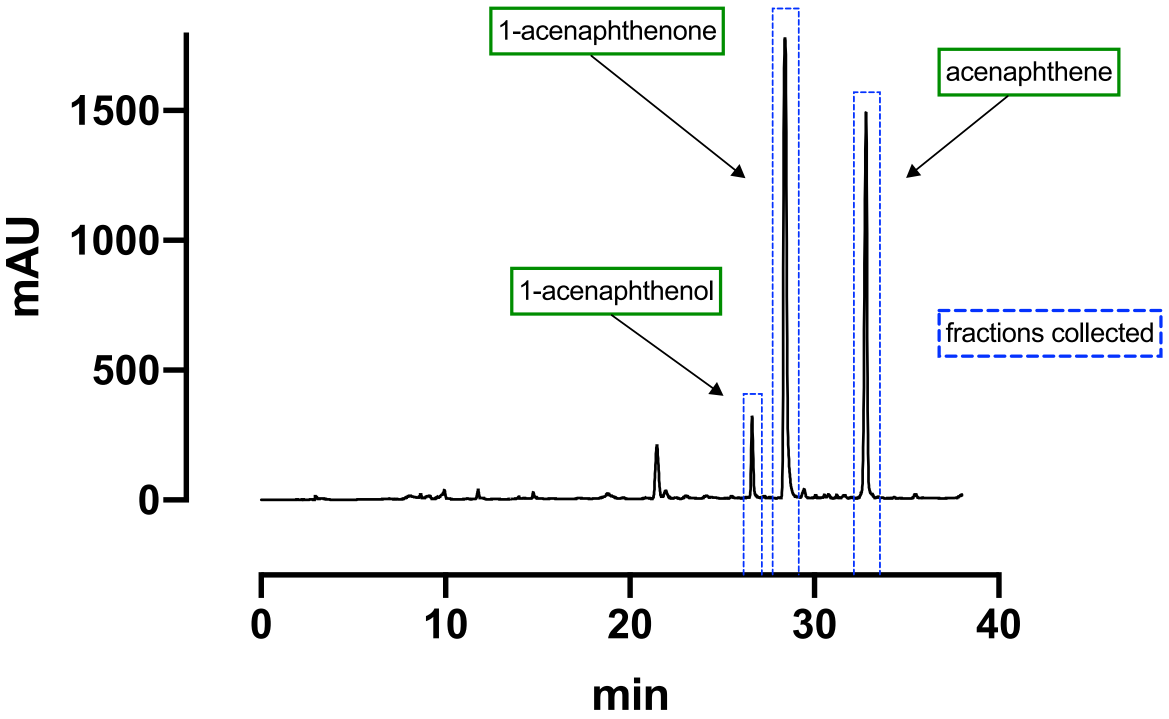

Figure S8: Preparative HPLC profile for product isolation of the scale-up anthracene whole-cell biotransformation employing CYP5035S7. The blue dashed line indicates the fractions collected and the arrows indicate the corresponding products framed in green that were identified by NMR analysis.

Fluorene

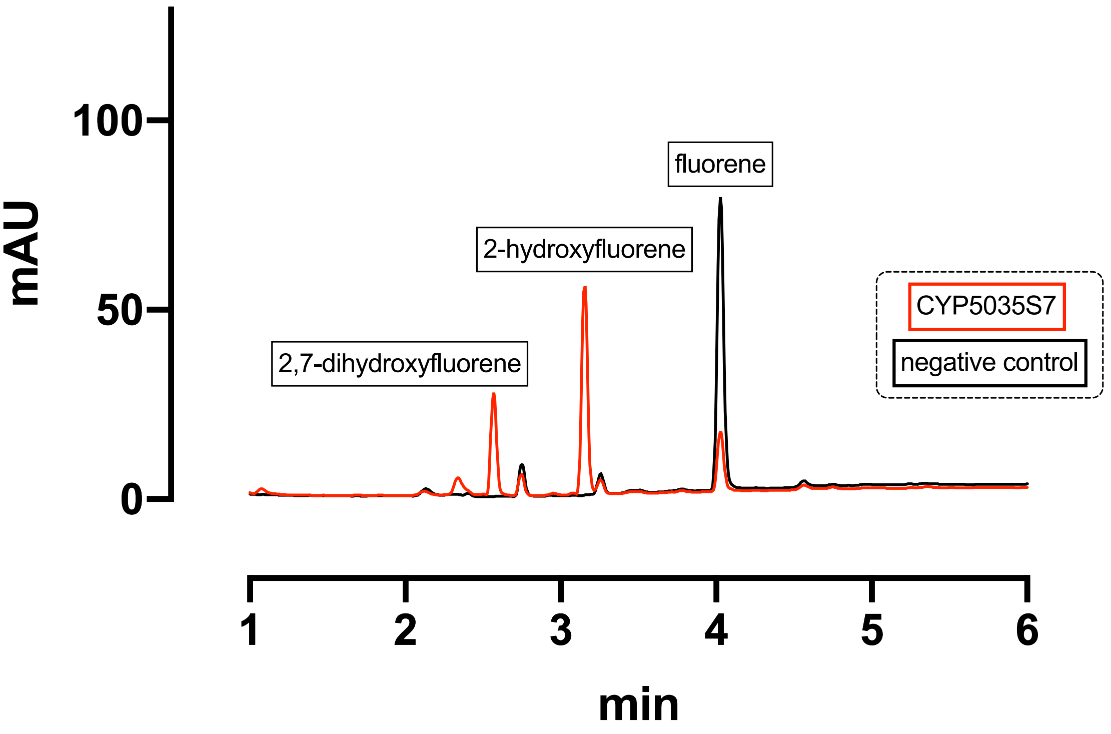

Figure S9: HPLC profile of fluorene conversion catalysed by CYP5035S7.

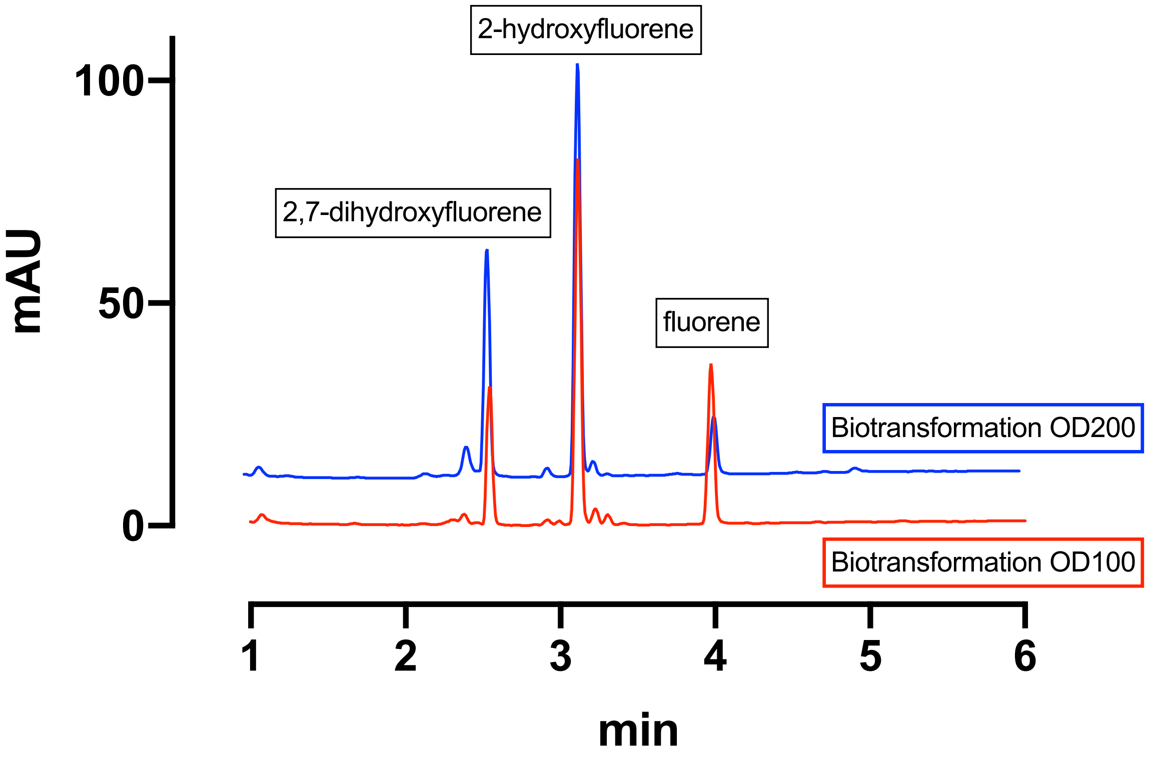

Figure S10: The difference between the HPLC profiles of the whole-cells biotransformation of fluorene catalysed by CYP5035S7 expressed in *P. pastoris* at different cell concentrations (OD).

Fluorene

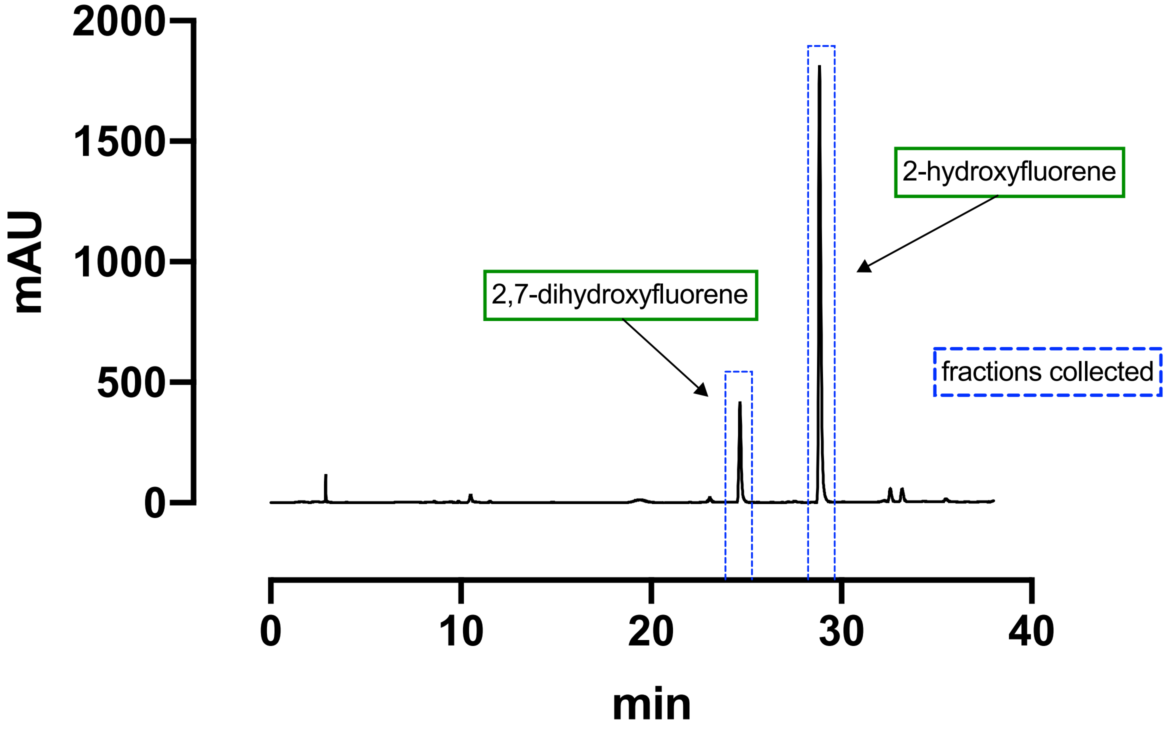

Figure S11: Preparative HPLC profile for product isolation of the scale-up fluorene whole-cell biotransformation employing CYP5035S7. The blue dashed line indicates the fractions collected and the arrows indicate the corresponding products framed in green that were identified by NMR analysis.

Anthracene

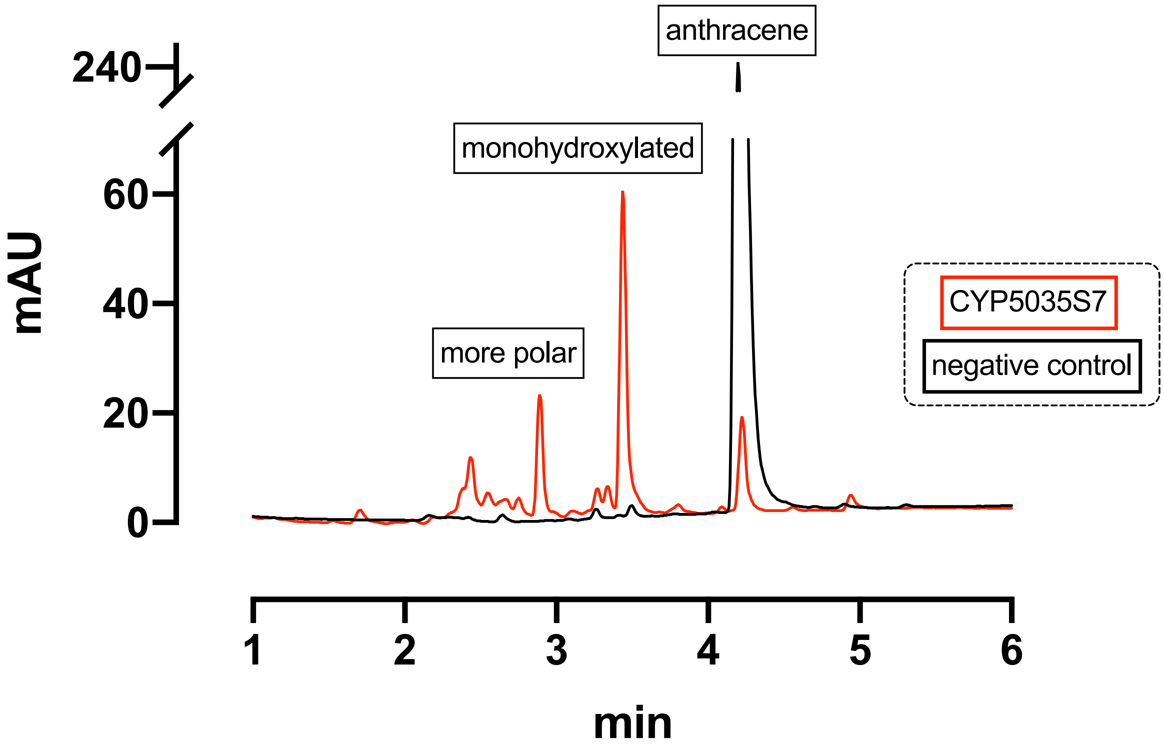

Figure S12: HPLC profile of anthracene conversion catalysed by CYP5035S7.

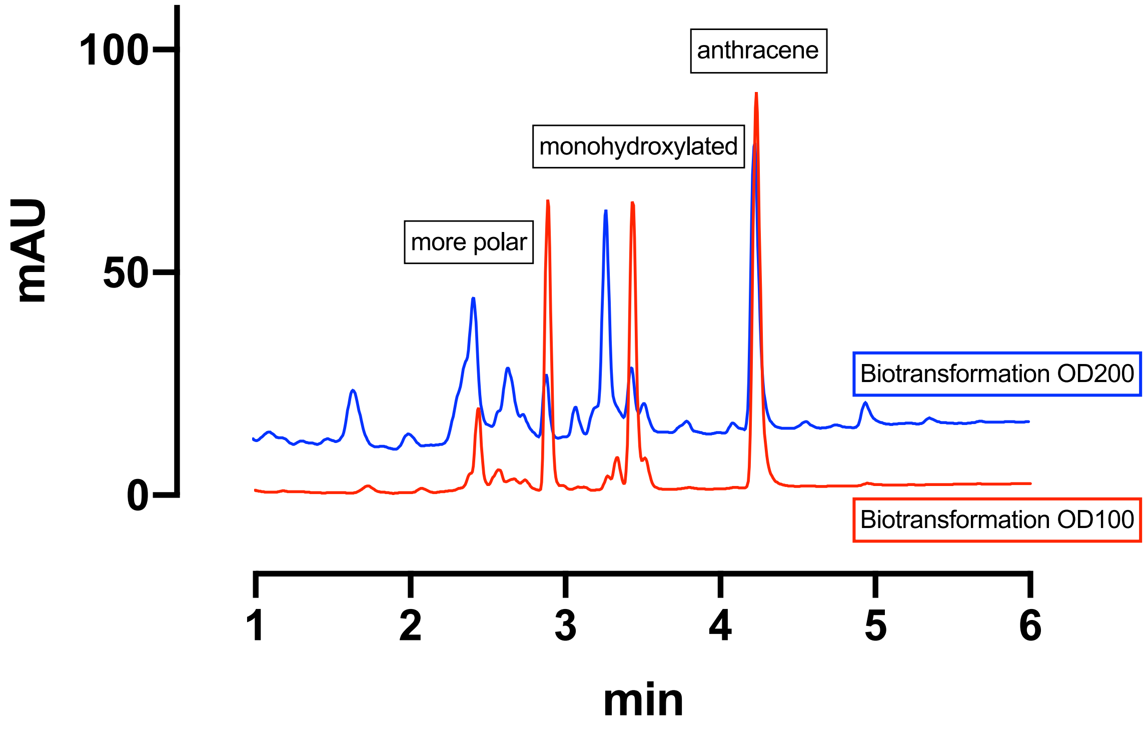

Figure S13: The difference between the HPLC profiles of the whole-cells biotransformation of anthracene catalysed by CYP5035S7 expressed in *P. pastoris* at different cell concentrations (OD).

Anthracene

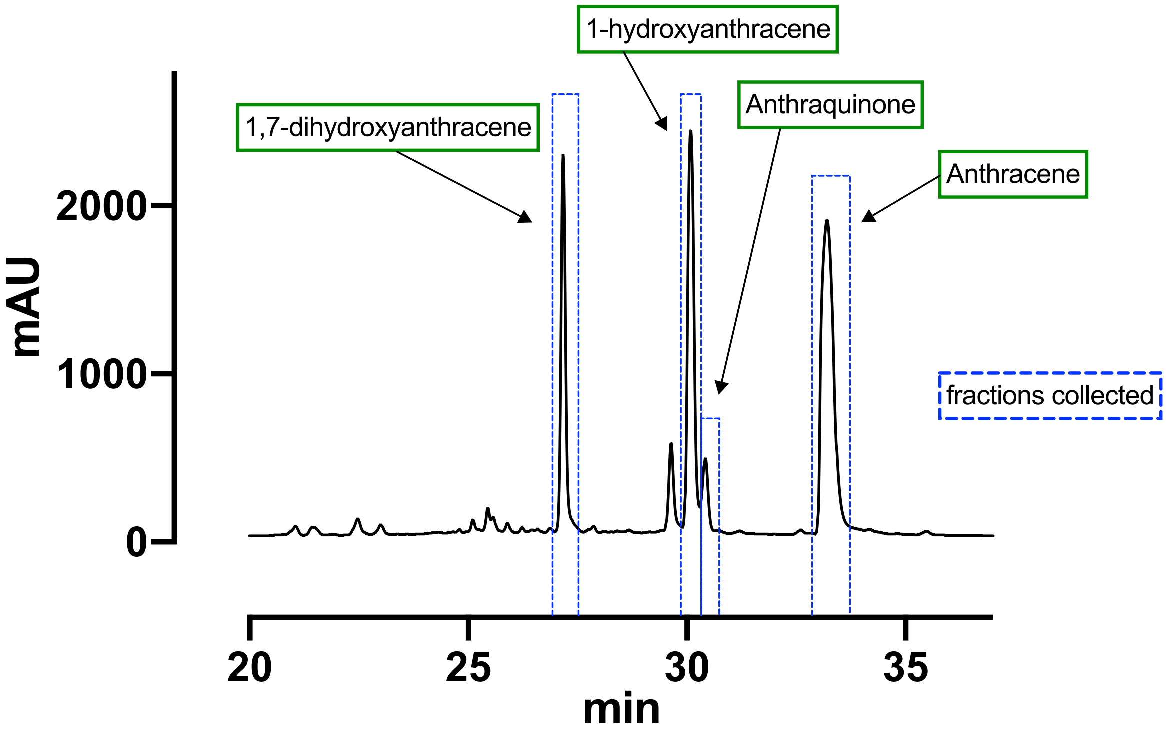

Figure S14: Preparative HPLC profile for product isolation of the scale-up anthracene whole-cell biotransformation employing CYP5035S7. The blue dashed line indicates the fractions collected and the arrows indicate the corresponding products framed in green that were identified by NMR analysis.

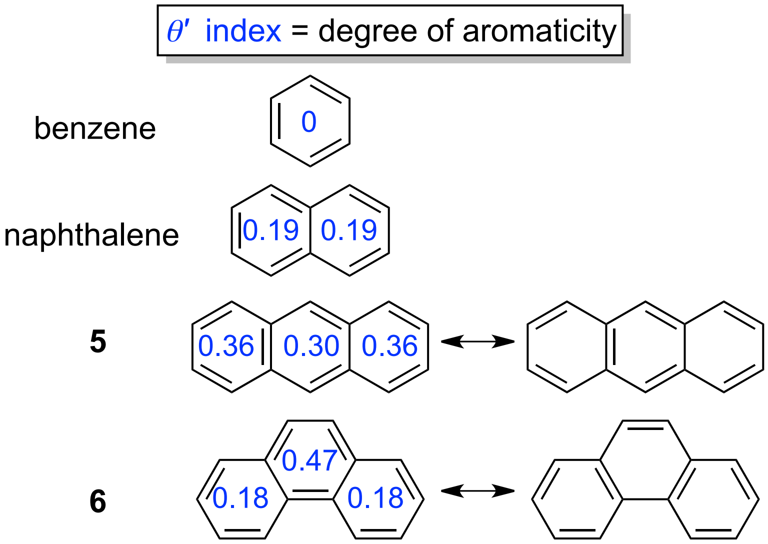

Figure S15: The degree of aromaticity according to the θ' index[1] along with resonance structures of Clar's aromatic π-sextet approach of four PAH structures are shown.

References

[1] M. Estévez-Fregoso, J. Hernández-Trujillo, *Phys. Chem. Chem. Phys.* **2016**, *18*, 11792–11799.

NMR spectra

PAH numbering of the compounds converted by CYP5035S7 at semi-preparative-scale in this study:

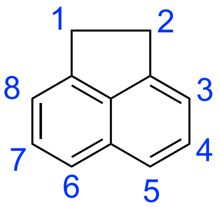

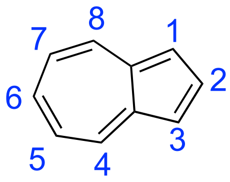

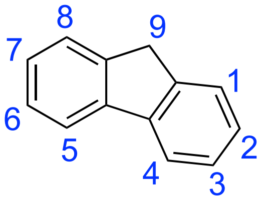

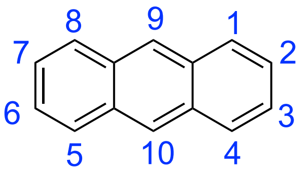

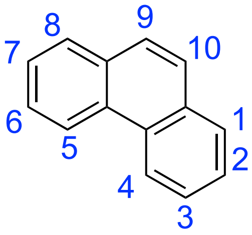

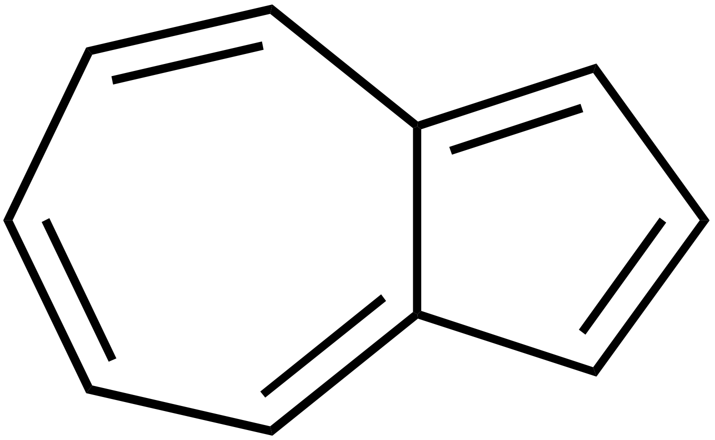

2

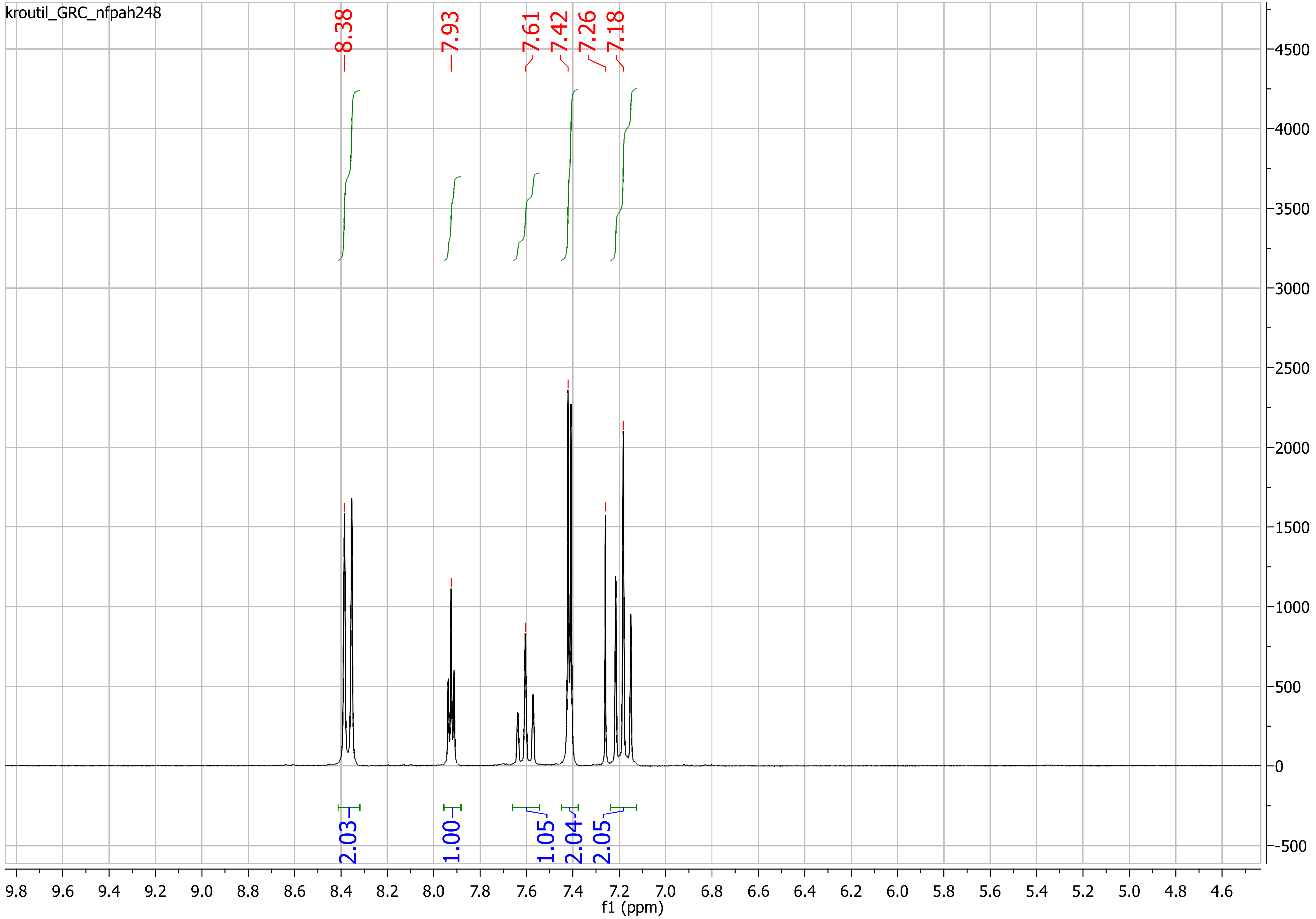

kroutil_GRC_nfpah248
8.38
7.93
7.61
7.42
7.26
7.18
2.03
1.00
1.05
2.04
2.05
f1 (ppm)

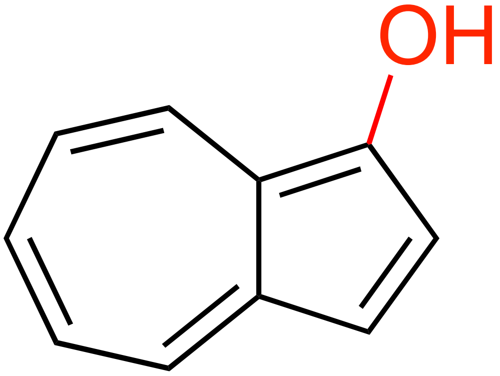

1-hydroxyazulene (**11**)

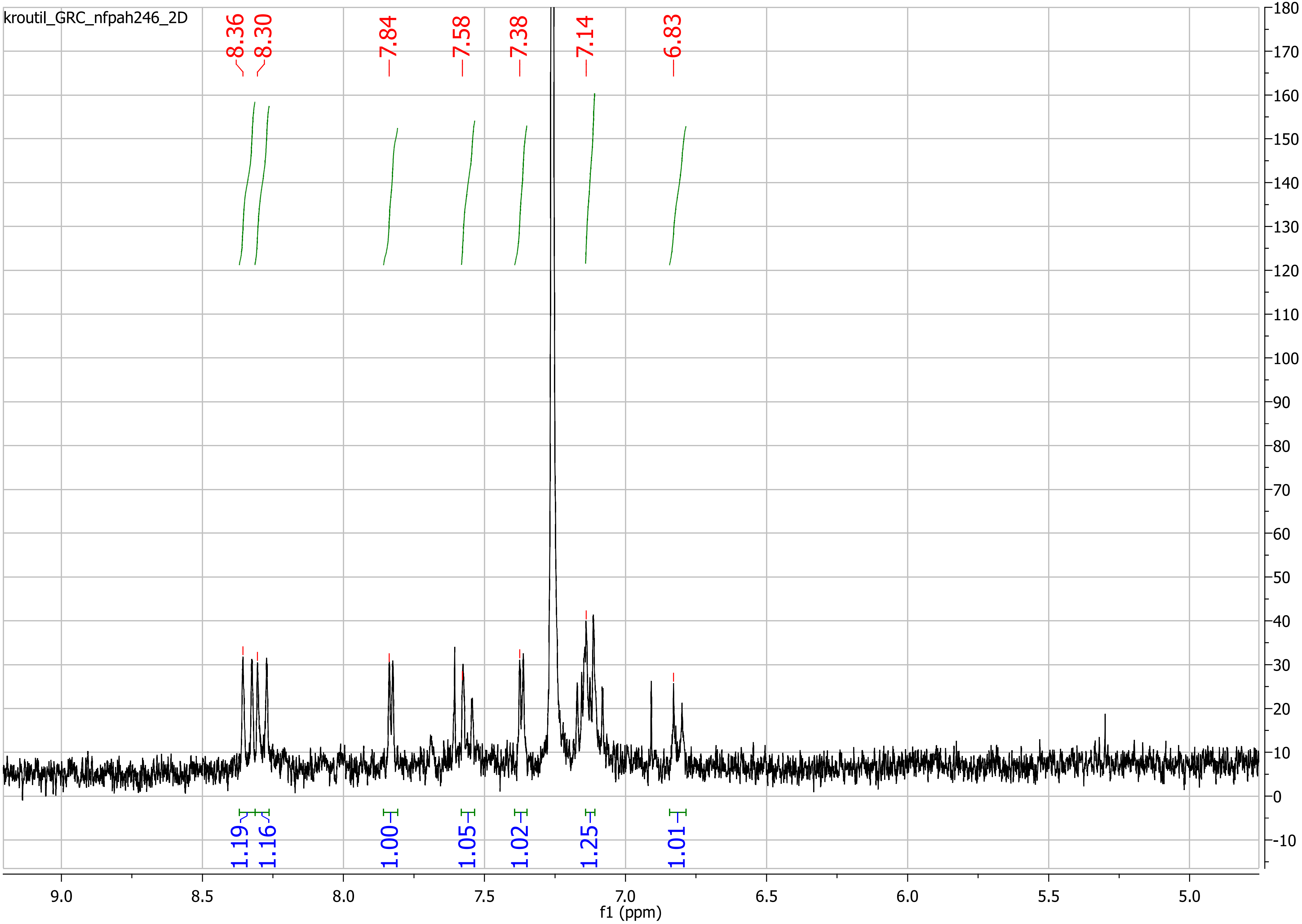

kroutil_GRC_nfpah246_2D
8.36
8.30
7.84
7.58
7.38
7.14
6.83
1.19
1.16
1.00
1.05
1.02
1.25
1.01
f1 (ppm)

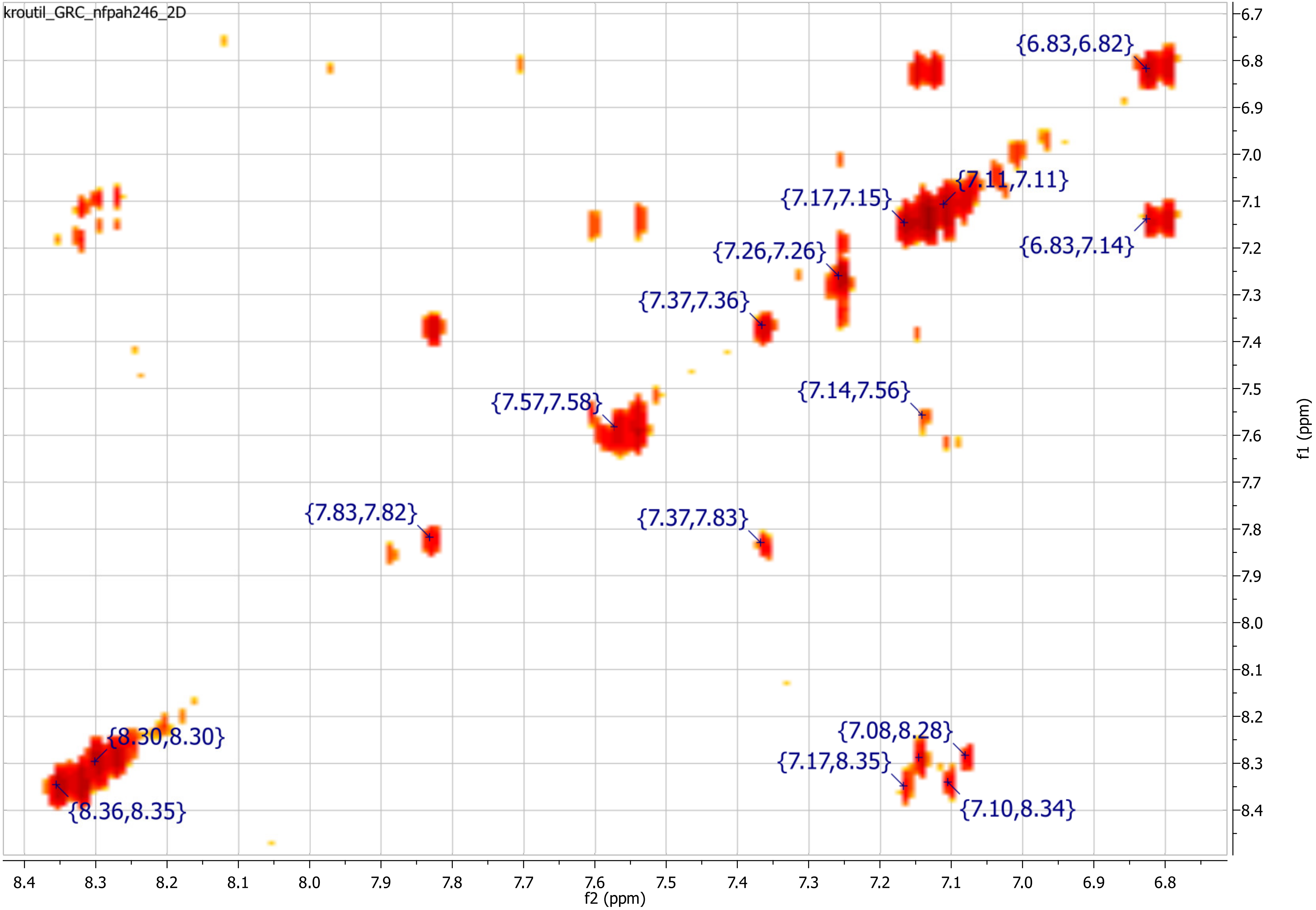
kroutil_GRC_nfpah246_2D
{6.83,6.82}
{7.11,7.11}
{7.17,7.15}
{6.83,7.14}
{7.26,7.26}
{7.37,7.36}
{7.14,7.56}
{7.57,7.58}
{7.83,7.82}
{7.37,7.83}
{8.30,8.30}
{8.36,8.35}
{7.08,8.28}
{7.17,8.35}
{7.10,8.34}
f2 (ppm)
f1 (ppm)

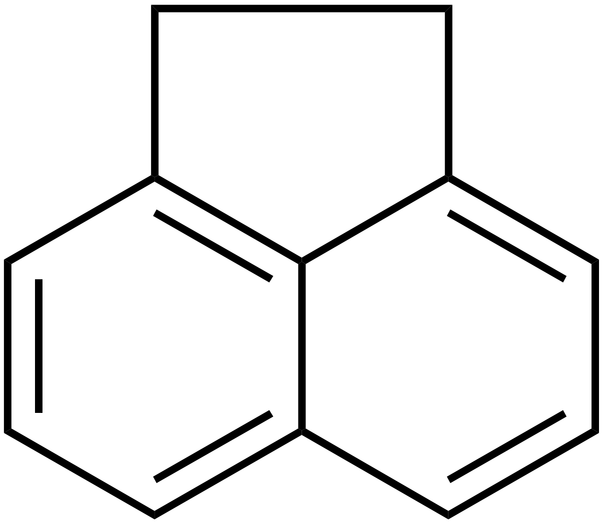

3

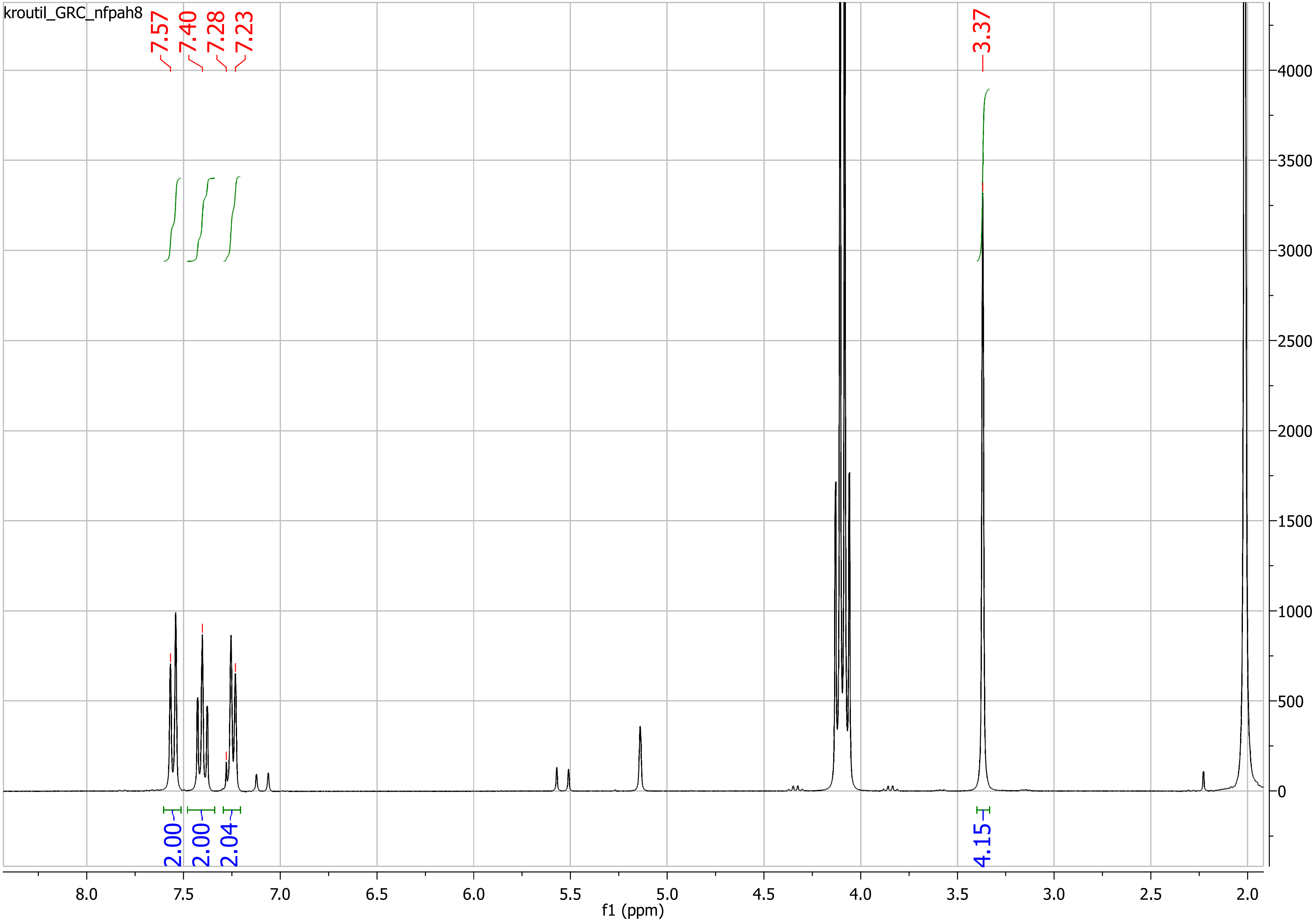
kroutil_GRC_nfpah8
7.57
7.40
7.28
7.23
3.37
2.00
2.00
2.04
4.15
f1 (ppm)

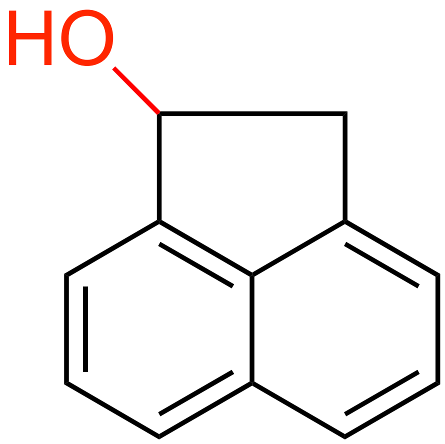

1-hydroxyacenaphthene (**12**)

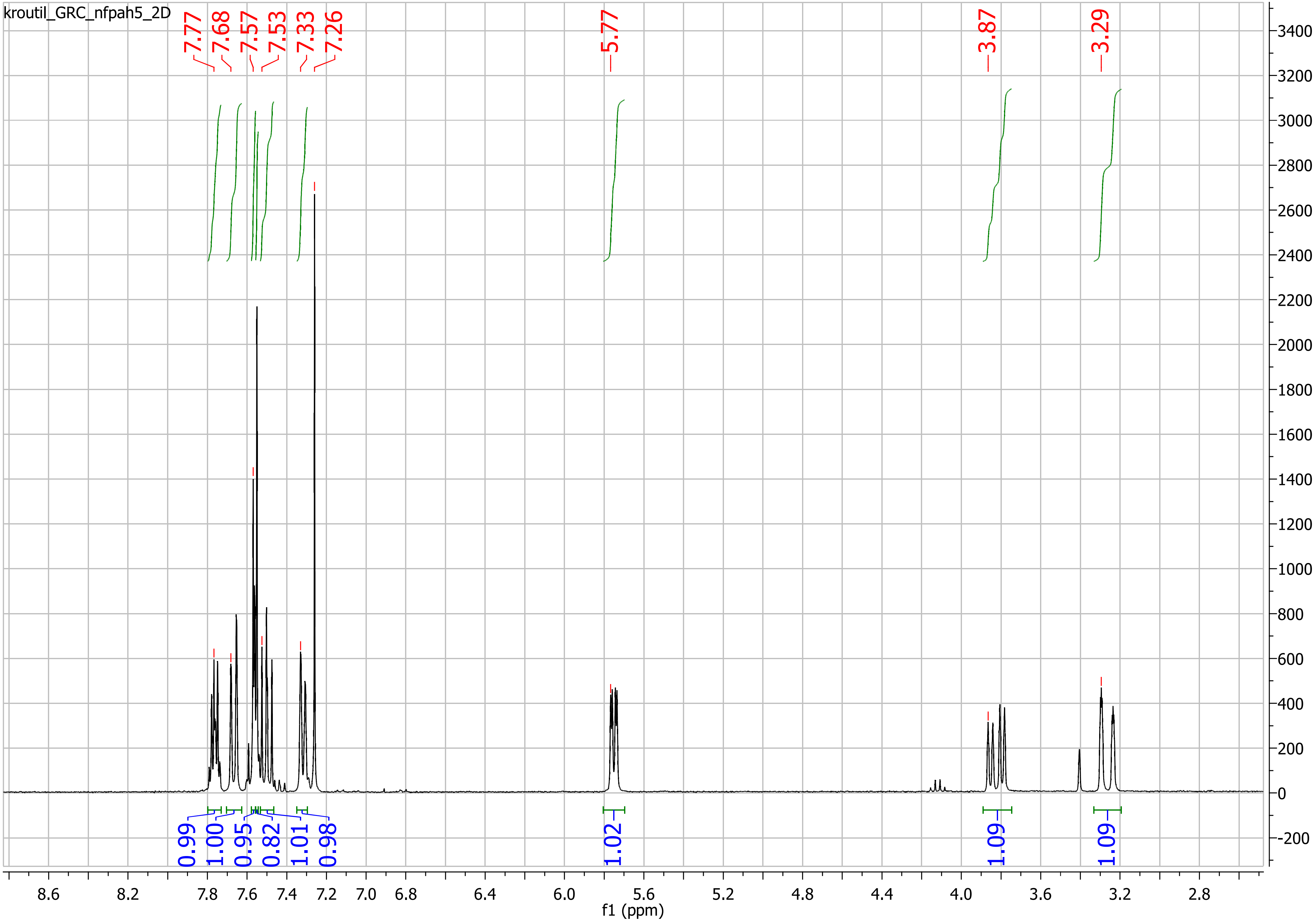
kroutil_GRC_nfpah5_2D
7.77
7.68
7.57
7.53
7.33
7.26
5.77
3.87
3.29
0.99
1.00
0.95
0.82
1.01
0.98
1.02
1.09
1.09
f1 (ppm)

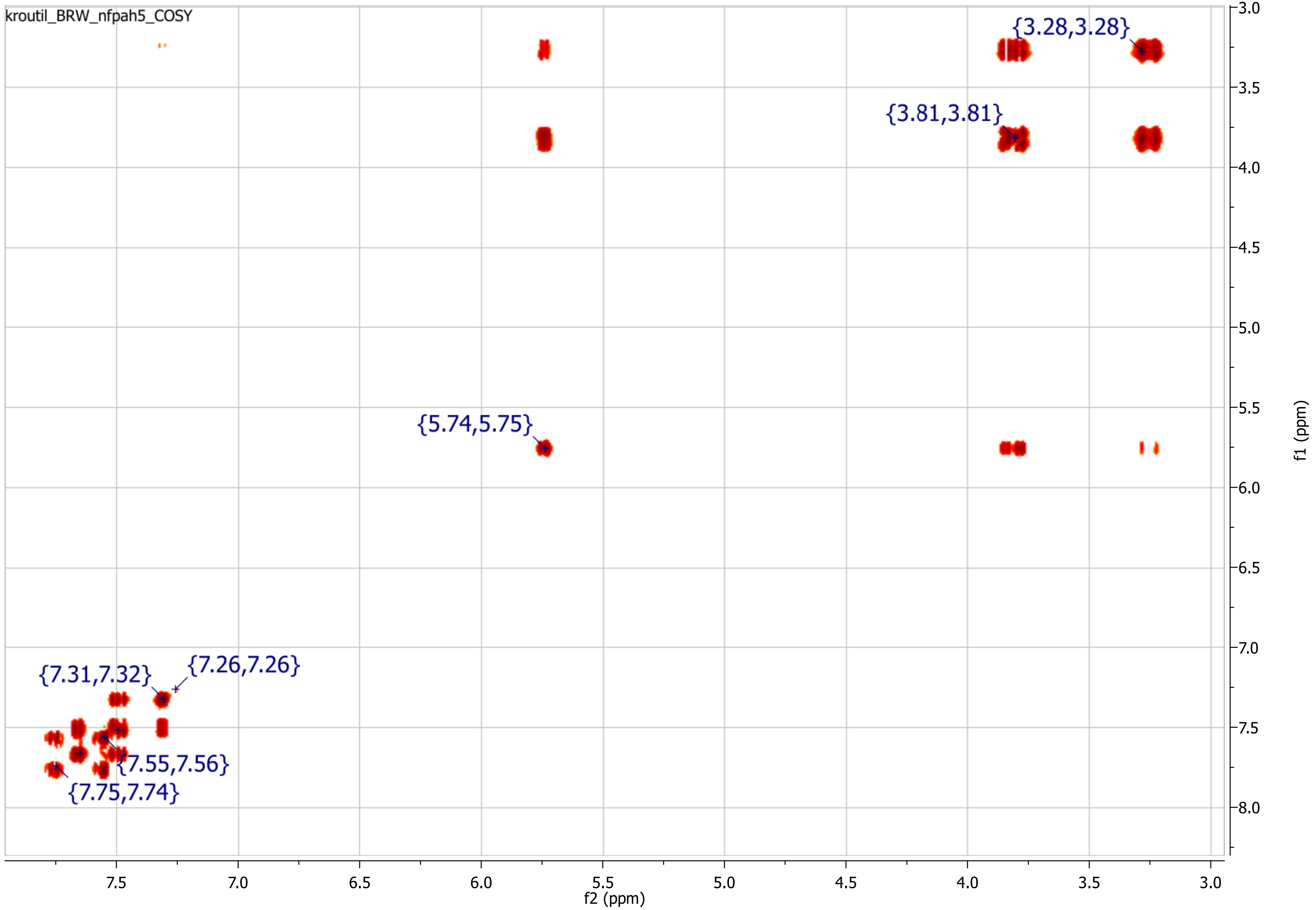

kroutil_BRW_nfpah5_COSY
{3.28,3.28}
{3.81,3.81}
{5.74,5.75}
{7.31,7.32}
{7.26,7.26}
{7.55,7.56}
{7.75,7.74}
7.5
7.0
6.5
6.0
5.5
5.0
4.5
4.0
3.5
3.0
f2 (ppm)
3.0
3.5
4.0
4.5
5.0
5.5
6.0
6.5
7.0
7.5
8.0
f1 (ppm)

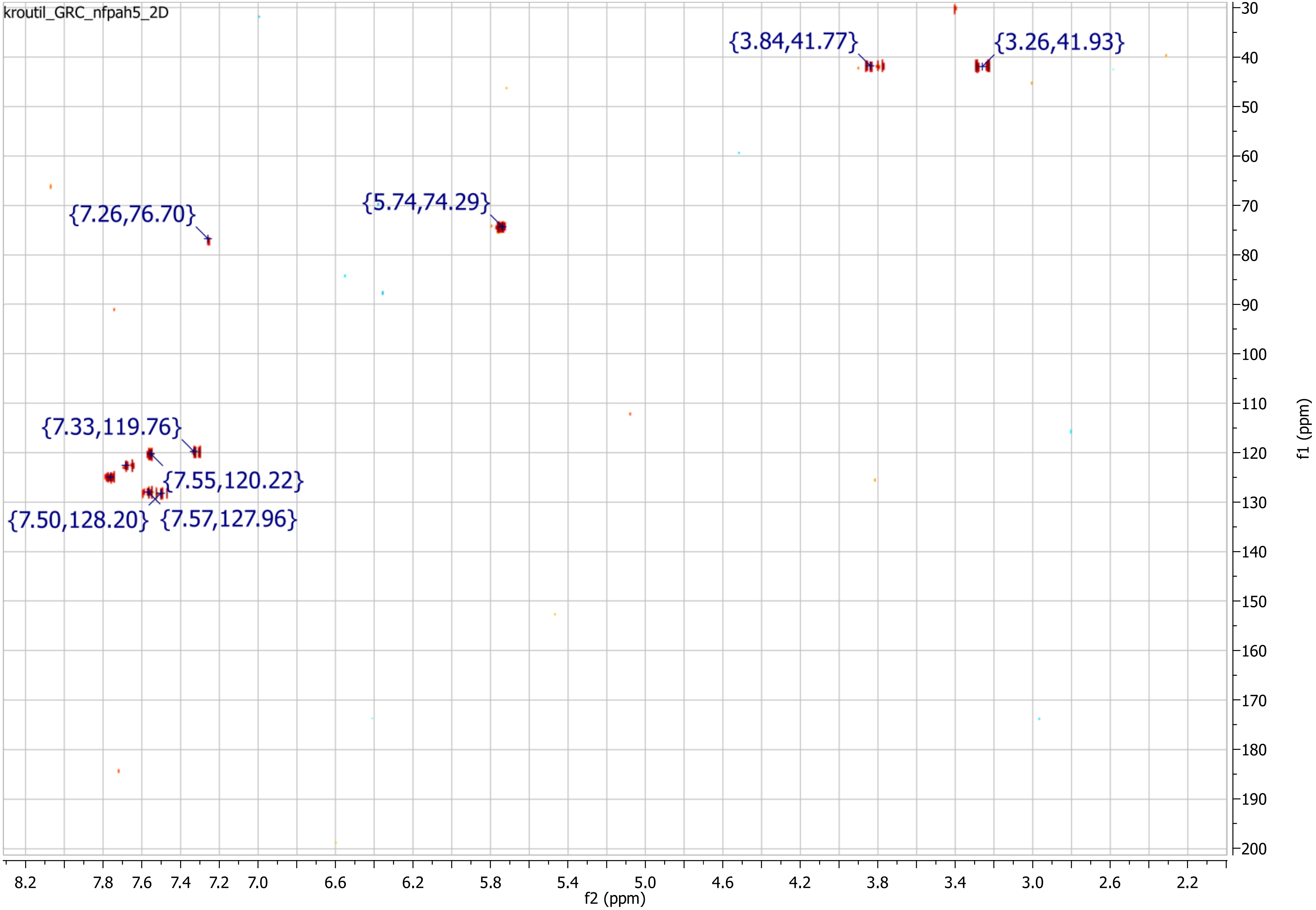

kroutil_GRC_nfpah5_2D
{3.84,41.77}
{3.26,41.93}
{7.26,76.70}
{5.74,74.29}
{7.33,119.76}
{7.55,120.22}
{7.50,128.20}
{7.57,127.96}
f2 (ppm)
f1 (ppm)

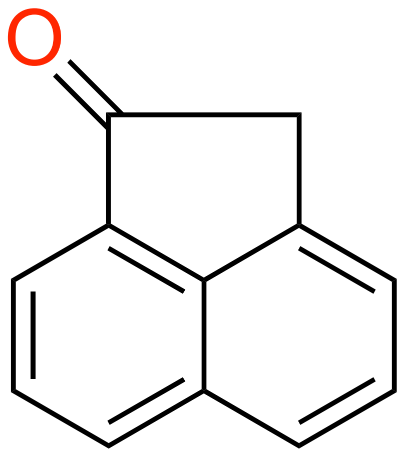

1-acenaphthenone (**13**)

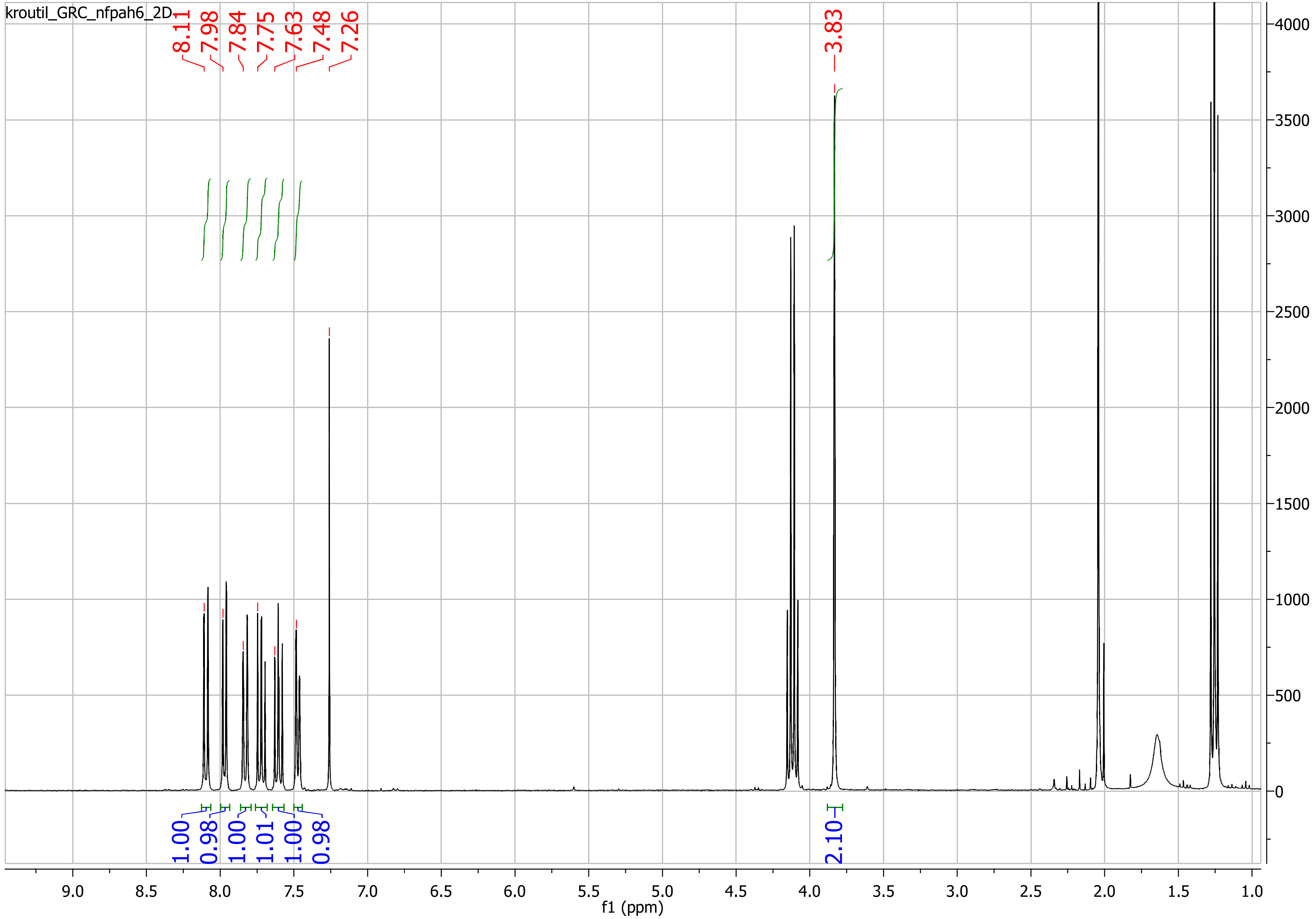
kroutil_GRC_nfpah6_2D
8.11
7.98
7.84
7.75
7.63
7.48
7.26
3.83
1.00
0.98
1.00
1.01
1.00
0.98
2.10
f1 (ppm)

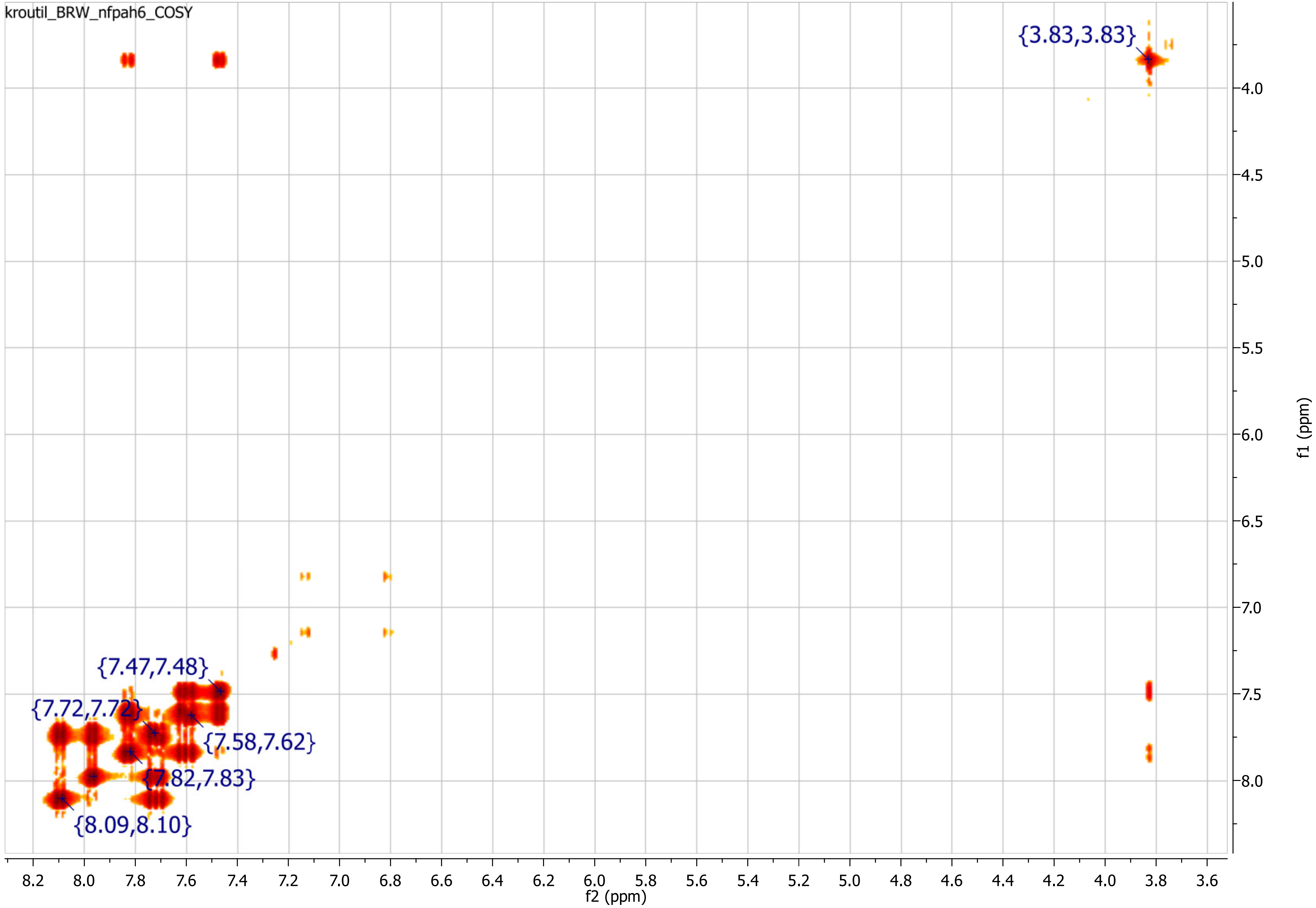
kroutil_BRW_nfpah6_COSY
{3.83,3.83}
{7.47,7.48}
{7.72,7.72}
{7.58,7.62}
{7.82,7.83}
{8.09,8.10}
f2 (ppm)
f1 (ppm)

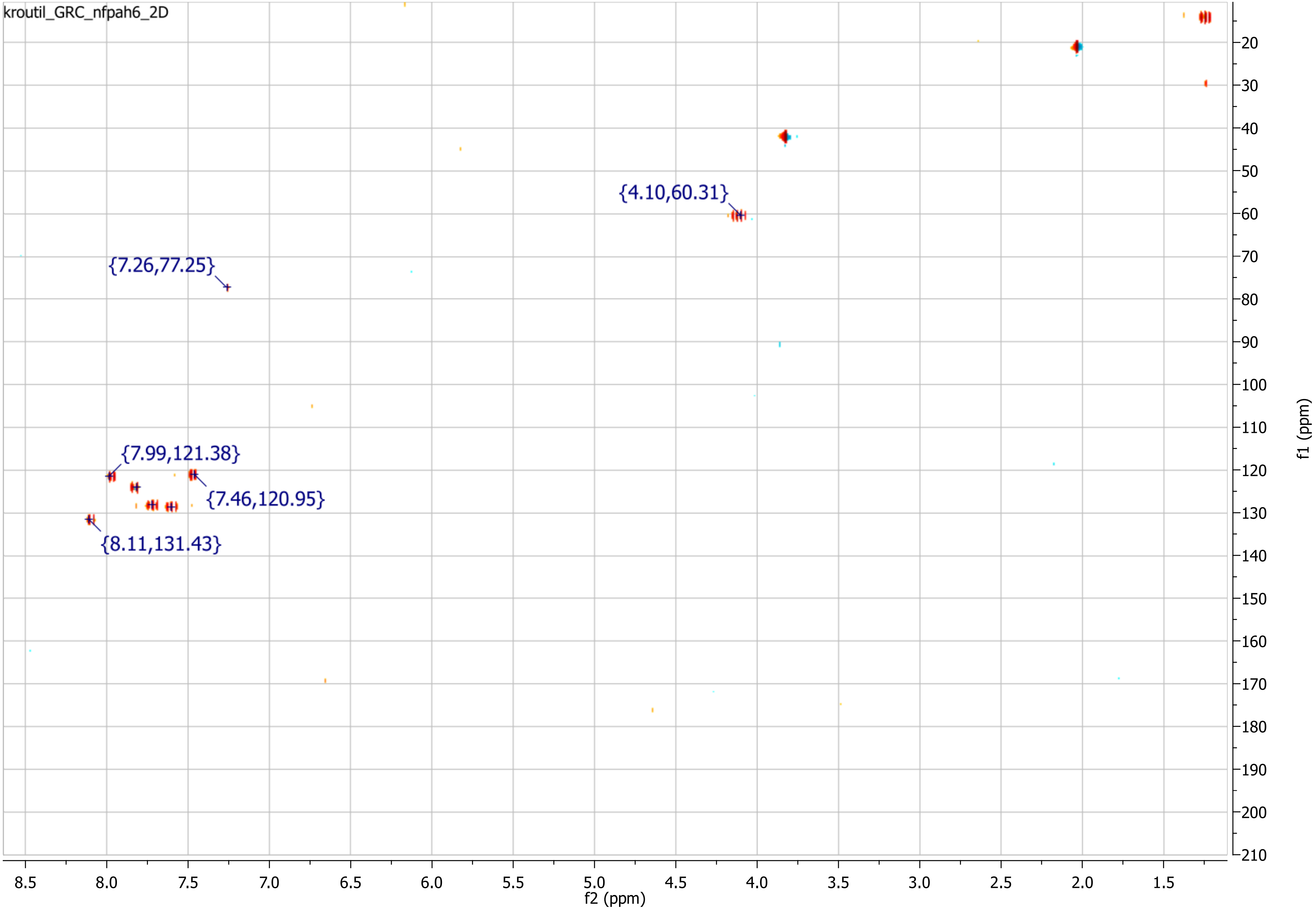

kroutil_GRC_nfpah6_2D
{4.10,60.31}
{7.26,77.25}
{7.99,121.38}
{7.46,120.95}
{8.11,131.43}
f2 (ppm)
f1 (ppm)

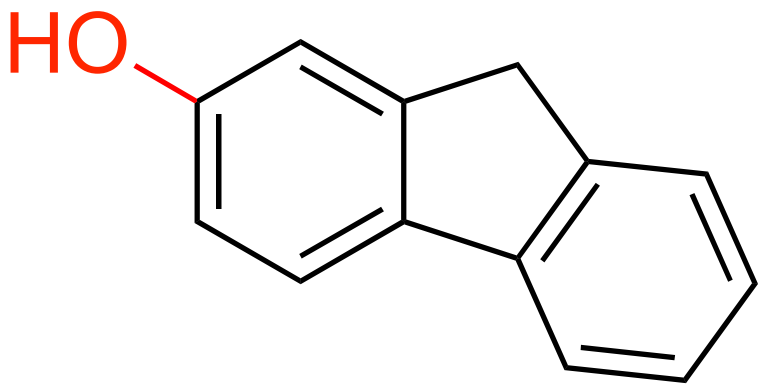

2-hydroxyfluorene (**14**)

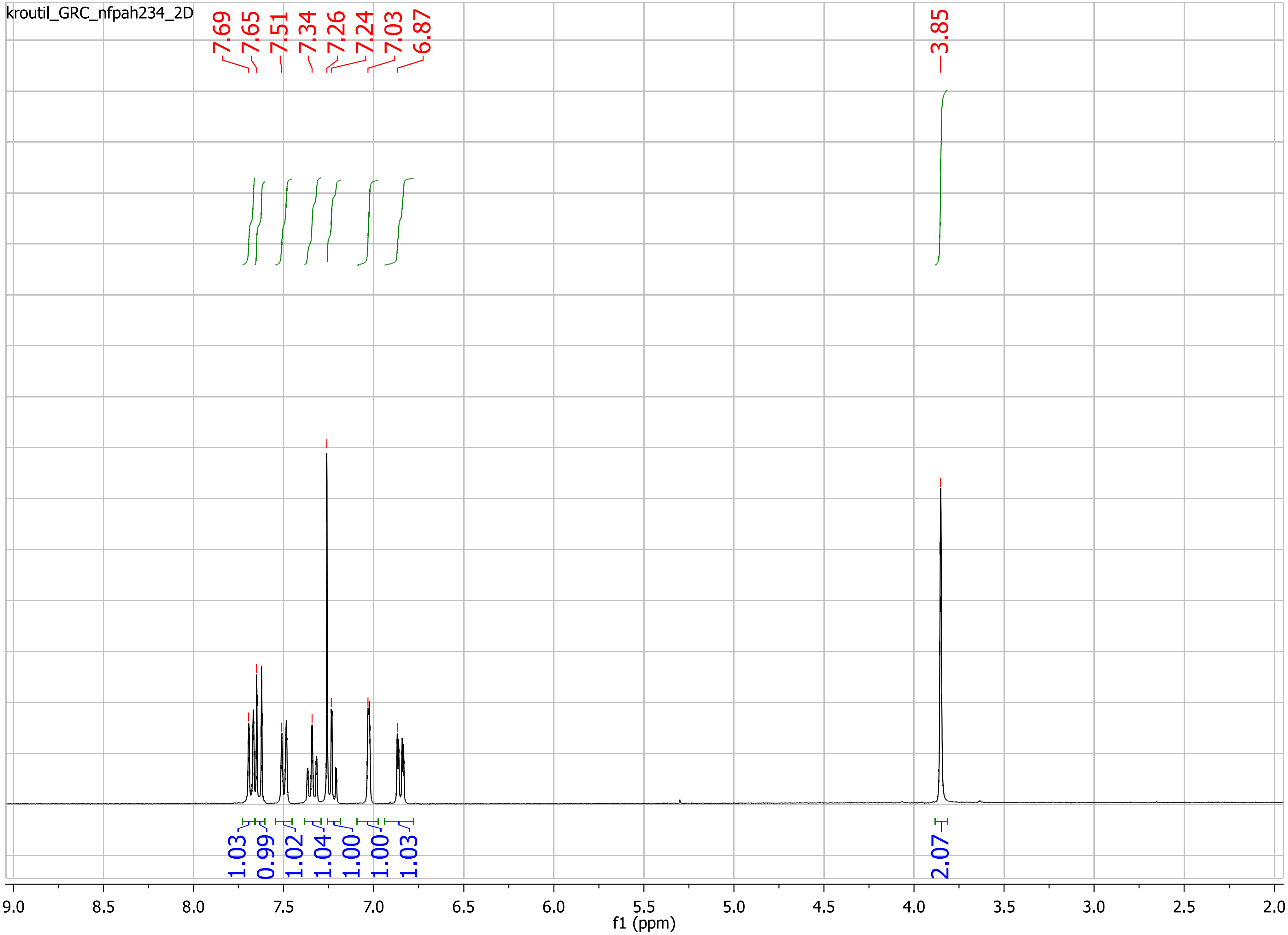
kroutil_GRC_nfpah234_2D
7.69
7.65
7.51
7.34
7.26
7.24
7.03
6.87
3.85
1.03
0.99
1.02
1.04
1.00
1.00
1.03
2.07
f1 (ppm)

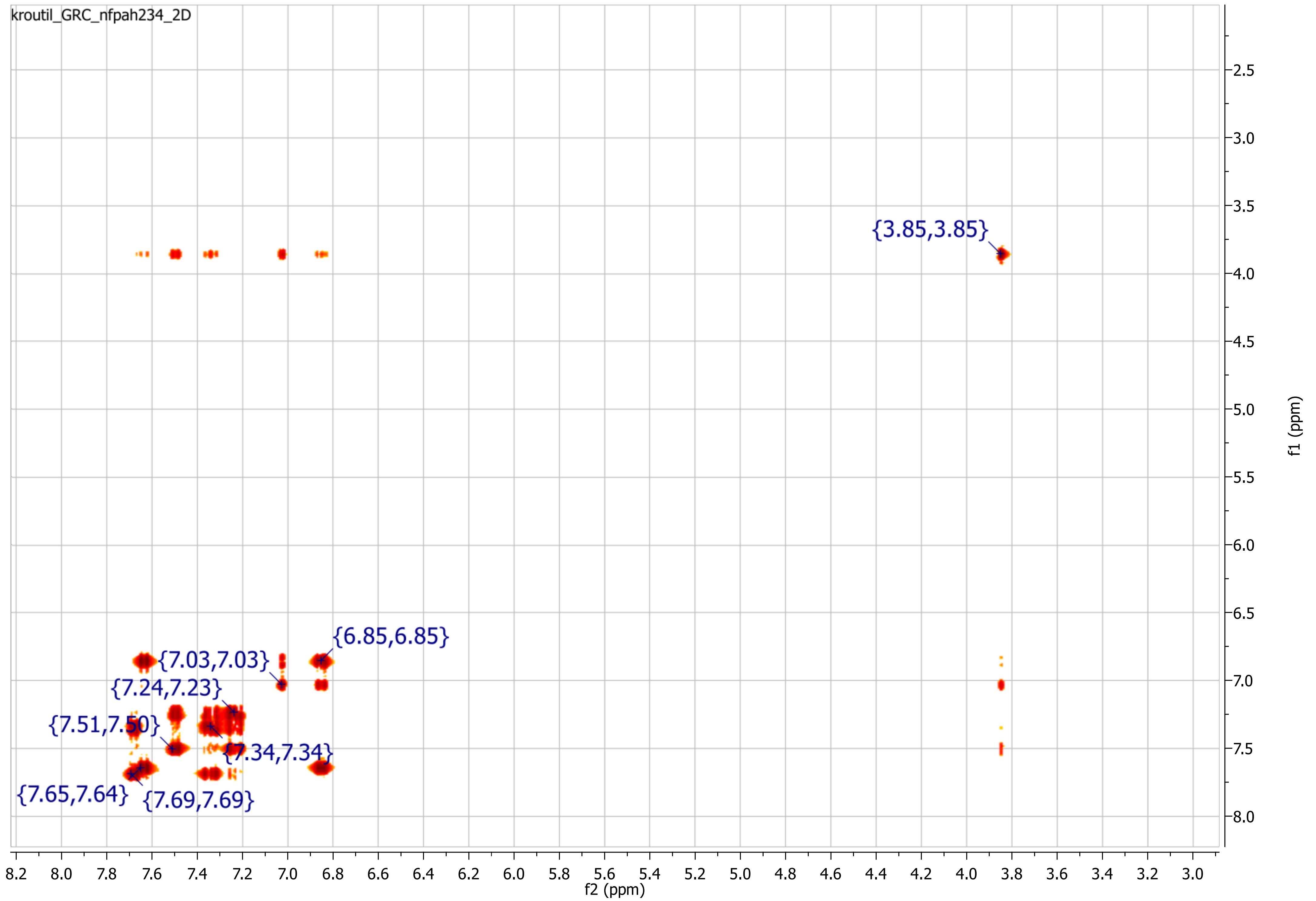
kroutil_GRC_nfpah234_2D
{3.85,3.85}
{6.85,6.85}
{7.03,7.03}
{7.24,7.23}
{7.51,7.50}
{7.34,7.34}
{7.65,7.64}
{7.69,7.69}
f2 (ppm)
f1 (ppm)

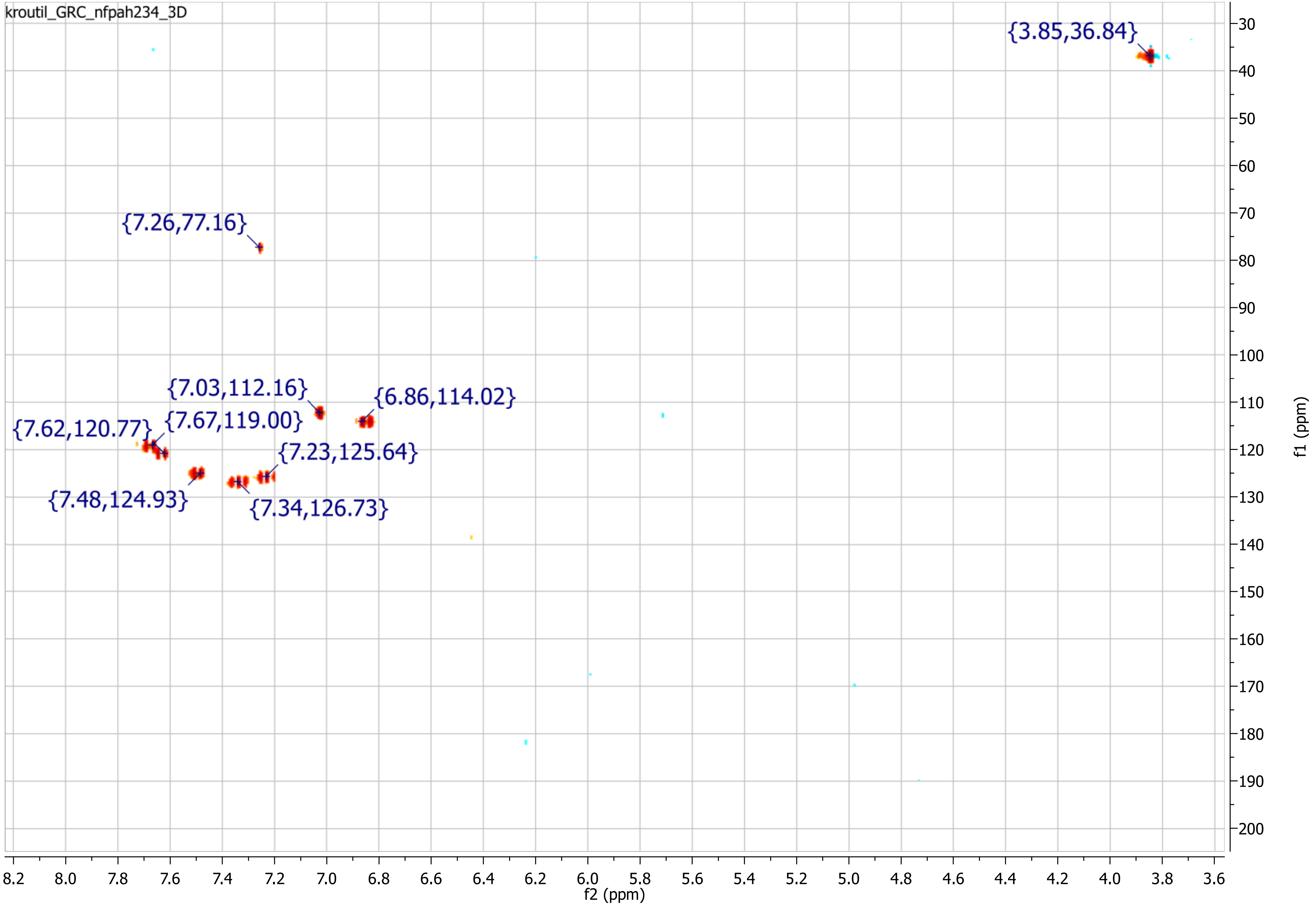
kroutil_GRC_nfpah234_3D
{3.85,36.84}
{7.26,77.16}
{7.03,112.16}
{6.86,114.02}
{7.62,120.77}
{7.67,119.00}
{7.23,125.64}
{7.48,124.93}
{7.34,126.73}
f2 (ppm)
f1 (ppm)

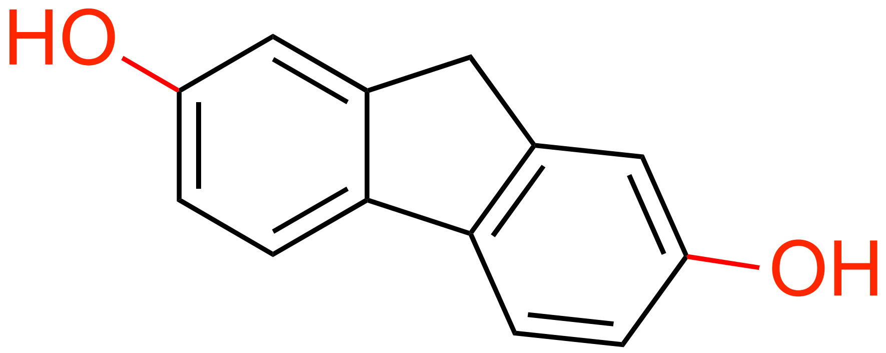

2,7-dihydroxyfluorene (**15**)

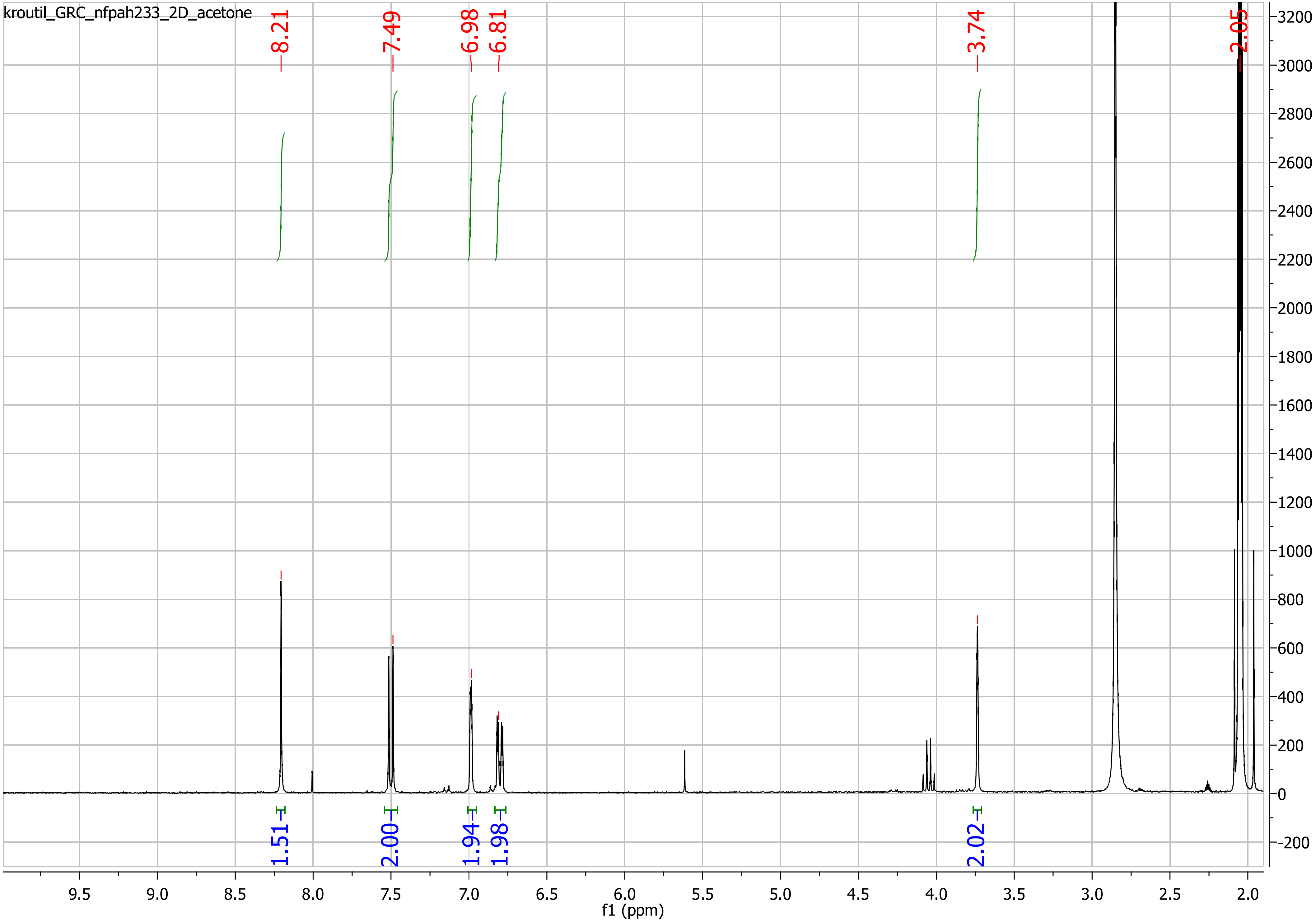

kroutil_GRC_nfpah233_2D_acetone
8.21
7.49
6.98
6.81
3.74
2.05
1.51
2.00
1.94
1.98
2.02
f1 (ppm)

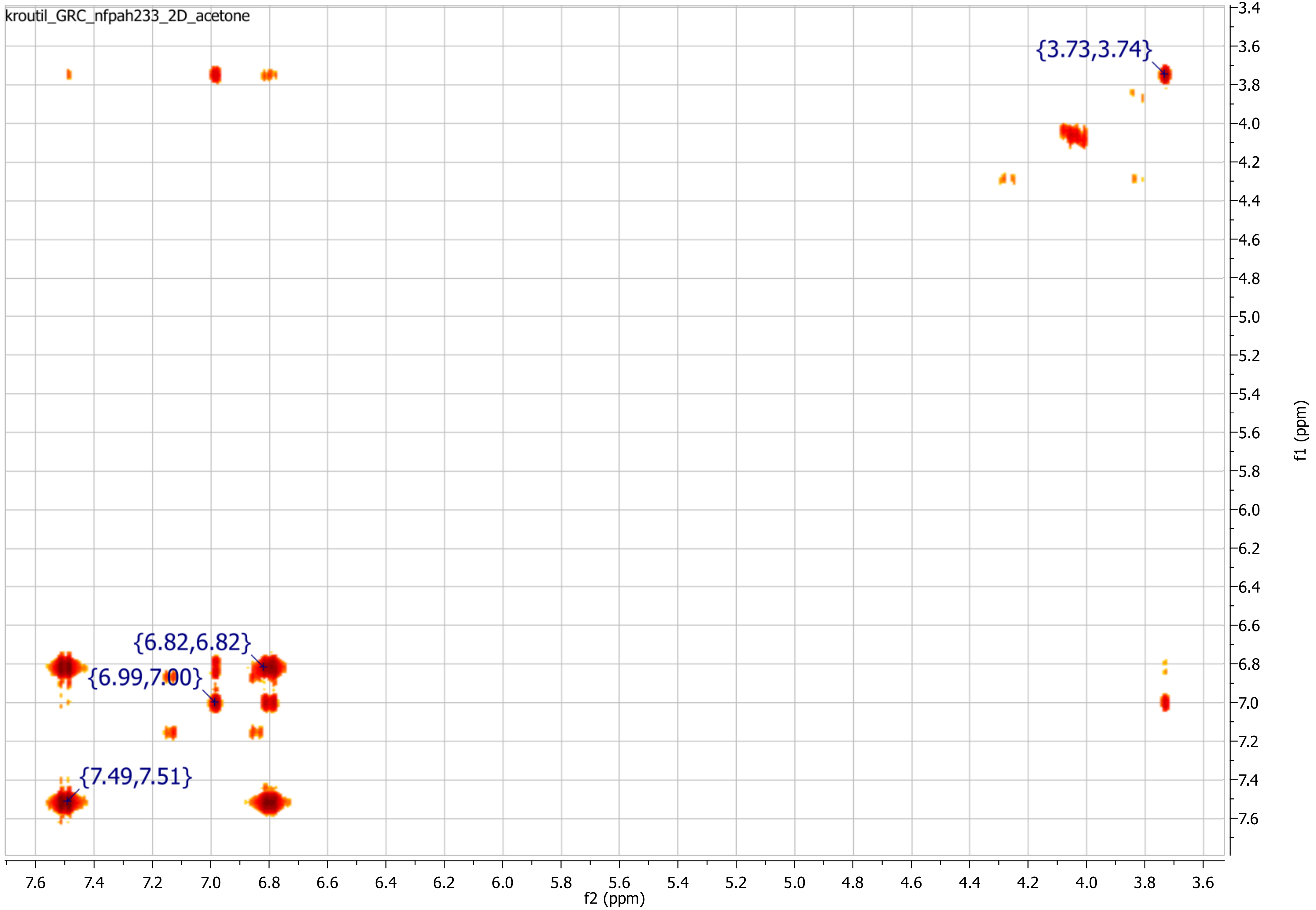
kroutil_GRC_nfpah233_2D_acetone
{3.73,3.74}
{6.82,6.82}
{6.99,7.00}
{7.49,7.51}
f2 (ppm)
f1 (ppm)

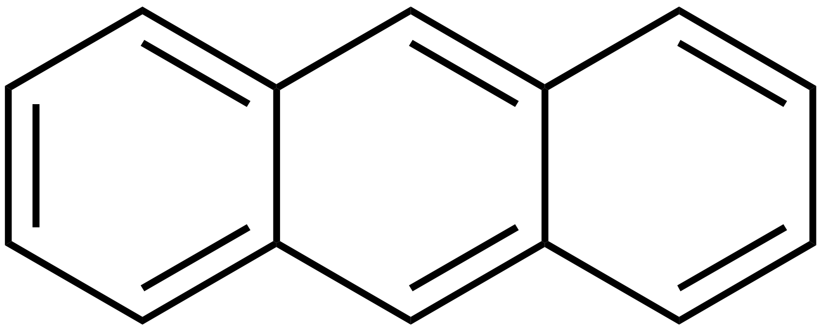

5

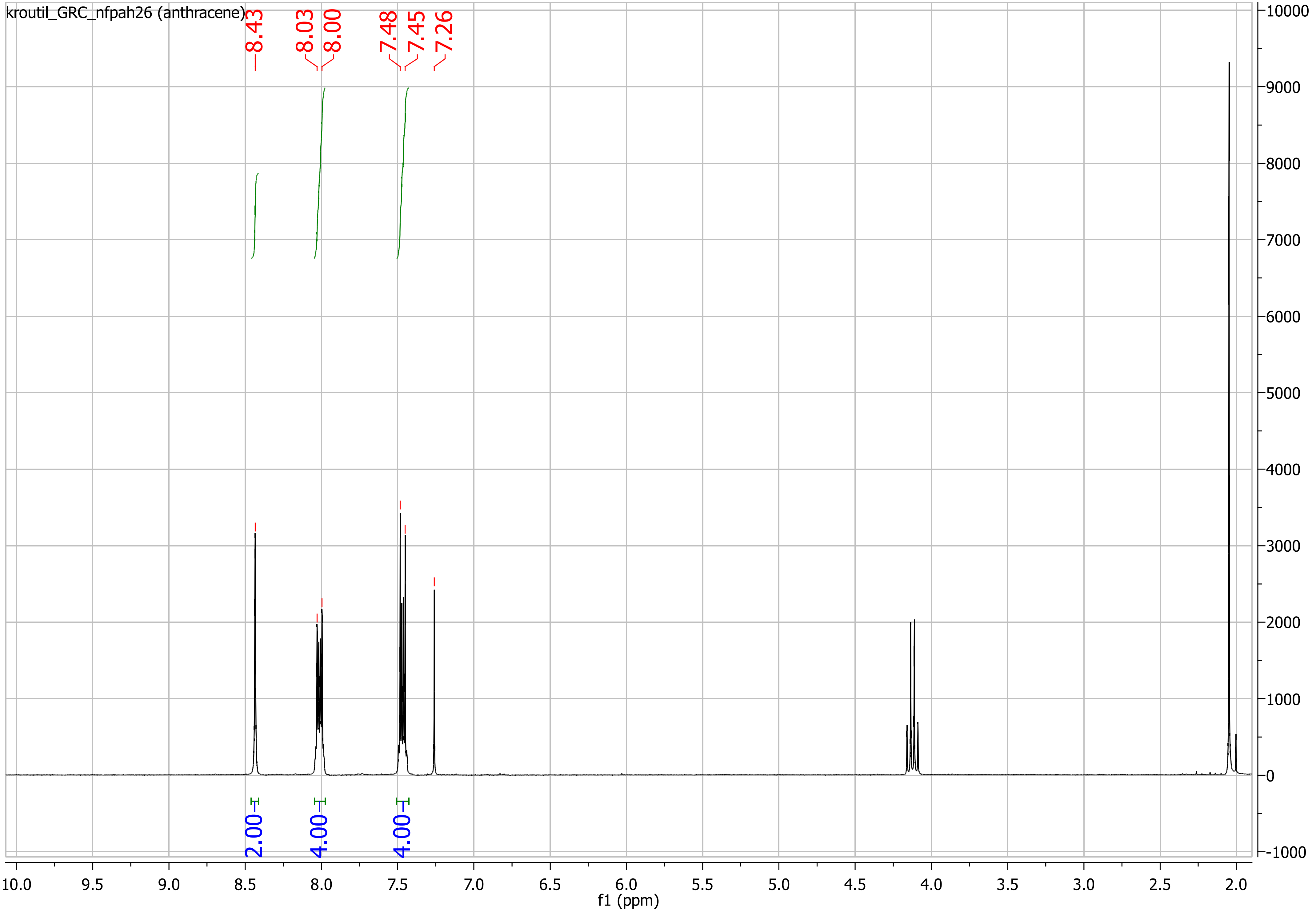

kroutil_GRC_nfpah26 (anthracene)
8.43
8.03
8.00
7.48
7.45
7.26
2.00
4.00
4.00
10000
9000
8000
7000
6000
5000
4000
3000
2000
1000
0
-1000
10.0
9.5
9.0
8.5
8.0
7.5
7.0
6.5
6.0
5.5
5.0
4.5
4.0
3.5
3.0
2.5
2.0
f1 (ppm)

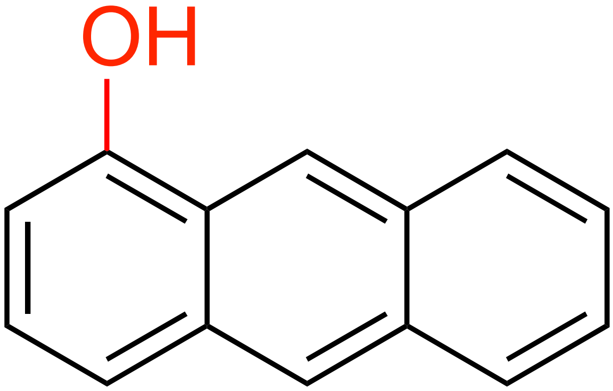

1-hydroxyanthracene (**16**)

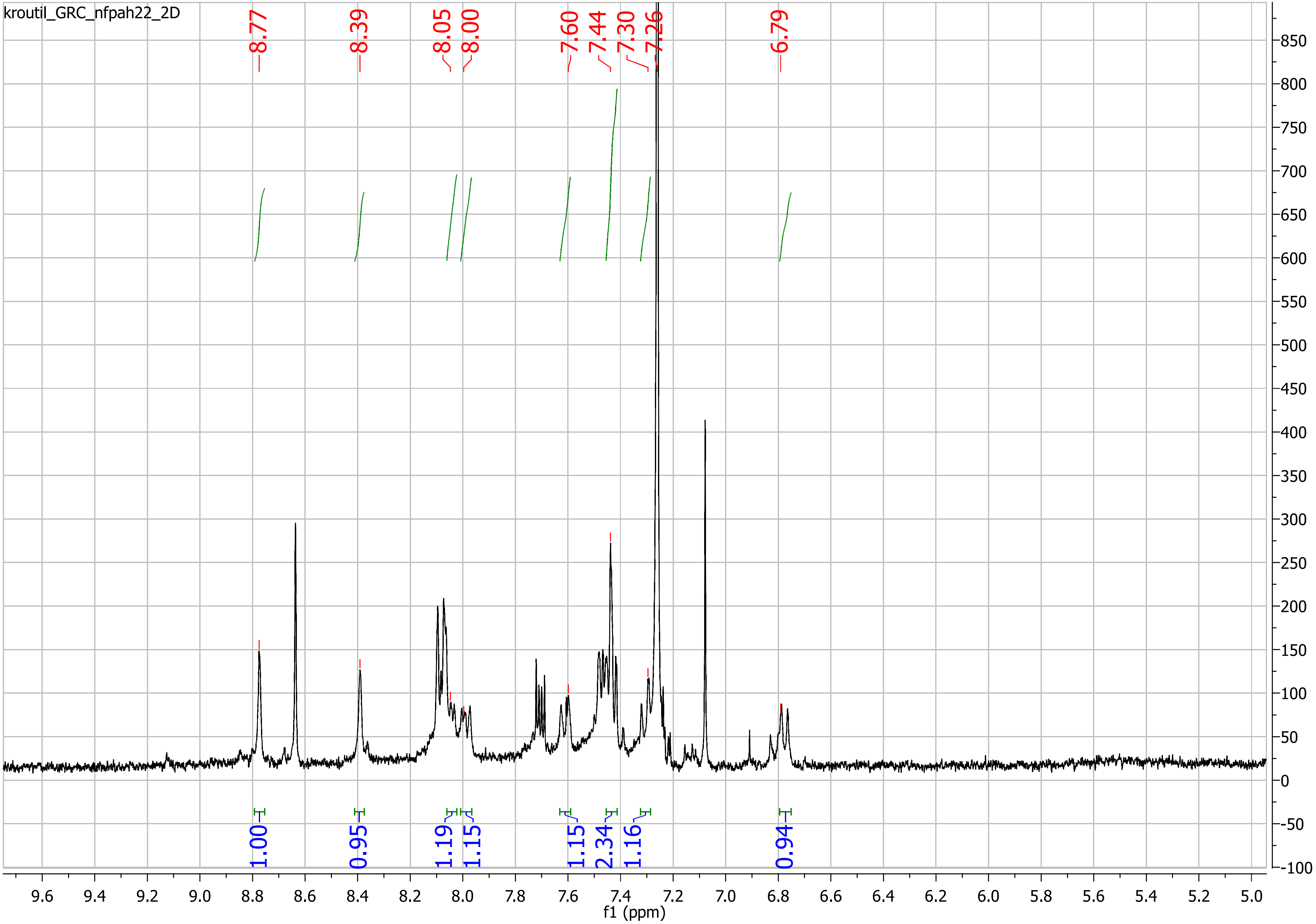

kroutil_GRC_nfpah22_2D
8.77
8.39
8.05
8.00
7.60
7.44
7.30
7.26
6.79
1.00
0.95
1.19
1.15
1.15
2.34
1.16
0.94
f1 (ppm)

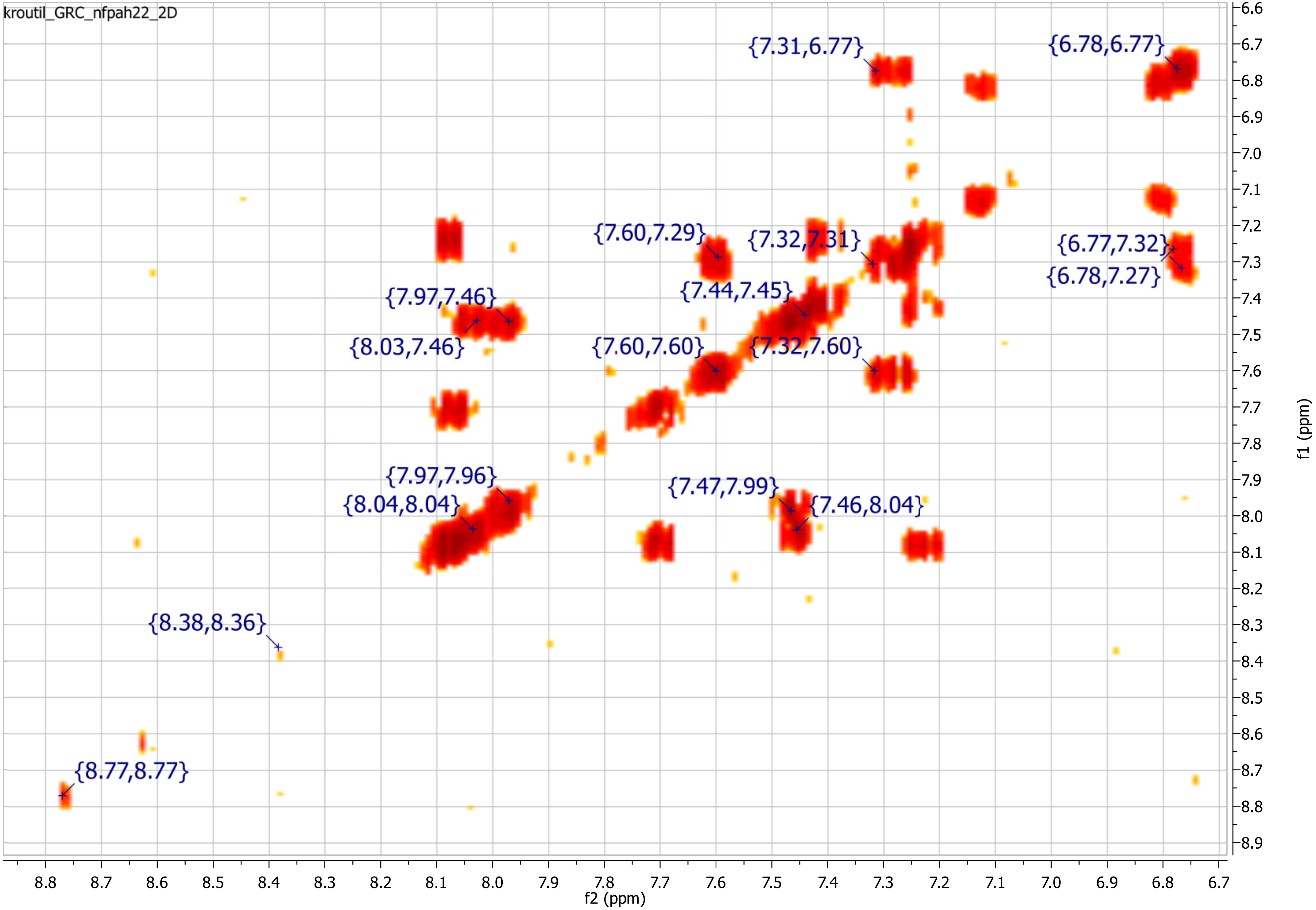
kroutil_GRC_nfpah22_2D
{7.31,6.77}
{6.78,6.77}
{7.60,7.29}
{7.32,7.31}
{6.77,7.32}
{6.78,7.27}
{7.97,7.46}
{7.44,7.45}
{8.03,7.46}
{7.60,7.60}
{7.32,7.60}
{7.97,7.96}
{7.47,7.99}
{8.04,8.04}
{7.46,8.04}
{8.38,8.36}
{8.77,8.77}
f2 (ppm)
f1 (ppm)

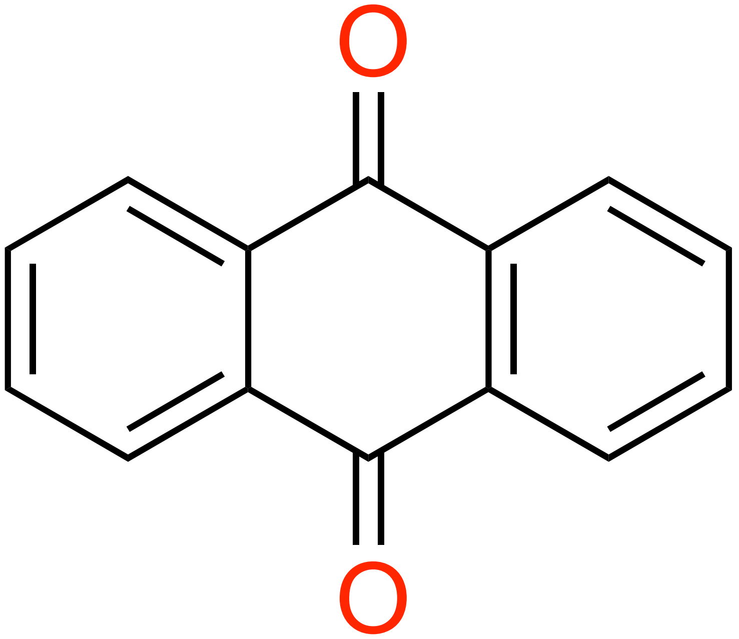

anthraquinone (**17**)

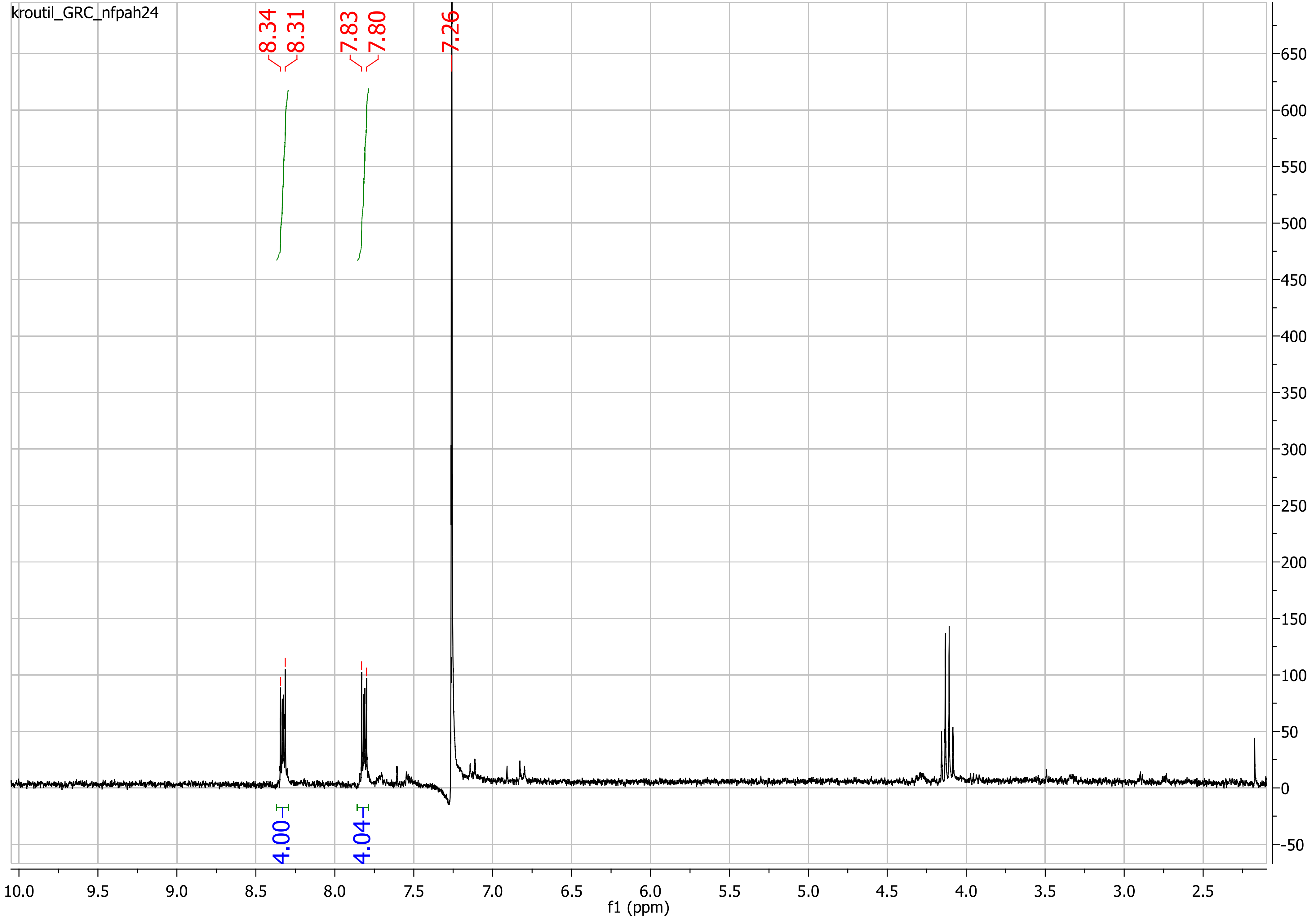

kroutil_GRC_nfpah24
8.34
8.31
7.83
7.80
7.26
4.00
4.04
f1 (ppm)

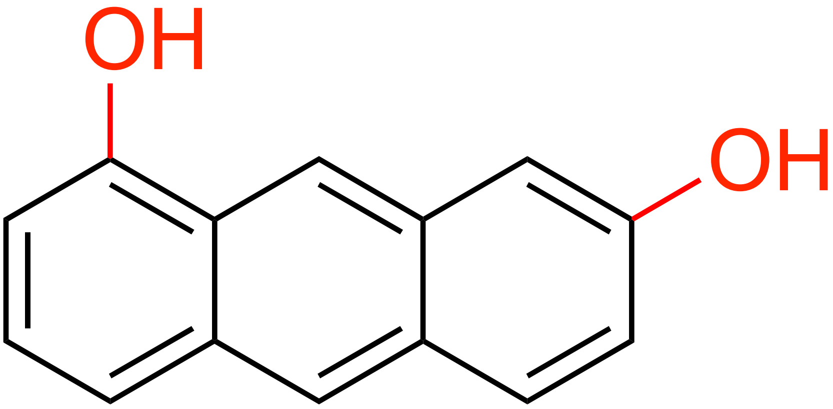

1,7-dihydroxyanthracene (**18**)

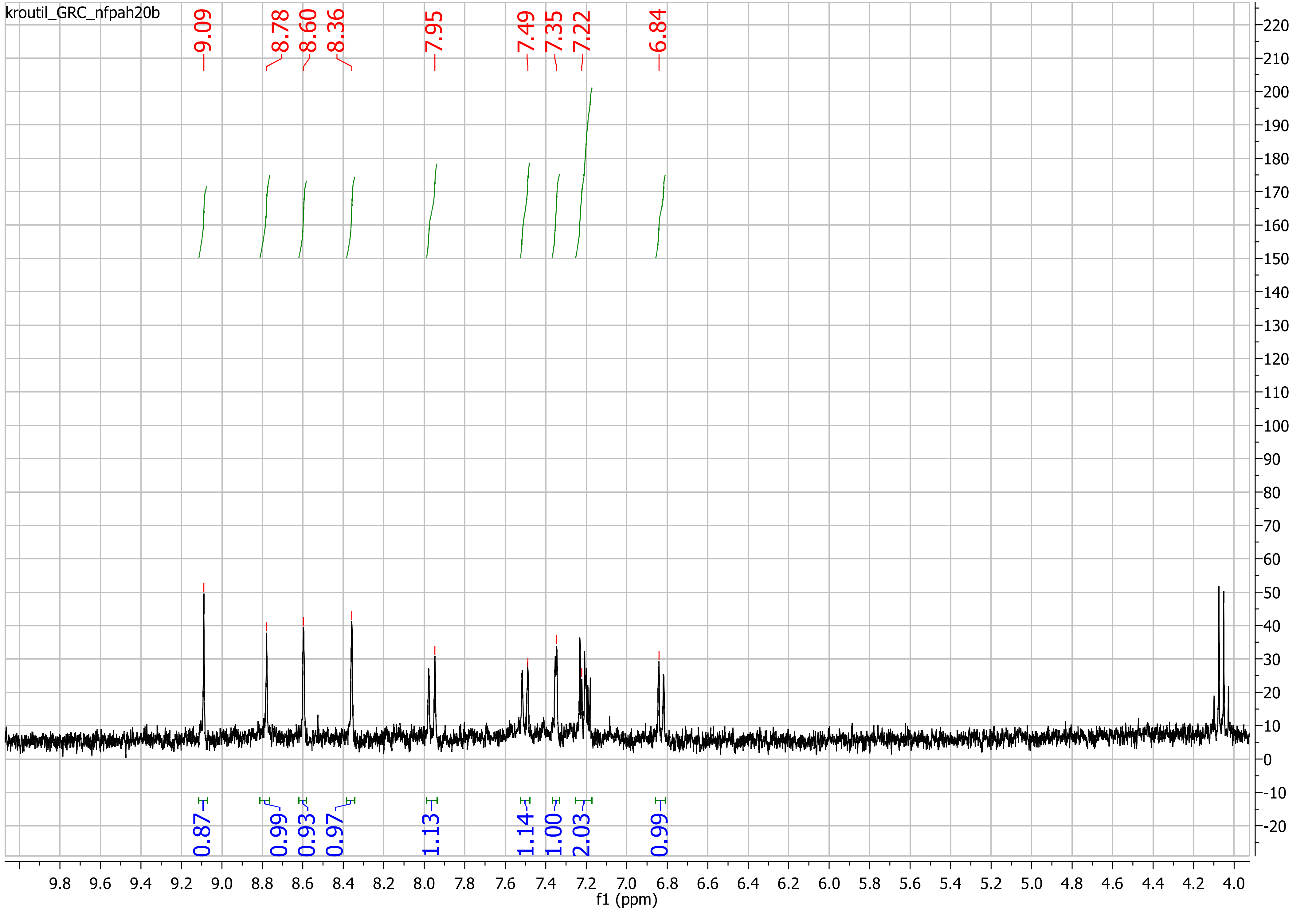

kroutil_GRC_nfpah20b
9.09
8.78
8.60
8.36
7.95
7.49
7.35
7.22
6.84
0.87
0.99
0.93
0.97
1.13
1.14
1.00
2.03
0.99
f1 (ppm)

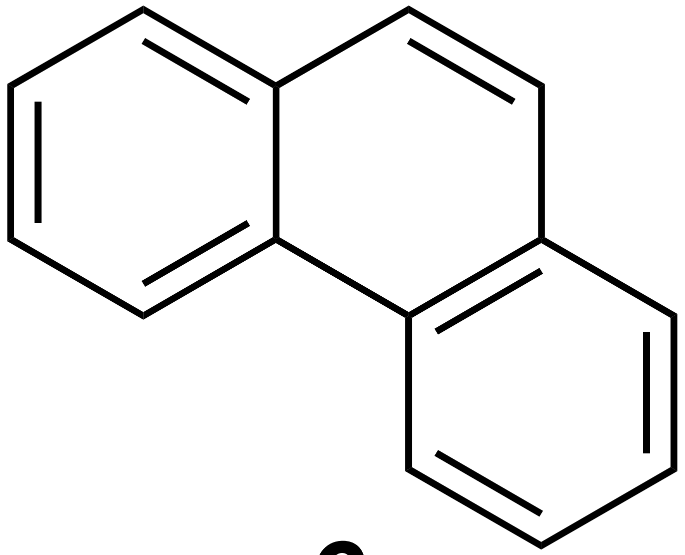

6

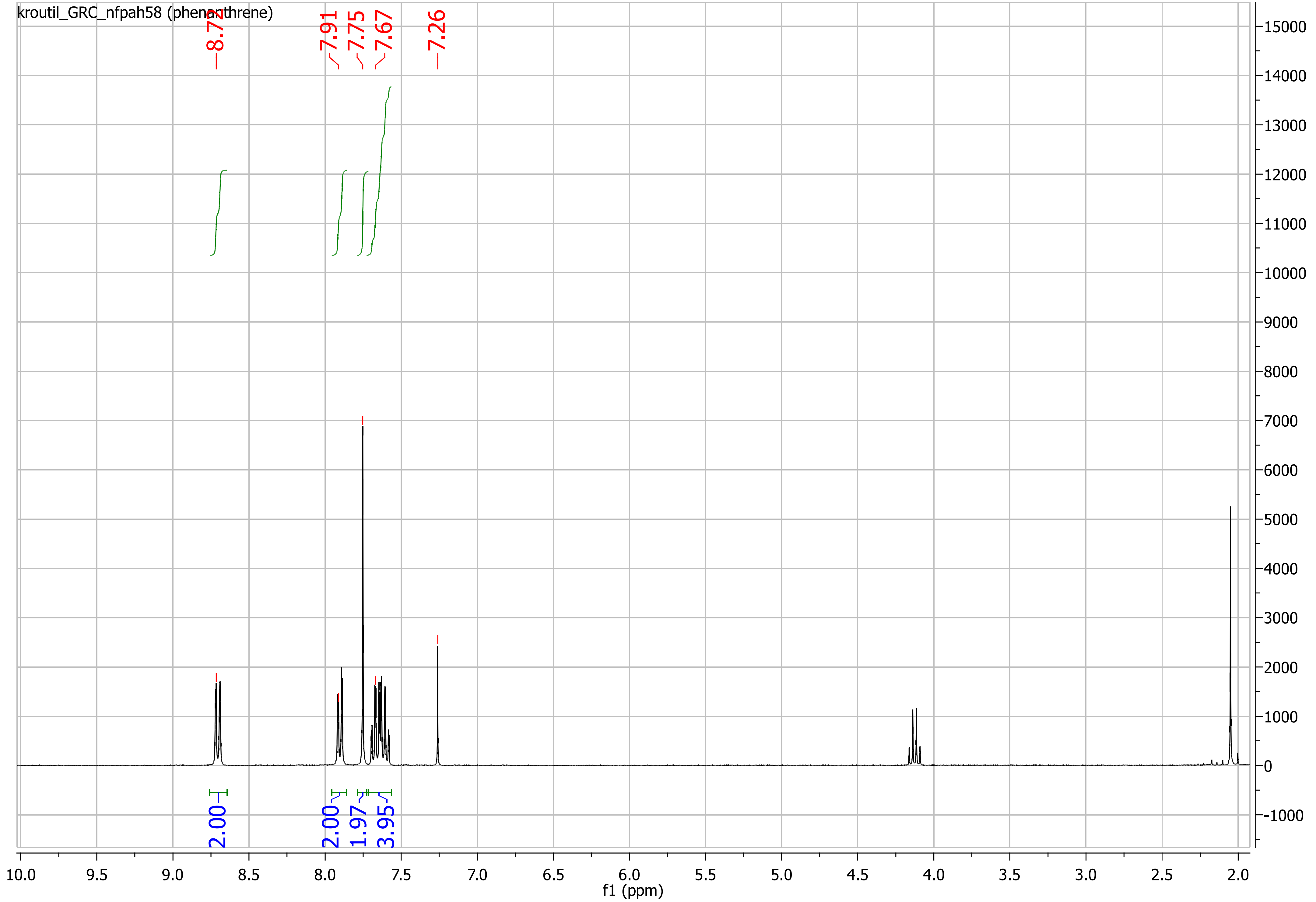
kroutil_GRC_nfpah58 (phenanthrene)
8.72
7.91
7.75
7.67
7.26
2.00
2.00
1.97
3.95
f1 (ppm)

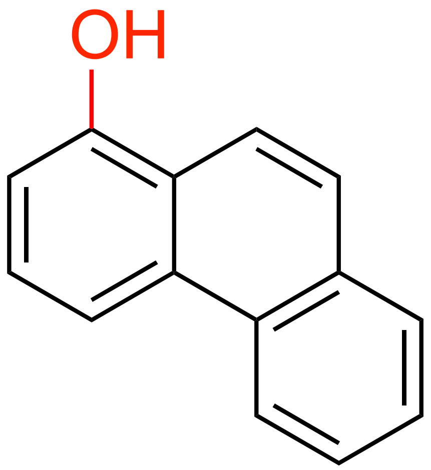

1-hydroxyphenanthrene (**19**)

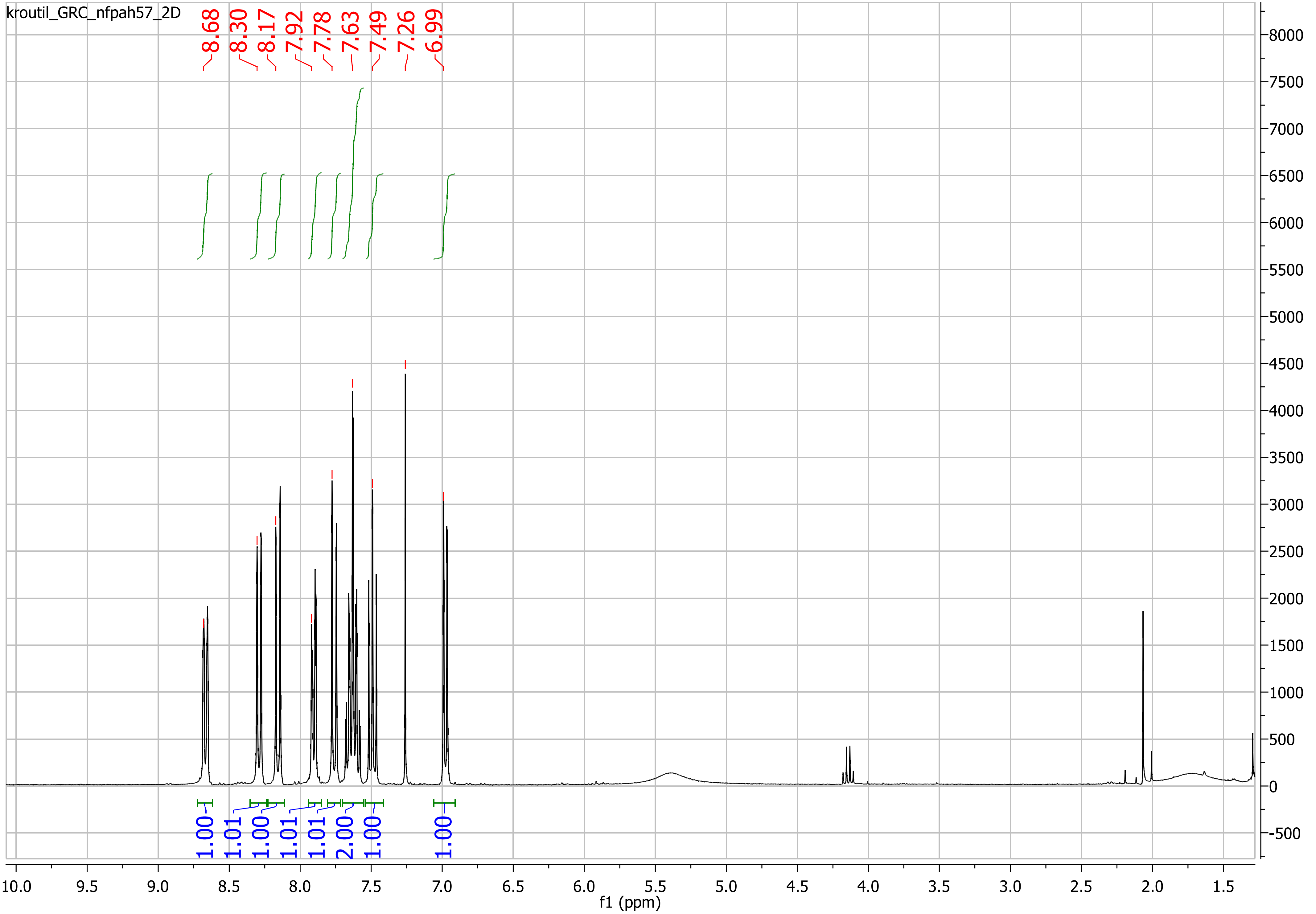
kroutil_GRC_nfpah57_2D
8.68
8.30
8.17
7.92
7.78
7.63
7.49
7.26
6.99
1.00
1.01
1.00
1.01
1.01
2.00
1.00
1.00
f1 (ppm)

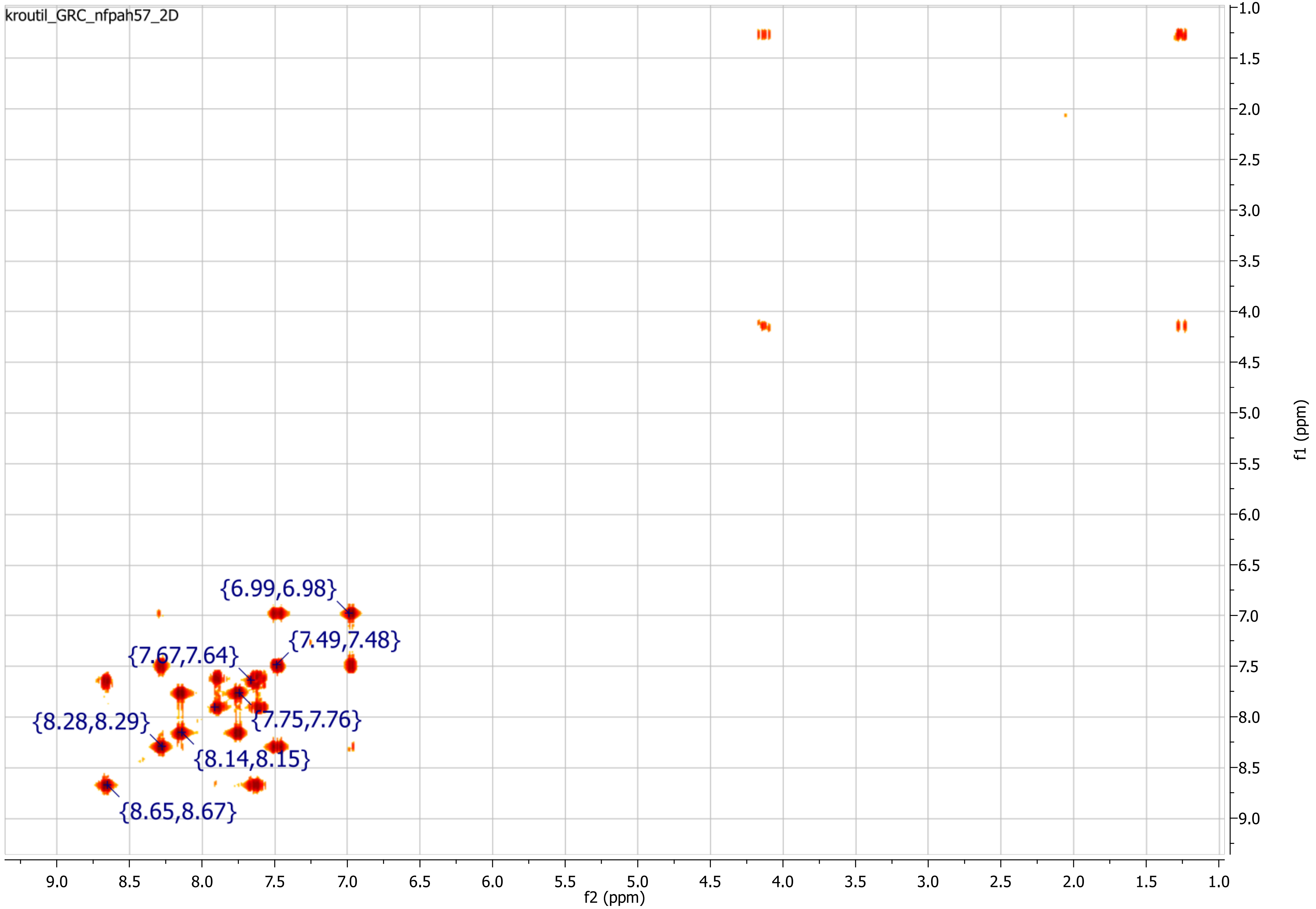

kroutil_GRC_nfpah57_2D
{6.99,6.98}
{7.49,7.48}
{7.67,7.64}
{7.75,7.76}
{8.28,8.29}
{8.14,8.15}
{8.65,8.67}
f2 (ppm)
f1 (ppm)

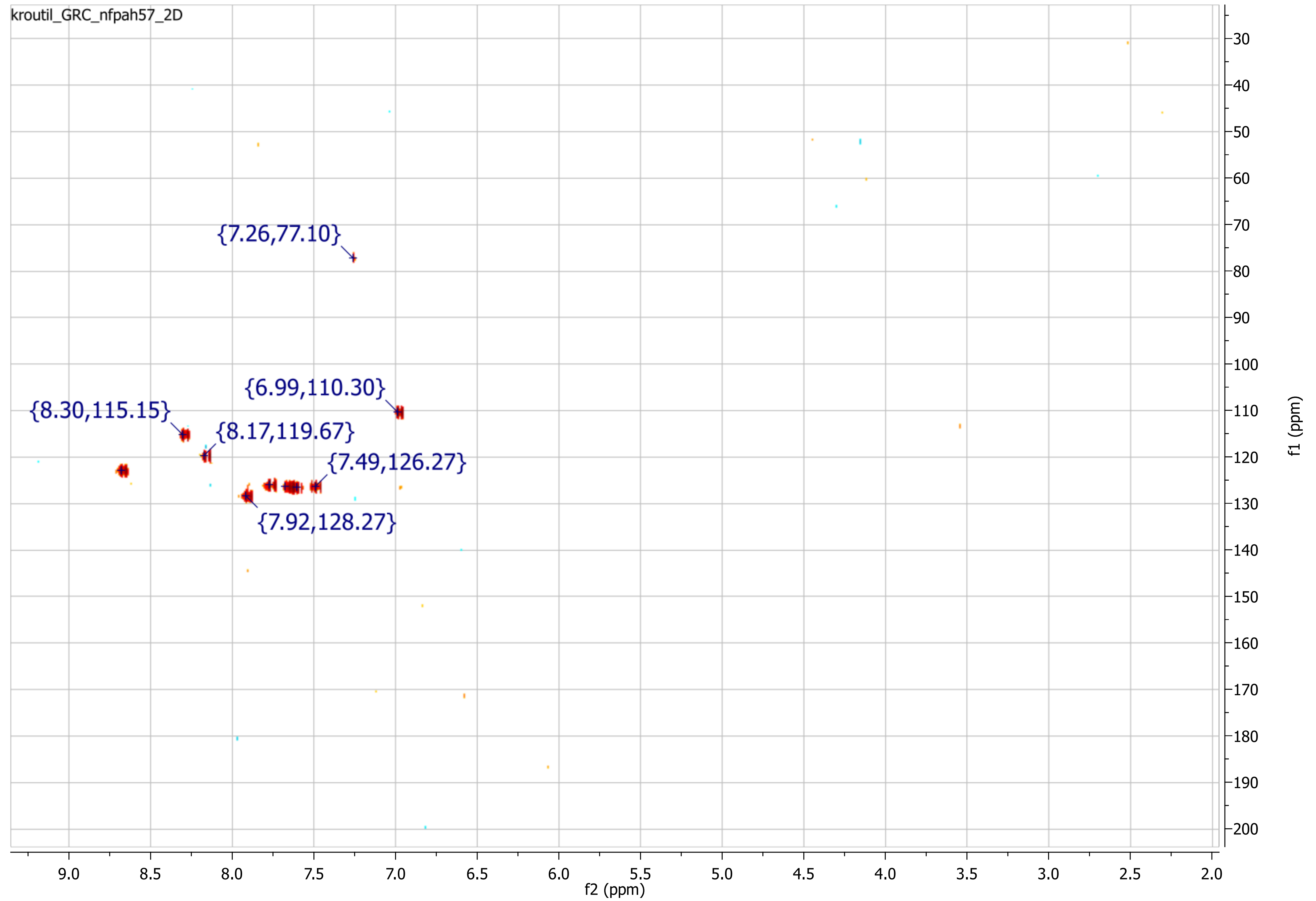

kroutil_GRC_nfpah57_2D
{7.26,77.10}
{6.99,110.30}
{8.30,115.15}
{8.17,119.67}
{7.49,126.27}
{7.92,128.27}
f2 (ppm)
f1 (ppm)

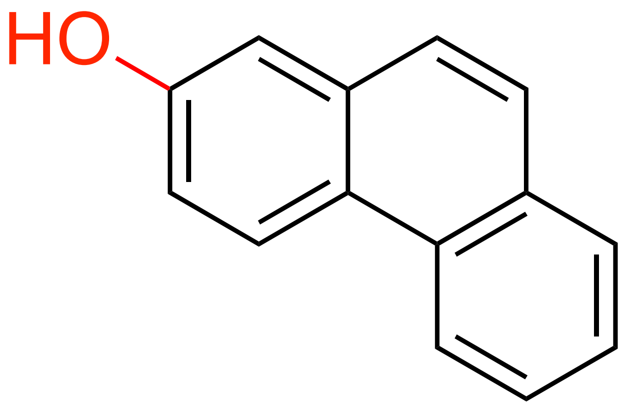

2-hydroxyphenanthrene (**20**)

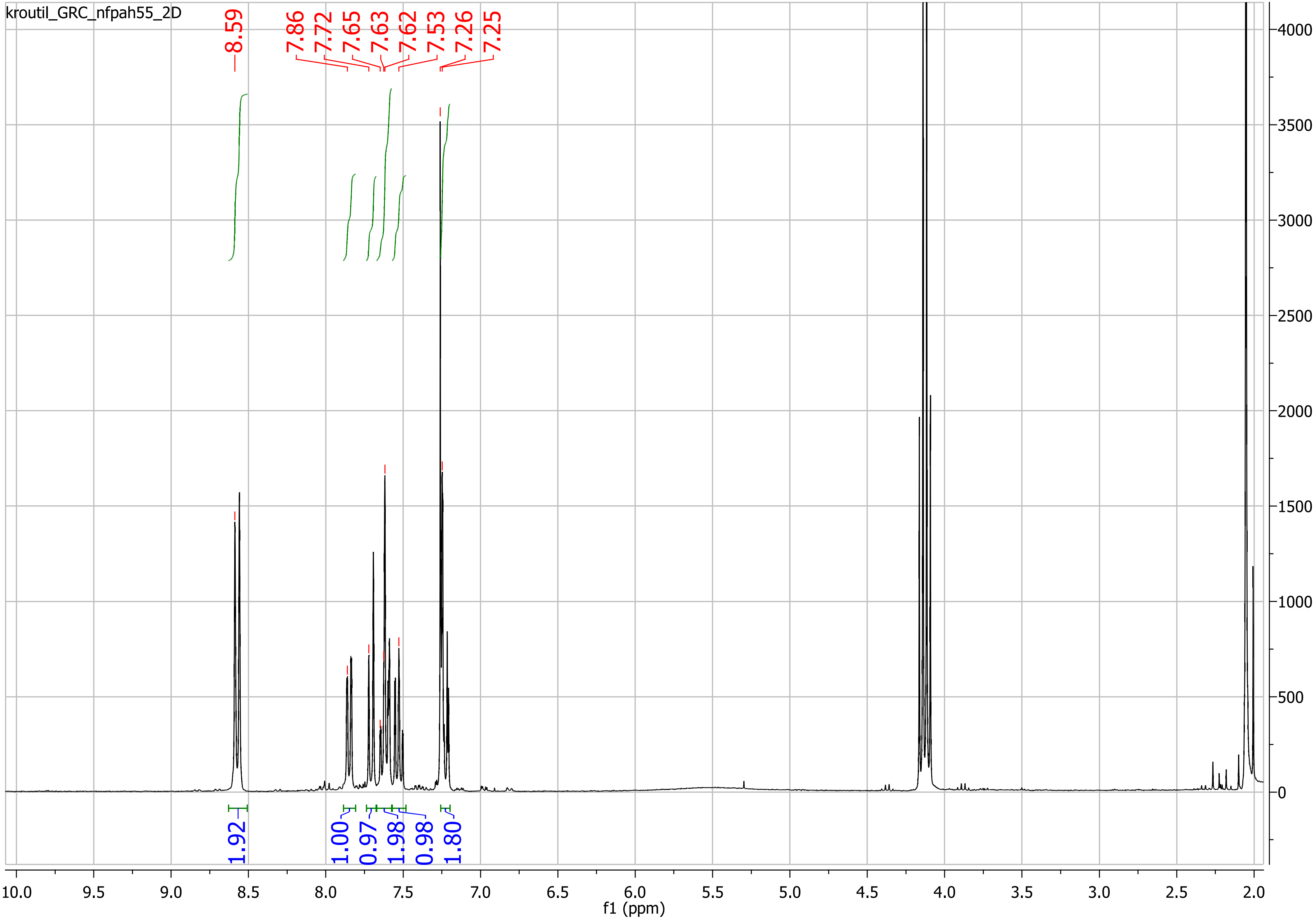

kroutil_GRC_nfpah55_2D
8.59
7.86
7.72
7.65
7.63
7.62
7.53
7.26
7.25
1.92
1.00
0.97
1.98
0.98
1.80
f1 (ppm)

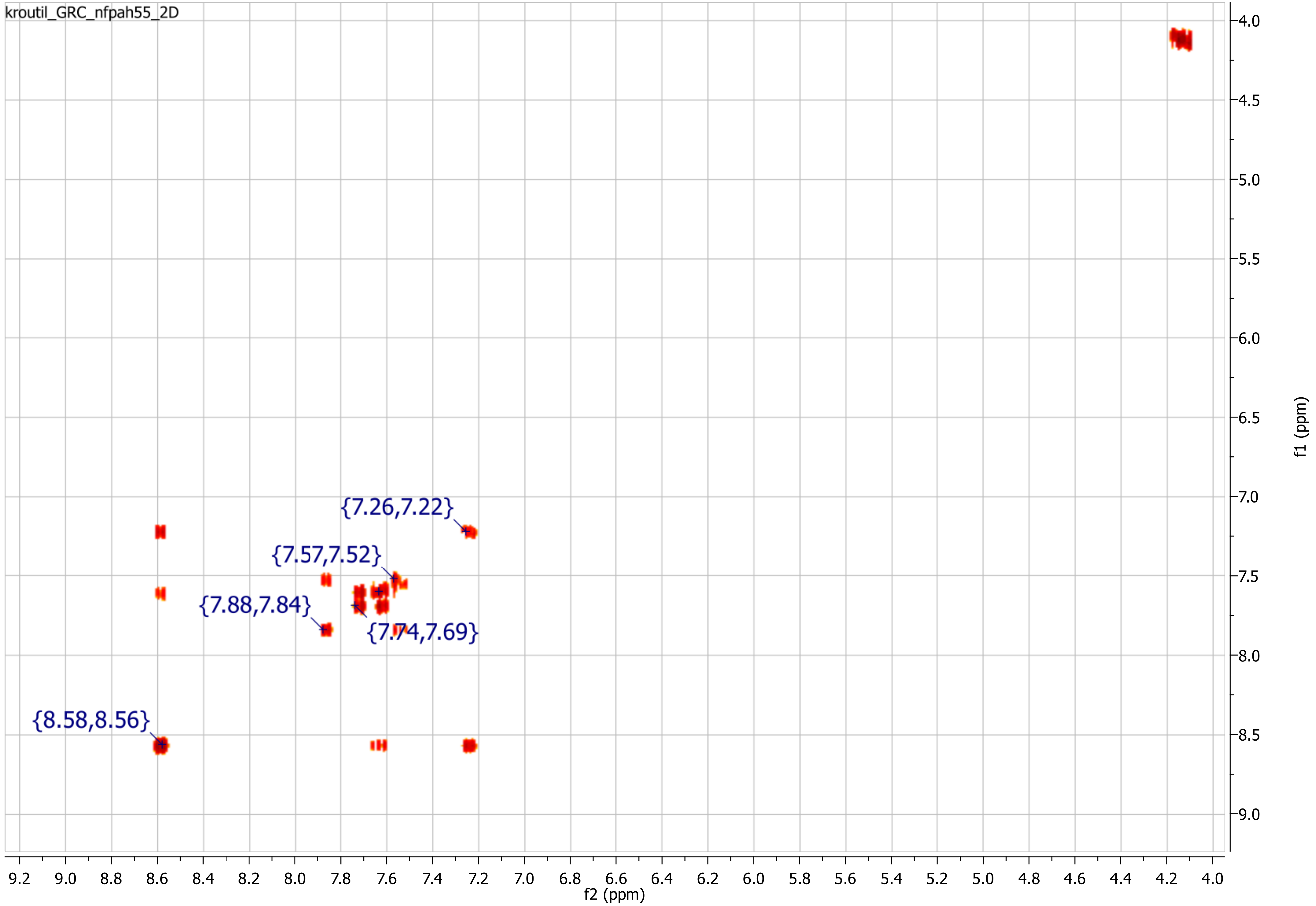

kroutil_GRC_nfpah55_2D
{7.26,7.22}
{7.57,7.52}
{7.88,7.84}
{7.74,7.69}
{8.58,8.56}
f2 (ppm)
f1 (ppm)

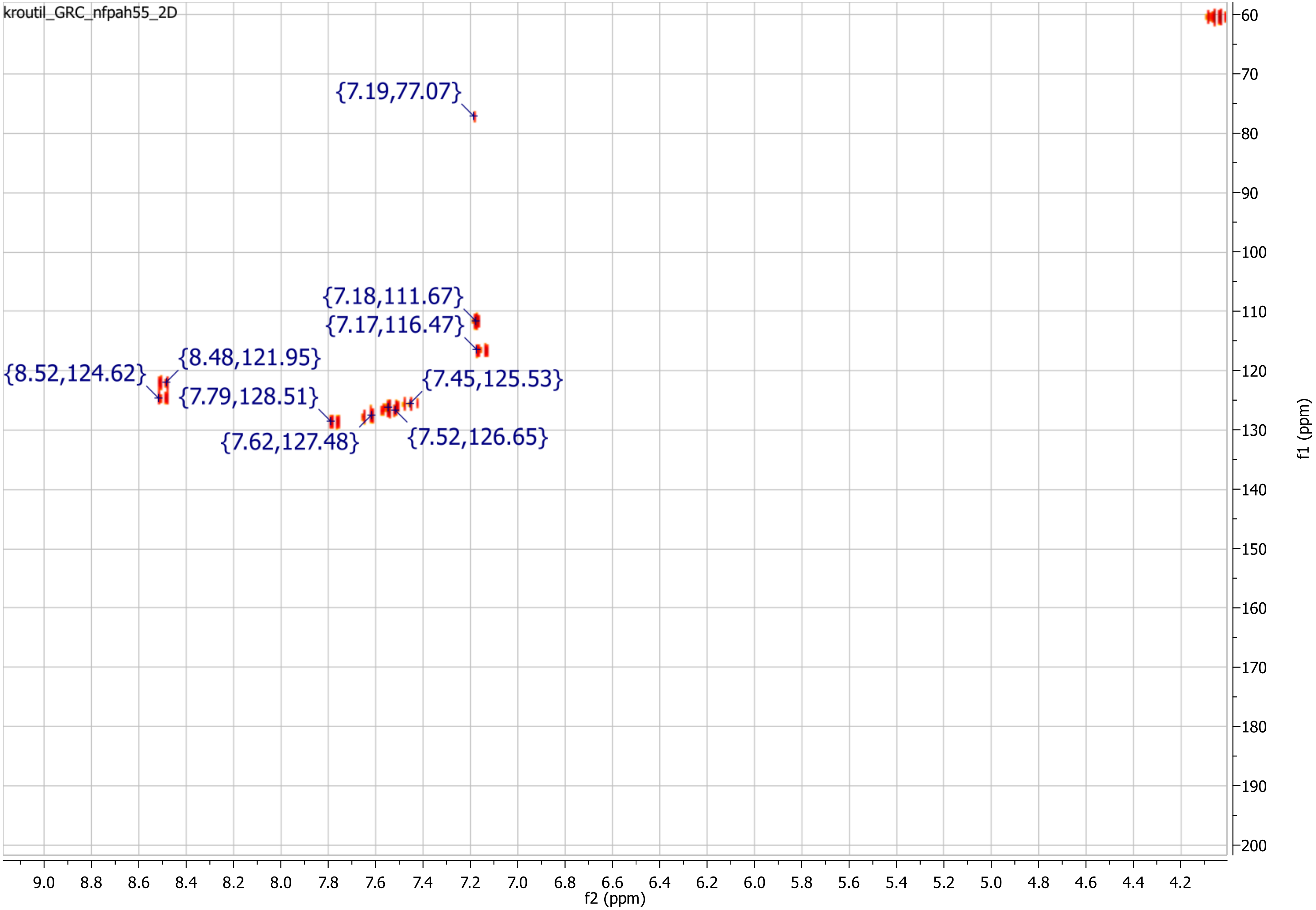
kroutil_GRC_nfpah55_2D
{7.19,77.07}
{7.18,111.67}
{7.17,116.47}
{8.48,121.95}
{8.52,124.62}
{7.79,128.51}
{7.45,125.53}
{7.62,127.48}
{7.52,126.65}
f2 (ppm)
f1 (ppm)

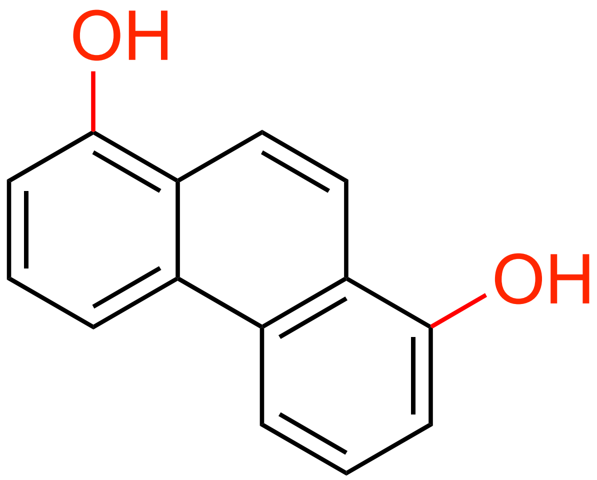

1,2-dihydroxyphenanthrene (**21**)

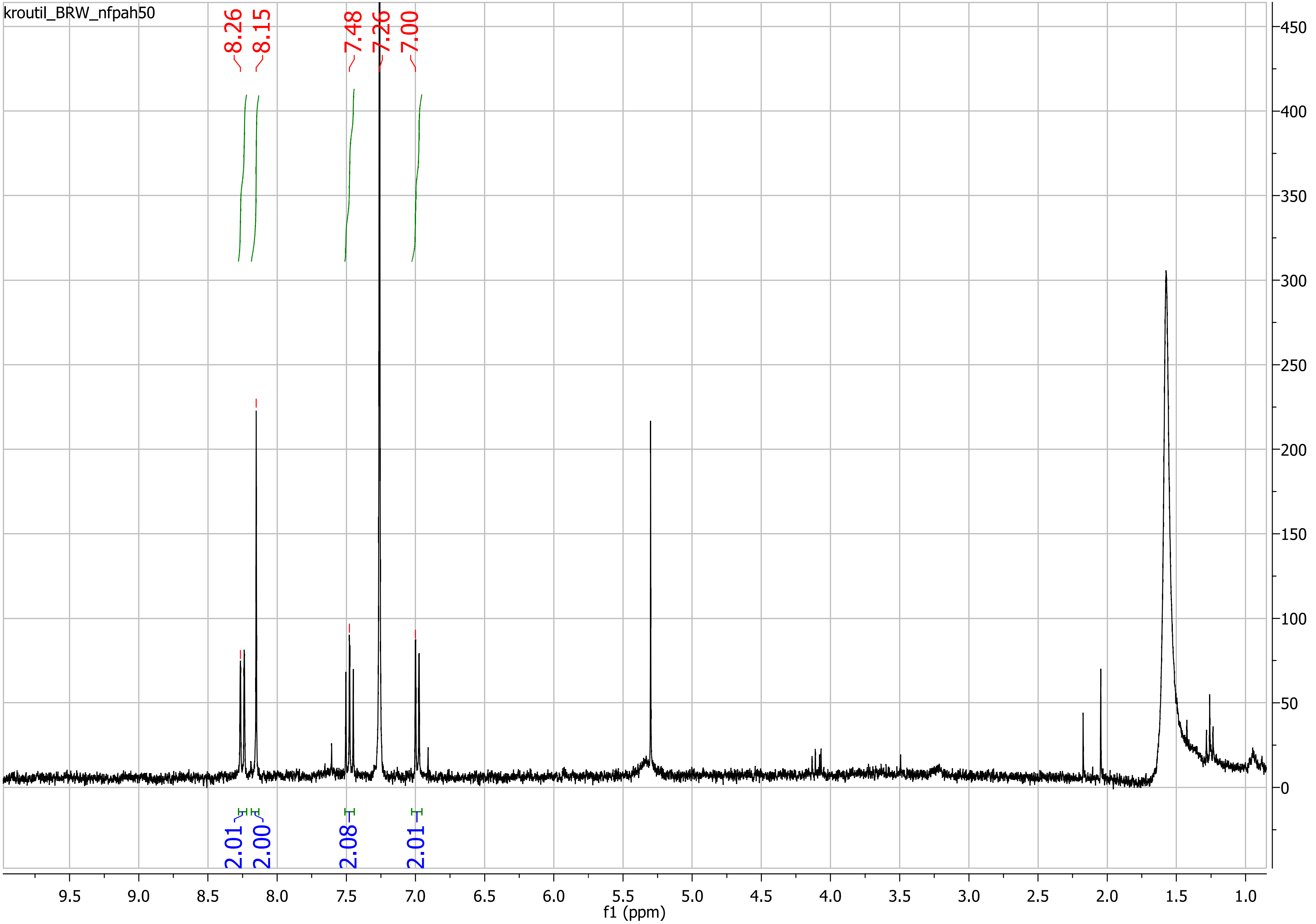

kroutil_BRW_nfpah50
8.26
8.15
7.48
7.26
7.00
2.01
2.00
2.08
2.01
450
400
350
300
250
200
150
100
50
0
9.5
9.0
8.5
8.0
7.5
7.0
6.5
6.0
5.5
5.0
4.5
4.0
3.5
3.0
2.5
2.0
1.5
1.0
f1 (ppm)

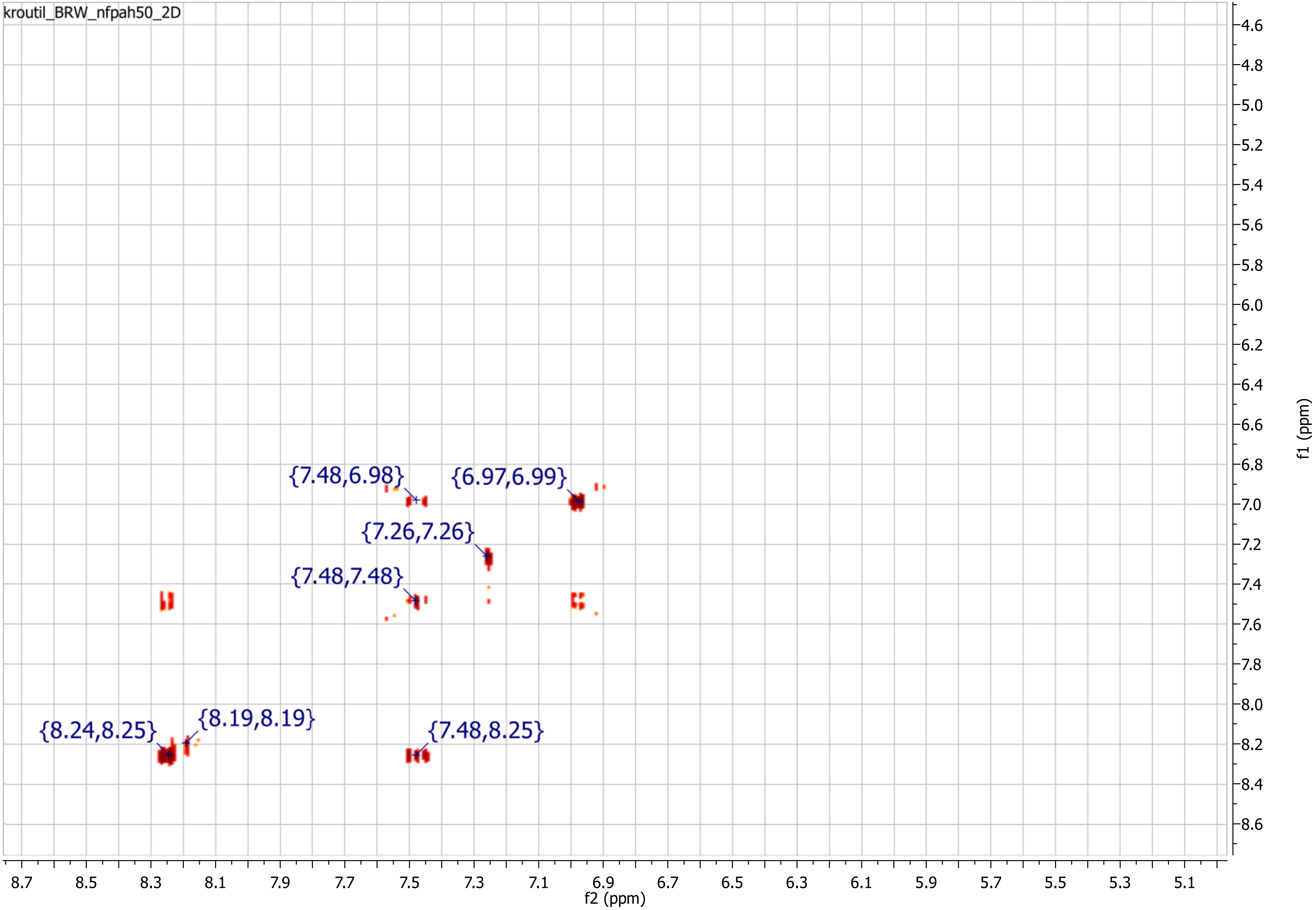

kroutil_BRW_nfpah50_2D
{7.48,6.98}
{6.97,6.99}
{7.26,7.26}
{7.48,7.48}
{8.24,8.25}
{8.19,8.19}
{7.48,8.25}
f2 (ppm)
f1 (ppm)

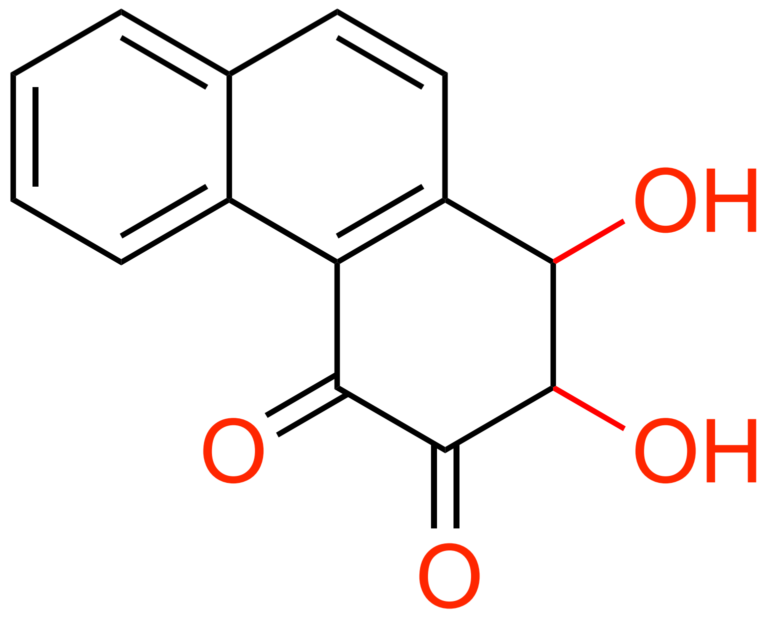

X

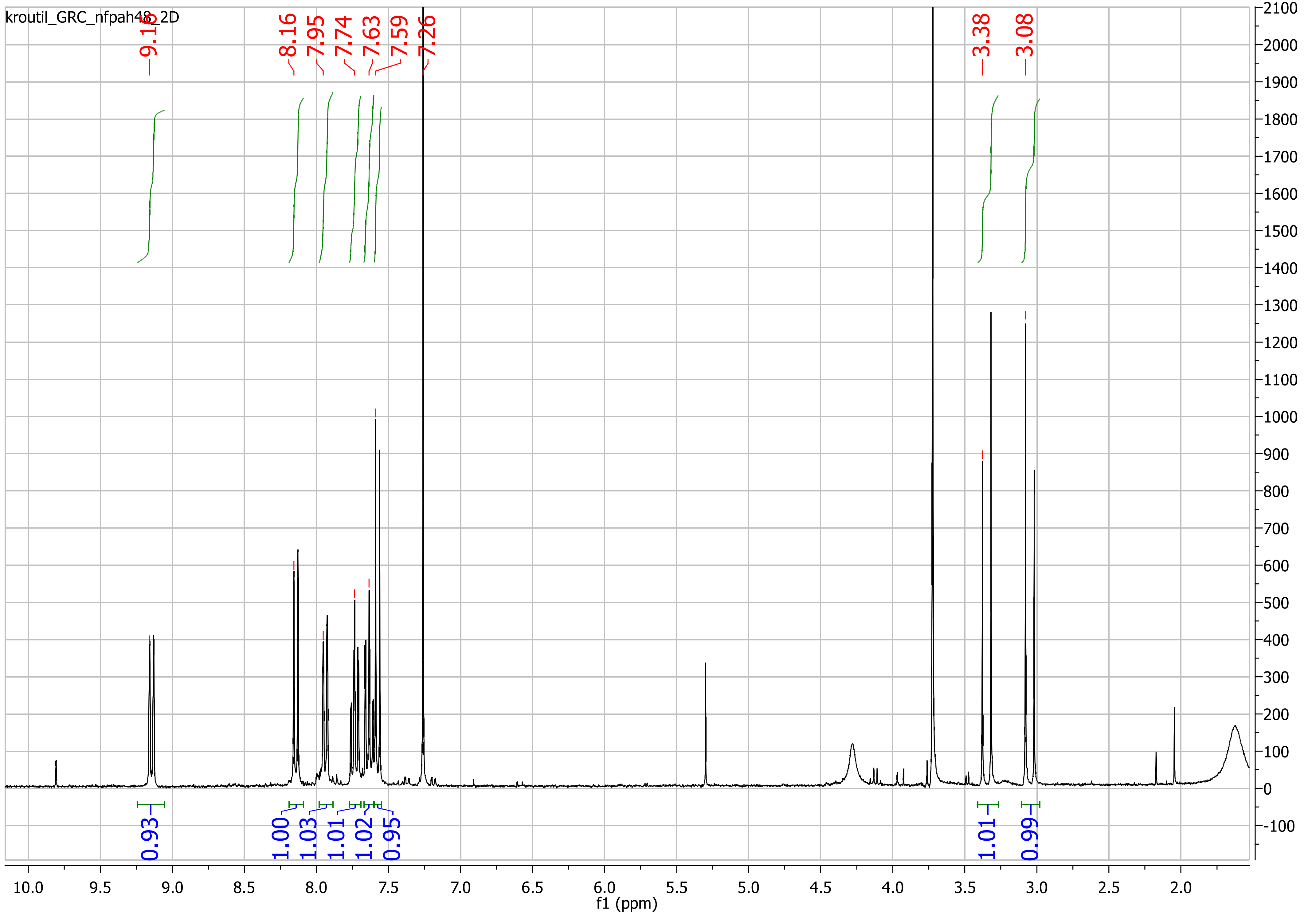

kroutil_GRC_nfpah48_2D
9.16
8.16
7.95
7.74
7.63
7.59
7.26
3.38
3.08
0.93
1.00
1.03
1.01
1.02
0.95
1.01
0.99
f1 (ppm)

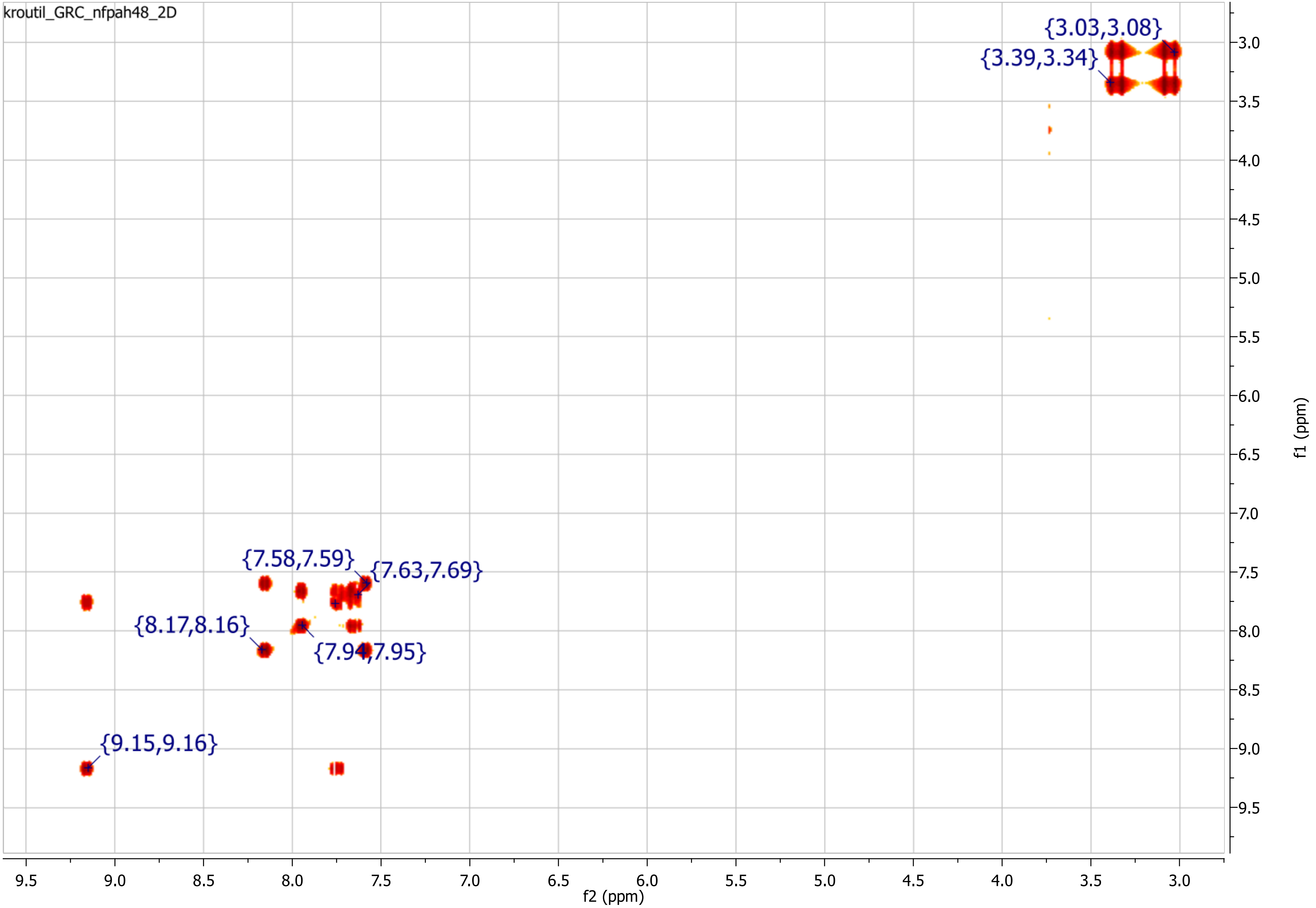
kroutil_GRC_nfpah48_2D
{3.03,3.08}
{3.39,3.34}
{7.58,7.59}
{7.63,7.69}
{8.17,8.16}
{7.94,7.95}
{9.15,9.16}
f2 (ppm)
f1 (ppm)
